# Correction: Genome editing of an African elite rice variety confers resistance against endemic and emerging *Xanthomonas oryzae* pv. *oryzae* strains

**DOI:** 10.7554/eLife.105903

**Published:** 2025-01-07

**Authors:** Van Schepler-Luu, Coline Sciallano, Melissa Stiebner, Chonghui Ji, Gabriel Boulard, Amadou Diallo, Florence Auguy, Si Nian Char, Yugander Arra, Kyrylo Schenstnyi, Marcel Buchholzer, Eliza PI Loo, Atugonza L Bilaro, David Lihepanyama, Mohammed Mkuya, Rosemary Murori, Ricardo Oliva, Sebastien Cunnac, Bing Yang, Boris Szurek, Wolf B Frommer

**Keywords:** Other

Schepler-Luu V, Sciallano C, Stiebner M, Ji C, Boulard G, Diallo A, Auguy F, Char SN, Arra Y, Schenstnyi K, Buchholzer M, Loo EPI, Bilaro AL, Lihepanyama D, Mkuya M, Murori R, Oliva R, Cunnac S, Yang B, Szurek B, Frommer WB. 2023. Genome editing of an African elite rice variety confers resistance against endemic and emerging *Xanthomonas oryzae* pv. oryzae strains. *eLife* **12**:e84864. doi: 10.7554/eLife.84864.

Published 20 June 2023

In this study, Table 1 highlights the resistance status of a set of near-isogenic lines (NILs) of rice each harboring a single BB resistance gene, upon inoculation of 18 representative endemic *Xoo* strains from diverse African countries and 3 strains corresponding to the emerging Tanzanian population introduced from Asia. We found out that our IRBB23 seed stock was mislabelled. Seeds were ordered from the International Rice Research Institute (IRRI) and a new test was done to see how IRBB23 and the susceptible control Azucena reacted to the *Xoo* strains. This has two main consequences. One relates to strains NAI9, AXO1947, BAI3, CFBP1948, NAI5 and S82-4-3, that were previously identified as avirulent on IRBB23, and that are in fact virulent. The other deals with strains iTzDak19-1, iTzDak19-2 and iTzDak19-3, which turn out to be avirulent but not virulent on IRBB23, as we had primarily witnessed. All the other strains triggered a resistance phenotype on IRBB23 as observed previously. As the new data showed that *Xa23* shows resistance to iTzDak19 but not to some other strains from Africa, we added a sentence in the Discussion to comment on how *Xa23* could be used to manage disease caused by the emerging *Xoo* population.

Corrected text (in the Results section):

NILs harboring *Xa1*, *Xa23*, *xa5* or *Xa4* were resistant to most endemic African strains, but surprisingly, iTzDak19-1, iTzDak19-2, and iTzDak19-3 were virulent on all NILs tested, except IRBB23.

Original text:

NILs harboring *Xa1*, *Xa23*, *xa5* or *Xa4* were resistant to most endemic African strains, but surprisingly, iTzDak19-1, iTzDak19-2, and iTzDak19-3 were virulent on all NILs tested.

Corrected Table 1:

Efficiency of resistance genes in IRBB rice lines towards a diversity panel of 26 African *Xoo* strains.

**Table:** 

Strain Name	CIX code	Country	Azucena	IR24	IRBB1	IRBB3	IRBB4	IRBB5	IRBB7	IRBB23	Komboka
NatiPark	607	Benin	S	R	R	R	R	R	R	R	R
Karfiguela13	705	Burkina Faso	S	R	R	R	R	R	R	R	R
N2-4	4482	Niger	S	R	R	R	R	R	R	R	R
Tanguieta3	609	Benin	S	MR	R	MR	R	R	MR	R	R
Toula20	629	Niger	S	MR	R	MR	R	R	MR	MR	R
BAI250	4127	Burkina Faso	S	MR	R	MR	MR	R	R	MR	R
S62-2-22	2374	Senegal	S	MR	R	MS	R	R	MR	R	R
CII-1	4083	Ivory Coast	S	MS	MR	MS	R	R	MR	MR	R
CII-2	1042	Ivory Coast	S	MS	MR	MS	MR	R	MR	MR	R
NAI9	2787	Niger	S	MS	MR	MS	MR	MR	MS	S	R
AXO1947	1917	Cameroon	S	S	R	S	R	R	MS	MS	R
MAI145	894	Mali	S	S	R	S	MR	MS	S	MR	R
BAI3	4092	Burkina Faso	S	MS	MR	S	MR	MR	MS	S	R
CFBP1948	2801	Cameroon	S	MS	MR	S	MR	MR	S	S	R
NAI5	4099	Niger	S	S	MR	S	MR	MR	S	S	R
MAI73	4079	Mali	S	S	R	S	MS	MR	S	MR	R
S82-4-3	2976	Senegal	S	MS	MR	S	MS	MR	MS	S	R
MAI132	4517	Mali	S	S	R	S	S	MS	S	MR	R
iTzDak19-1	4457	Tanzania	S	S	S	S	S	S	S	R	S
iTzDak19-2	4458	Tanzania	S	S	S	S	S	S	S	R	S
iTzDak19-3	4462	Tanzania	S	S	S	S	S	S	S	R	S
iTzLuk21-1	4506	Tanzania	nd	nd	nd	nd	nd	nd	nd	nd	nd
iTzLuk21-2	4509	Tanzania	nd	nd	nd	nd	nd	nd	nd	nd	nd
iTzLuk21-3	4505	Tanzania	nd	nd	nd	nd	nd	nd	nd	nd	S
iTzLuk21-4	4507	Tanzania	nd	nd	nd	nd	nd	nd	nd	nd	S
iTzLuk21-5	4508	Tanzania	nd	nd	nd	nd	nd	nd	nd	nd	nd

n.d. not determined

The originally published version of Table 1 is also shown for reference:

Efficiency of resistance genes in IRBB rice lines towards a diversity panel of 26 African *Xoo* strains.

**Table:** 

Strain Name	CIX code	Country	Azucena	IR24	IRBB1	IRBB3	IRBB4	IRBB5	IRBB7	IRBB23	Komboka
NatiPark	607	Benin	S	**R**	**R**	**R**	**R**	**R**	**R**	**R**	**R**
Karfiguela13	705	Burkina Faso	S	**R**	**R**	**R**	**R**	**R**	**R**	**R**	**R**
N2-4	4482	Niger	S	**R**	**R**	**R**	**R**	**R**	**R**	**R**	**R**
Tanguieta3	609	Benin	S	MR	**R**	MR	**R**	**R**	MR	**R**	**R**
Toula20	629	Niger	S	MR	**R**	MR	**R**	**R**	MR	**R**	**R**
BAI250	4127	Burkina Faso	S	MR	**R**	MR	MR	**R**	**R**	**R**	**R**
S62-2-22	2374	Senegal	S	MR	**R**	MS	**R**	**R**	MR	**R**	**R**
CII-1	4083	Ivory Coast	S	MS	MR	MS	**R**	**R**	MR	**R**	**R**
CII-2	1042	Ivory Coast	S	MS	MR	MS	MR	**R**	MR	**R**	**R**
NAI9	2787	Niger	S	MS	MR	MS	MR	MR	MS	**R**	**R**
AXO1947	1917	Cameroon	S	S	**R**	S	**R**	**R**	MS	**R**	**R**
MAI145	894	Mali	S	S	**R**	S	MR	MS	S	**R**	**R**
BAI3	4092	Burkina Faso	S	MS	MR	S	MR	MR	MS	**R**	**R**
CFBP1948	2801	Cameroon	S	MS	MR	S	MR	MR	S	**R**	**R**
NAI5	4099	Niger	S	S	MR	S	MR	MR	S	**R**	**R**
MAI73	4079	Mali	S	S	**R**	S	MS	MR	S	**R**	**R**
S82-4-3	2976	Senegal	S	MS	MR	S	MS	MR	MS	**R**	**R**
MAI132	4517	Mali	S	S	R	S	S	MS	S	**R**	**R**
iTzDak19-1	4457	Tanzania	S	S	S	S	S	S	S	S	S
iTzDak19-2	4458	Tanzania	S	S	S	S	S	S	S	S	S
iTzDak19-3	4462	Tanzania	S	S	S	S	S	S	S	S	S
iTzLuk21-1	4506	Tanzania	nd	nd	nd	nd	nd	nd	nd	nd	nd
iTzLuk21-2	4509	Tanzania	nd	nd	nd	nd	nd	nd	nd	nd	nd
iTzLuk21-3	4505	Tanzania	nd	nd	nd	nd	nd	nd	nd	nd	S
iTzLuk21-4	4507	Tanzania	nd	nd	nd	nd	nd	nd	nd	nd	S
iTzLuk21-5	4508	Tanzania	nd	nd	nd	nd	nd	nd	nd	nd	nd

n.d. not determined

Corrected text (in the Discussion section):

Due to the distinct evolutionary trace and the resulting absence of coevolution, it is likely that many African rice varieties do not contain suitable *R*-genes that protect against Asian strains. Here we identified the dominant *R*-gene *Xa23* as being effective against the iTz strains. One may thus consider to introgress into local rice varieties in East-Africa. Based on the sequence information for the iTz strains and resistance profiling of Chinese strains, a combination of *Xa21* and *xa13* might also be able to protect against the iTz strains.

Hallmark features of the new strains identified in Tanzania are the presence of iTALes and the PthXo1 homolog PthXo1B; both are absent from endemic African strains. African rice lines carrying *Xa1* may thus be resistant to endemic African strains, but will be susceptible to Asian strains, including those recently introduced to Tanzania. Surveys over several years, including preliminary data from 2022, indicate that the outbreak is gaining momentum regarding severity and spread, thus potentially becoming a threat to East Africa, and over the course of time, possibly to all of Africa.

Original text (in the Discussion section):

Due to the distinct evolutionary trace and the resulting absence of coevolution, it is likely that many African rice varieties do not contain suitable *R*-genes that protect against Asian strains. Hallmark features of the new strains identified in Tanzania are the presence of iTALes and the PthXo1 homolog PthXo1B; both are absent from endemic African strains. African rice lines carrying *Xa1* may thus be resistant to endemic African strains, but will be susceptible to Asian strains, including those recently introduced to Tanzania. Surveys over several years, including preliminary data from 2022, indicate that the outbreak is gaining momentum regarding severity and spread, thus potentially becoming a threat to East Africa, and over the course of time, possibly to all of Africa.

