# Supplementary material for: Genome editing of an African elite rice variety confers resistance against endemic and emerging Xanthomonas oryzae pv. oryzae strains
Source: eLife. 2023 Jun 20;12:e84864. doi: 10.7554/eLife.84864 (PMC10322153; doi:10.7554/eLife.84864)
Supplement: Figure 3—source data 1. — CIX4505, CIX4507, CIX4508, CIX4457, CIX4458, Xoo3-1. [file elife-84864-fig3-data1.docx]

20 40 60 80 100 120

'''''''''|'''''''''|'''''''''|'''''''''|'''''''''|'''''''''|'''''''''|'''''''''|'''''''''|'''''''''|'''''''''|'''''''''|

ROI_00014_iTzDak19-1 SIVAQLSRPDPALAALTNDHLVALACLGGRPALDAVKKGLPHAPELIRRVNSRIGERTSHRVADLAHVVRVLGFFQSHSHPAQAFDDAMTQFGMSRHGLVQLFRRVGVTEFEARCGTIPP 120

ROI_00001_iTzDak19-2 SIVAQLSRPDPALAALTNDHLVALACLGGRPALDAVKKGLPHAPELIRRVNSRIGERTSHRVADLAHVVRVLGFFQSHSHPAQAFDDAMTQFGMSRHGLVQLFRRVGVTEFEARCGTIPP 120

ROI_00006_iTzDak19-2 SIVAQLSRPDPALAALTNDHLVALACLGGRPALDAVKKGLPHAPELIRRVNSRIGERTSHRVADLAHVVRVLGFFQSHSHPAQAFDDAMTQFGMSRHGLVQLFRRVGVTEFEARCGTIPP 120

ROI_00013_iTzDak19-3 SIVAQLSRPDPALAALTNDHLVALACLGGRPALDAVKKGLPHAPELIRRVNSRIGERTSHRVADLAHVVRVLGFFQSHSHPAQAFDDAMTQFGMSRHGLVQLFRRVGVTEFEARCGTIPP 120

ROI_00018_iTzDak19-3 SIVAQLSRPDPALAALTNDHLVALACLGGRPALDAVKKGLPHAPELIRRVNSRIGERTSHRVADLAHVVRVLGFFQSHSHPAQAFDDAMTQFGMSRHGLVQLFRRVGVTEFEARCGTIPP 120

ROI_00007_iTzLuk21-3 SIVAQLSRPDPALAALTNDHLVALACLGGRPALDAVKKGLPHAPELIRRVNSRIGERTSHRVADLAHVVRVLGFFQSHSHPAQAFDDAMTQFGMSRHGLVQLFRRVGVTEFEARCGTIPP 120

ROI_00012_iTzLuk21-3 SIVAQLSRPDPALAALTNDHLVALACLGGRPALDAVKKGLPHAPELIRRVNSRIGERTSHRVADLAHVVRVLGFFQSHSHPAQAFDDAMTQFGMSRHGLVQLFRRVGVTEFEARCGTIPP 120

ROI_00013_iTzLuk21-1 SIVAQLSRPDPALAALTNDHLVALACLGGRPALDAVKKGLPHAPELIRRVNSRIGERTSHRVADLAHVVRVLGFFQSHSHPAQAFDDAMTQFGMSRHGLVQLFRRVGVTEFEARCGTIPP 120

ROI_00018_iTzLuk21-1 SIVAQLSRPDPALAALTNDHLVALACLGGRPALDAVKKGLPHAPELIRRVNSRIGERTSHRVADLAHVVRVLGFFQSHSHPAQAFDDAMTQFGMSRHGLVQLFRRVGVTEFEARCGTIPP 120

ROI_00005_iTzLuk21-4 SIVAQLSRPDPALAALTNDHLVALACLGGRPALDAVKKGLPHAPELIRRVNSRIGERTSHRVADLAHVVRVLGFFQSHSHPAQAFDDAMTQFGMSRHGLVQLFRRVGVTEFEARCGTIPP 120

ROI_00006_iTzLuk21-4 SIVAQLSRPDPALAALTNDHLVALACLGGRPALDAVKKGLPHAPELIRRVNSRIGERTSHRVADLAHVVRVLGFFQSHSHPAQAFDDAMTQFGMSRHGLVQLFRRVGVTEFEARCGTIPP 120

ROI_00006_iTzLuk21-5 SIVAQLSRPDPALAALTNDHLVALACLGGRPALDAVKKGLPHAPELIRRVNSRIGERTSHRVADLAHVVRVLGFFQSHSHPAQAFDDAMTQFGMSRHGLVQLFRRVGVTEFEARCGTIPP 120

ROI_00011_iTzLuk21-5 SIVAQLSRPDPALAALTNDHLVALACLGGRPALDAVKKGLPHAPELIRRVNSRIGERTSHRVADLAHVVRVLGFFQSHSHPAQAFDDAMTQFGMSRHGLVQLFRRVGVTEFEARCGTIPP 120

ROI_00013_iTzLuk21-2 SIVAQLSRPDPALAALTNDHLVALACLGGRPALDAVKKGLPHAPELIRRVNSRIGERTSHRVADLAHVVRVLGFFQSHSHPAQAFDDAMTQFGMSRHGLVQLFRRVGVTEFEARCGTIPP 120

ROI_00018_iTzLuk21-2 SIVAQLSRPDPALAALTNDHLVALACLGGRPALDAVKKGLPHAPELIRRVNSRIGERTSHRVADLAHVVRVLGFFQSHSHPAQAFDDAMTQFGMSRHGLVQLFRRVGVTEFEARCGTIPP 120

Tal3a_PXO99A SIVAQLSRPDPALAALTNDHLVALACLGGRPALDAVKKGLPHAPELIRRVNSRIGERTSHRVADLAHVVRVLGFFQSHSHPAQAFDDAMTQFGMSRHGLVQLFRRVGVTEFEARCGTIPP 120

Tal3b_PXO99A SIVAQLSRRDPALAALTNDQLVALACLGGRPAPHSRKRKSHD------------------------------------------------------------------------------ 42

PthXo1_PXO99A SIVAQLSRPDPALAALTNDHLVALACLGGRPAMDAVKKGLPHAPELIRRVNRRIGERTSHRVADYAQVVRVLEFFQCHSHPAYAFDEAMTQFGMSRNGLVQLFRRVGVTELEARGGTLPP 120

Consensus SIVAQLSRXDPALAALTNDXLVALACLGGRPAXXXXKXXXXXAPELIRRVNXRIGERTSHRVADXAXVVRVLXFFQXHSHPAXAFDXAMTQFGMSRXGLVQLFRRVGVTEXEARXGTJPP 120

140 160 180 200 220 240

'''''''''|'''''''''|'''''''''|'''''''''|'''''''''|'''''''''|'''''''''|'''''''''|'''''''''|'''''''''|'''''''''|'''''''''|

ROI_00014_iTzDak19-1 ASQRWDRILQASGTKRAKPSPTSAQTPDQASLHAFPDSLERDLDAPSPMHEGDQTRASRRKRS--------------------------------------------------------- 183

ROI_00001_iTzDak19-2 ASQRWDRILQASGTKRAKPSPTSAQTPDQASLHAFPDSLERDLDAPSPMHEGDQTRASRRKRS--------------------------------------------------------- 183

ROI_00006_iTzDak19-2 ASQRWDRILQASGTKRAKPSPTSAQTPDQASLHAFPDSLERDLDAPSPMHEGDQTRASRRKRS--------------------------------------------------------- 183

ROI_00013_iTzDak19-3 ASQRWDRILQASGTKRAKPSPTSAQTPDQASLHAFPDSLERDLDAPSPMHEGDQTRASRRKRS--------------------------------------------------------- 183

ROI_00018_iTzDak19-3 ASQRWDRILQASGTKRAKPSPTSAQTPDQASLHAFPDSLERDLDAPSPMHEGDQTRASRRKRS--------------------------------------------------------- 183

ROI_00007_iTzLuk21-3 ASQRWDRILQASGTKRAKPSPTSAQTPDQASLHAFPDSLERDLDAPSPMHEGDQTRASRRKRS--------------------------------------------------------- 183

ROI_00012_iTzLuk21-3 ASQRWDRILQASGTKRAKPSPTSAQTPDQASLHAFPDSLERDLDAPSPMHEGDQTRASRRKRS--------------------------------------------------------- 183

ROI_00013_iTzLuk21-1 ASQRWDRILQASGTKRAKPSPTSAQTPDQASLHAFPDSLERDLDAPSPMHEGDQTRASRRKRS--------------------------------------------------------- 183

ROI_00018_iTzLuk21-1 ASQRWDRILQASGTKRAKPSPTSAQTPDQASLHAFPDSLERDLDAPSPMHEGDQTRASRRKRS--------------------------------------------------------- 183

ROI_00005_iTzLuk21-4 ASQRWDRILQASGTKRAKPSPTSAQTPDQASLHAFPDSLERDLDAPSPMHEGDQTRASRRKRS--------------------------------------------------------- 183

ROI_00006_iTzLuk21-4 ASQRWDRILQASGTKRAKPSPTSAQTPDQASLHAFPDSLERDLDAPSPMHEGDQTRASRRKRS--------------------------------------------------------- 183

ROI_00006_iTzLuk21-5 ASQRWDRILQASGTKRAKPSPTSAQTPDQASLHAFPDSLERDLDAPSPMHEGDQTRASRRKRS--------------------------------------------------------- 183

ROI_00011_iTzLuk21-5 ASQRWDRILQASGTKRAKPSPTSAQTPDQASLHAFPDSLERDLDAPSPMHEGDQTRASRRKRS--------------------------------------------------------- 183

ROI_00013_iTzLuk21-2 ASQRWDRILQASGTKRAKPSPTSAQTPDQASLHAFPDSLERDLDAPSPMHEGDQTRASRRKRS--------------------------------------------------------- 183

ROI_00018_iTzLuk21-2 ASQRWDRILQASGTKRAKPSPTSAQTPDQASLHAFPDSLERDLDAPSPMHEGDQTRASRRKRS--------------------------------------------------------- 183

Tal3a_PXO99A ASQRWDRILQASGTKRAKPSPTSAQTPDQASLHAFPDSLERDLDAPSPMHEGDQTRASRRKRS--------------------------------------------------------- 183

Tal3b_PXO99A ------------------------------------------------------------------------------------------------------------------------ 42

PthXo1_PXO99A ASQRWDRILQASGMKRAKPSPTSAQTPDQASLHAFADSLERDLDAPSPMHEGDQTGASSRKRSRSDRAVTGPSAQHSFEVRVPEQRDALHLPLSWRVKRPRTRIGGGLPDPGTPIAADLA 240

Consensus ASQRWDRILQASGXKRAKPSPTSAQTPDQASLHAFXDSLERDLDAPSPMHEGDQTXASXRKRSRSDRAVTGPSAQHSFEVRVPEQRDALHLPLSWRVKRPRTRIGGGLPDPGTPIAADLA 240

260 280

'''''''''|'''''''''|'''''''''|'''''''''|''''''

ROI_00014_iTzDak19-1 ---------------------------------------------- 183

ROI_00001_iTzDak19-2 ---------------------------------------------- 183

ROI_00006_iTzDak19-2 ---------------------------------------------- 183

ROI_00013_iTzDak19-3 ---------------------------------------------- 183

ROI_00018_iTzDak19-3 ---------------------------------------------- 183

ROI_00007_iTzLuk21-3 ---------------------------------------------- 183

ROI_00012_iTzLuk21-3 ---------------------------------------------- 183

ROI_00013_iTzLuk21-1 ---------------------------------------------- 183

ROI_00018_iTzLuk21-1 ---------------------------------------------- 183

ROI_00005_iTzLuk21-4 ---------------------------------------------- 183

ROI_00006_iTzLuk21-4 ---------------------------------------------- 183

ROI_00006_iTzLuk21-5 ---------------------------------------------- 183

ROI_00011_iTzLuk21-5 ---------------------------------------------- 183

ROI_00013_iTzLuk21-2 ---------------------------------------------- 183

ROI_00018_iTzLuk21-2 ---------------------------------------------- 183

Tal3a_PXO99A ---------------------------------------------- 183

Tal3b_PXO99A ---------------------------------------------- 42

PthXo1_PXO99A ASSTVMWEQDAAPFAGAADDFPAFNEEELAWLMELLPQSGSVGGTI 286

Consensus ASSTVMWEQDAAPFAGAADDFPAFNEEELAWLMELLPQSGSVGGTI 286
