# Supplementary material for: Genome editing of an African elite rice variety confers resistance against endemic and emerging Xanthomonas oryzae pv. oryzae strains
Source: eLife. 2023 Jun 20;12:e84864. doi: 10.7554/eLife.84864 (PMC10322153; doi:10.7554/eLife.84864)
Supplement: Supplementary file 1. — (a) List of Xoo strains used in this study (b) Disease survey in multiple rice growing areas in Tanzania in 2022. (c) Sequences of Xa1 and Xa4 genes from Oryza sativa cv. Komboka. (d) Characteristics of the TALome of Xanthomonas oryzae pv. oryzae Tanzanian strains. (e) OsSWEET promoter sequences for the rice varieties Komboka and Kitaake. (f) Guide RNA sequences. (g) EBE sequences in select OsSWEET promoters of Komboka wild-type and CRISPR-edited lines. (h) List of SNPs and Indels in OsSWEET promoters after editing of the rice varieties of Komboka and Kitaake (i) List of T0 lines with biallelic mutations at all targeted EBEs. (j) Screening of the T1 generation for homozygous mutations in all six SWEET EBEs. (k) List of T1 lines with homozygous mutations of all targeted EBEs. n.d. not determined. (l) List of primers used in this study (m) Alignment of OsSWEET11a promoters from the rice cv. Komboka and Kitaake. [file elife-84864-supp1.docx]

**Supplementary file 1a. List of *Xoo* strains used in this study**

| **CIX° Number** | **Strain name** | **CFBP ID** | **Country of origin** | **Region*** | **Year of isolation** | **NCBI**  **Accession #** |
| --- | --- | --- | --- | --- | --- | --- |
|  |  |  |  |  |  |  |
| 607 | Nati Park | 8172 | Benin | WA | 2013 | CP033173 |
| 609 | Tanguieta3 | NA | Benin | WA | 2013 | NA |
| 705 | Karfiguela13 | NA | Burkina Faso | WA | 2013 | NA |
| 2679 | BAI3 | 7321 | Burkina Faso | WA | 2004 | GCF_003031385.1 |
| 4127 | BAI250 | NA | Burkina Faso | WA | 2016 | NA |
| 1917 | AXO1947 | NA | Cameroon | CA | NA | GCF_001466505.1 |
| 2801 | CFBP1948 | 1948 | Cameroon | CA | 1979 | GCF_004355465.1 |
| 1042 | CII-2 | NA | Ivory Coast | WA | NA | NA |
| 4083 | CII-1 | NA | Ivory Coast | WA | NA | NA |
| 894 | MAI145 | NA | Mali | WA | 2012 | GCF_002850095.1 |
| 4079 | MAI73 | NA | Mali | WA | 2012 | GCF_002850075.1 |
| 629 | Toula20 | NA | Niger | WA | 2013 | GCF_004321555.1 |
| 2787 | NAI9 | 7324 | Niger | WA | 2004 | CP033177 |
| 4099 | NAI5 | 7323 | Niger | WA | 2004 | NA |
| 4482 | N2-4 | NA | Niger | WA | 2018 | NA |
| 4517 | MAI132 | NA | Niger | WA | 2013 | NA |
| 2374 | S62-2-22 | NA | Senegal | WA | 2014 | CP036377 |
| 2976 | S82-4-3 | NA | Senegal | WA | 2016 | NA |
| 4457 | iTzDak19-1 | NA | Tanzania | EA | 2019 | GCA_030033735.1 |
| 4458 | iTzDak19-2 | NA | Tanzania | EA | 2019 | GCA_030033715.1 |
| 4462 | iTzDak19-3 | NA | Tanzania | EA | 2019 | GCA_030033655.1 |
| 4505 | iTzLuk21-3 | NA | Tanzania | EA | 2021 | GCA_030033655.1 |
| 4506 | iTzLuk21-1 | NA | Tanzania | EA | 2021 | GCA_029204265.1 |
| 4507 | iTzLuk21-4 | NA | Tanzania | EA | 2021 | GCA_029224685.1 |
| 4508 | iTzLuk21-5 | NA | Tanzania | EA | 2021 | GCA_030056695.1 |
| 4509 | iTzLuk21-2 | NA | Tanzania | EA | 2021 | GCA_029204245.1 |
| 2839 | PXO86 | 7202 | Philippines | Asia | 1977 | GCA_0000948075.1 |
| 2831 | PXO61 | 7201 | Philippines | Asia | 1973 | CP021789.1 |
| 2840 | PXO99 | NA | Philippines | Asia | NA | GCA_000019585.2 |

°CIX, Collection IRD *Xanthomonas*. *WA, West Africa; CA, Central Africa; EA, East Africa.

**Supplementary file 1b. Disease survey in multiple rice growing areas in Tanzania in 2022.** Note that data were obtained from local farmers and breeders. *Xoo* isolation from infected leaf material, molecular validation and genotyping will be initiated after transfer to IRD.

| **FIELD NO.** | **VILLAGE** | **DISTRICT** | **REGION/ PROVINCE** | **VARIETY** | **SOWING DATE** | **STAGE* DURING SAMPLING** | **DISEASE SCALE**** | **GEOGRAPHIC LOCATION (GPS)** |
| --- | --- | --- | --- | --- | --- | --- | --- | --- |
| 1 | Lupiro | Ulanga | Morogoro | TXD 306 | March | Maturity | 3 | -8.368452, 36.674900 |
|  | Lupiro | Ulanga | Morogoro | TXD 306 | March | Maturity | 3 | -8.368452, 36.674900 |
|  | Lupiro | Ulanga | Morogoro | TXD 306 | March | Maturity | 3 | -8.368452, 36.674900 |
|  | Lupiro | Ulanga | Morogoro | TXD 306 | March | Maturity | 3 | -8.368452, 36.674900 |
|  | Lupiro | Ulanga | Morogoro | TXD 306 | March | Maturity | 3 | -8.368452, 36.674900 |
|  | Lupiro | Ulanga | Morogoro | TXD 306 | March | Maturity | 3 | -8.368452, 36.674900 |
|  | Lupiro | Ulanga | Morogoro | TXD 306 | March | Maturity | 3 | -8.368452, 36.674900 |
|  | Lupiro | Ulanga | Morogoro | TXD 306 | March | Maturity | 3 | -8.368452, 36.674900 |
|  | Lupiro | Ulanga | Morogoro | TXD 306 | March | Maturity | 3 | -8.368452, 36.674900 |
|  | Lupiro | Ulanga | Morogoro | TXD 306 | March | Maturity | 3 | -8.368452, 36.674900 |
|  | Lupiro | Ulanga | Morogoro | TXD 306 | March | Maturity | 3 | -8.368452, 36.674900 |
|  | Lupiro | Ulanga | Morogoro | TXD 306 | March | Maturity | 3 | -8.368452, 36.674900 |
|  | Lupiro | Ulanga | Morogoro | TXD 306 | March | Maturity | 3 | -8.368452, 36.674900 |
|  | Lupiro | Ulanga | Morogoro | TXD 306 | March | Maturity | 3 | -8.368452, 36.674900 |
|  | Lupiro | Ulanga | Morogoro | TXD 306 | March | Maturity | 3 | -8.368452, 36.674900 |
|  | Lupiro | Ulanga | Morogoro | TXD 306 | March | Maturity | 3 | -8.368452, 36.674900 |
|  | Lupiro | Ulanga | Morogoro | TXD 306 | March | Maturity | 3 | -8.368452, 36.674900 |
|  | Lupiro | Ulanga | Morogoro | TXD 306 | March | Maturity | 3 | -8.368452, 36.674900 |
|  | Lupiro | Ulanga | Morogoro | TXD 306 | March | Maturity | 3 | -8.368452, 36.674900 |
|  | Lupiro | Ulanga | Morogoro | TXD 306 | March | Maturity | 3 | -8.368452, 36.674900 |
|  | Lupiro | Ulanga | Morogoro | TXD 306 | March | Maturity | 3 | -8.368452, 36.674900 |
|  | Lupiro | Ulanga | Morogoro | TXD 306 | March | Maturity | 3 | -8.368452, 36.674900 |
|  | Lupiro | Ulanga | Morogoro | TXD 306 | March | Maturity | 3 | -8.368452, 36.674900 |
|  | Lupiro | Ulanga | Morogoro | TXD 306 | March | Maturity | 3 | -8.368452, 36.674900 |
|  | Lupiro | Ulanga | Morogoro | TXD 306 | March | Maturity | 3 | -8.368452, 36.674900 |
|  |  |  |  |  |  |  |  |  |
| 2 | Lupiro | Ulanga | Morogoro | Lawama | March | Maturity | 3 | -8.370881, 36.677017 |
|  | Lupiro | Ulanga | Morogoro | Lawama | March | Maturity | 3 | -8.370881, 36.677017 |
|  | Lupiro | Ulanga | Morogoro | Lawama | March | Maturity | 3 | -8.370881, 36.677017 |
|  | Lupiro | Ulanga | Morogoro | Lawama | March | Maturity | 3 | -8.370881, 36.677017 |
|  | Lupiro | Ulanga | Morogoro | Lawama | March | Maturity | 3 | -8.370881, 36.677017 |
|  | Lupiro | Ulanga | Morogoro | Lawama | March | Maturity | 3 | -8.370881, 36.677017 |
|  | Lupiro | Ulanga | Morogoro | Lawama | March | Maturity | 3 | -8.370881, 36.677017 |
|  | Lupiro | Ulanga | Morogoro | Lawama | March | Maturity | 3 | -8.370881, 36.677017 |
|  | Lupiro | Ulanga | Morogoro | Lawama | March | Maturity | 3 | -8.370881, 36.677017 |
|  | Lupiro | Ulanga | Morogoro | Lawama | March | Maturity | 3 | -8.370881, 36.677017 |
|  | Lupiro | Ulanga | Morogoro | Lawama | March | Maturity | 3 | -8.370881, 36.677017 |
|  | Lupiro | Ulanga | Morogoro | Lawama | March | Maturity | 3 | -8.370881, 36.677017 |
|  | Lupiro | Ulanga | Morogoro | Lawama | March | Maturity | 3 | -8.370881, 36.677017 |
|  | Lupiro | Ulanga | Morogoro | Lawama | March | Maturity | 3 | -8.370881, 36.677017 |
|  | Lupiro | Ulanga | Morogoro | Lawama | March | Maturity | 3 | -8.370881, 36.677017 |
|  | Lupiro | Ulanga | Morogoro | Lawama | March | Maturity | 3 | -8.370881, 36.677017 |
|  | Lupiro | Ulanga | Morogoro | Lawama | March | Maturity | 3 | -8.370881, 36.677017 |
|  | Lupiro | Ulanga | Morogoro | Lawama | March | Maturity | 3 | -8.370881, 36.677017 |
|  | Lupiro | Ulanga | Morogoro | Lawama | March | Maturity | 3 | -8.370881, 36.677017 |
|  | Lupiro | Ulanga | Morogoro | Lawama | March | Maturity | 3 | -8.370881, 36.677017 |
|  |  |  |  |  |  |  |  |  |
| 3 | Idunda | Ulanga | Morogoro | TXD 306 | March | Maturity | 3 | -8.482672, 36.692378 |
|  | Idunda | Ulanga | Morogoro | TXD 306 | March | Maturity | 3 | -8.482672, 36.692378 |
|  | Idunda | Ulanga | Morogoro | TXD 306 | March | Maturity | 3 | -8.482672, 36.692378 |
|  | Idunda | Ulanga | Morogoro | TXD 306 | March | Maturity | 3 | -8.482672, 36.692378 |
|  | Idunda | Ulanga | Morogoro | TXD 306 | March | Maturity | 3 | -8.482672, 36.692378 |
|  | Idunda | Ulanga | Morogoro | TXD 306 | March | Maturity | 3 | -8.482672, 36.692378 |
|  | Idunda | Ulanga | Morogoro | TXD 306 | March | Maturity | 3 | -8.482672, 36.692378 |
|  | Idunda | Ulanga | Morogoro | TXD 306 | March | Maturity | 3 | -8.482672, 36.692378 |
|  | Idunda | Ulanga | Morogoro | TXD 306 | March | Maturity | 3 | -8.482672, 36.692378 |
|  | Idunda | Ulanga | Morogoro | TXD 306 | March | Maturity | 3 | -8.482672, 36.692378 |
|  | Idunda | Ulanga | Morogoro | TXD 306 | March | Maturity | 3 | -8.482672, 36.692378 |
|  | Idunda | Ulanga | Morogoro | TXD 306 | March | Maturity | 3 | -8.482672, 36.692378 |
|  | Idunda | Ulanga | Morogoro | TXD 306 | March | Maturity | 3 | -8.482672, 36.692378 |
|  | Idunda | Ulanga | Morogoro | TXD 306 | March | Maturity | 3 | -8.482672, 36.692378 |
|  | Idunda | Ulanga | Morogoro | TXD 306 | March | Maturity | 3 | -8.482672, 36.692378 |
|  | Idunda | Ulanga | Morogoro | TXD 306 | March | Maturity | 3 | -8.482672, 36.692378 |
|  | Idunda | Ulanga | Morogoro | TXD 306 | March | Maturity | 3 | -8.482672, 36.692378 |
|  | Idunda | Ulanga | Morogoro | TXD 306 | March | Maturity | 3 | -8.482672, 36.692378 |
|  | Idunda | Ulanga | Morogoro | TXD 306 | March | Maturity | 3 | -8.482672, 36.692378 |
|  | Idunda | Ulanga | Morogoro | TXD 306 | March | Maturity | 3 | -8.482672, 36.692378 |
|  |  |  |  |  |  |  |  |  |
| 4 | Minepa | Ulanga | Morogoro | TXD 306 | March | Maturity | 3 | -8.252905, 36.683281 |
|  | Minepa | Ulanga | Morogoro | TXD 306 | March | Maturity | 3 | -8.252905, 36.683281 |
|  | Minepa | Ulanga | Morogoro | TXD 306 | March | Maturity | 3 | -8.252905, 36.683281 |
|  | Minepa | Ulanga | Morogoro | TXD 306 | March | Maturity | 3 | -8.252905, 36.683281 |
|  | Minepa | Ulanga | Morogoro | TXD 306 | March | Maturity | 3 | -8.252905, 36.683281 |
|  | Minepa | Ulanga | Morogoro | TXD 306 | March | Maturity | 3 | -8.252905, 36.683281 |
|  | Minepa | Ulanga | Morogoro | TXD 306 | March | Maturity | 3 | -8.252905, 36.683281 |
|  | Minepa | Ulanga | Morogoro | TXD 306 | March | Maturity | 3 | -8.252905, 36.683281 |
|  | Minepa | Ulanga | Morogoro | TXD 306 | March | Maturity | 3 | -8.252905, 36.683281 |
|  | Minepa | Ulanga | Morogoro | TXD 306 | March | Maturity | 3 | -8.252905, 36.683281 |
|  | Minepa | Ulanga | Morogoro | TXD 306 | March | Maturity | 3 | -8.252905, 36.683281 |
|  | Minepa | Ulanga | Morogoro | TXD 306 | March | Maturity | 3 | -8.252905, 36.683281 |
|  | Minepa | Ulanga | Morogoro | TXD 306 | March | Maturity | 3 | -8.252905, 36.683281 |
|  | Minepa | Ulanga | Morogoro | TXD 306 | March | Maturity | 3 | -8.252905, 36.683281 |
|  | Minepa | Ulanga | Morogoro | TXD 306 | March | Maturity | 3 | -8.252905, 36.683281 |
|  | Minepa | Ulanga | Morogoro | TXD 306 | March | Maturity | 3 | -8.252905, 36.683281 |
|  | Minepa | Ulanga | Morogoro | TXD 306 | March | Maturity | 3 | -8.252905, 36.683281 |
|  | Minepa | Ulanga | Morogoro | TXD 306 | March | Maturity | 3 | -8.252905, 36.683281 |
|  | Minepa | Ulanga | Morogoro | TXD 306 | March | Maturity | 3 | -8.252905, 36.683281 |
|  | Minepa | Ulanga | Morogoro | TXD 306 | March | Maturity | 3 | -8.252905, 36.683281 |
|  | Minepa | Ulanga | Morogoro | TXD 306 | March | Maturity | 3 | -8.252905, 36.683281 |
|  | Minepa | Ulanga | Morogoro | TXD 306 | March | Maturity | 3 | -8.252905, 36.683281 |
|  | Minepa | Ulanga | Morogoro | TXD 306 | March | Maturity | 3 | -8.252905, 36.683281 |
|  | Minepa | Ulanga | Morogoro | TXD 306 | March | Maturity | 3 | -8.252905, 36.683281 |
|  | Minepa | Ulanga | Morogoro | TXD 306 | March | Maturity | 3 | -8.252905, 36.683281 |
|  |  |  |  |  |  |  |  |  |
| 5 | Ifakara | Kilombero | Morogoro | Dunduli | February | Maturity | 3 | -8.137694, 36.684177 |
|  | Ifakara | Kilombero | Morogoro | Dunduli | February | Maturity | 3 | -8.137694, 36.684177 |
|  | Ifakara | Kilombero | Morogoro | Dunduli | February | Maturity | 3 | -8.137694, 36.684177 |
|  | Ifakara | Kilombero | Morogoro | Dunduli | February | Maturity | 3 | -8.137694, 36.684177 |
|  | Ifakara | Kilombero | Morogoro | Dunduli | February | Maturity | 3 | -8.137694, 36.684177 |
|  | Ifakara | Kilombero | Morogoro | Dunduli | February | Maturity | 3 | -8.137694, 36.684177 |
|  | Ifakara | Kilombero | Morogoro | Dunduli | February | Maturity | 3 | -8.137694, 36.684177 |
|  | Ifakara | Kilombero | Morogoro | Dunduli | February | Maturity | 3 | -8.137694, 36.684177 |
|  | Ifakara | Kilombero | Morogoro | Dunduli | February | Maturity | 3 | -8.137694, 36.684177 |
|  | Ifakara | Kilombero | Morogoro | Dunduli | February | Maturity | 3 | -8.137694, 36.684177 |
|  | Ifakara | Kilombero | Morogoro | Dunduli | February | Maturity | 3 | -8.137694, 36.684177 |
|  | Ifakara | Kilombero | Morogoro | Dunduli | February | Maturity | 3 | -8.137694, 36.684177 |
|  | Ifakara | Kilombero | Morogoro | Dunduli | February | Maturity | 3 | -8.137694, 36.684177 |
|  | Ifakara | Kilombero | Morogoro | Dunduli | February | Maturity | 3 | -8.137694, 36.684177 |
|  | Ifakara | Kilombero | Morogoro | Dunduli | February | Maturity | 3 | -8.137694, 36.684177 |
|  | Ifakara | Kilombero | Morogoro | Dunduli | February | Maturity | 3 | -8.137694, 36.684177 |
|  | Ifakara | Kilombero | Morogoro | Dunduli | February | Maturity | 3 | -8.137694, 36.684177 |
|  | Ifakara | Kilombero | Morogoro | Dunduli | February | Maturity | 3 | -8.137694, 36.684177 |
|  | Ifakara | Kilombero | Morogoro | Dunduli | February | Maturity | 3 | -8.137694, 36.684177 |
|  | Ifakara | Kilombero | Morogoro | Dunduli | February | Maturity | 3 | -8.137694, 36.684177 |
|  |  |  |  |  |  |  |  |  |
| 6 | Ifakara | Kilombero | Morogoro | EST PN. 326/44 | February | Maturity | 3 | -8.150813, 36.665661 |
|  | Ifakara | Kilombero | Morogoro | EST PN. 326/44 | February | Maturity | 3 | -8.150813, 36.665661 |
|  | Ifakara | Kilombero | Morogoro | EST PN. 326/44 | February | Maturity | 3 | -8.150813, 36.665661 |
|  | Ifakara | Kilombero | Morogoro | EST PN. 326/44 | February | Maturity | 3 | -8.150813, 36.665661 |
|  | Ifakara | Kilombero | Morogoro | EST PN. 326/44 | February | Maturity | 3 | -8.150813, 36.665661 |
|  | Ifakara | Kilombero | Morogoro | EST PN. 326/44 | February | Maturity | 3 | -8.150813, 36.665661 |
|  | Ifakara | Kilombero | Morogoro | EST PN. 326/44 | February | Maturity | 3 | -8.150813, 36.665661 |
|  | Ifakara | Kilombero | Morogoro | EST PN. 326/44 | February | Maturity | 3 | -8.150813, 36.665661 |
|  | Ifakara | Kilombero | Morogoro | EST PN. 326/44 | February | Maturity | 3 | -8.150813, 36.665661 |
|  | Ifakara | Kilombero | Morogoro | EST PN. 326/44 | February | Maturity | 3 | -8.150813, 36.665661 |
|  | Ifakara | Kilombero | Morogoro | EST PN. 326/44 | February | Maturity | 3 | -8.150813, 36.665661 |
|  | Ifakara | Kilombero | Morogoro | EST PN. 326/44 | February | Maturity | 3 | -8.150813, 36.665661 |
|  | Ifakara | Kilombero | Morogoro | EST PN. 326/44 | February | Maturity | 3 | -8.150813, 36.665661 |
|  | Ifakara | Kilombero | Morogoro | EST PN. 326/44 | February | Maturity | 3 | -8.150813, 36.665661 |
|  | Ifakara | Kilombero | Morogoro | EST PN. 326/44 | February | Maturity | 3 | -8.150813, 36.665661 |
|  | Ifakara | Kilombero | Morogoro | EST PN. 326/44 | February | Maturity | 3 | -8.150813, 36.665661 |
|  | Ifakara | Kilombero | Morogoro | EST PN. 326/44 | February | Maturity | 3 | -8.150813, 36.665661 |
|  | Ifakara | Kilombero | Morogoro | EST PN. 326/44 | February | Maturity | 3 | -8.150813, 36.665661 |
|  | Ifakara | Kilombero | Morogoro | EST PN. 326/44 | February | Maturity | 3 | -8.150813, 36.665661 |
|  | Ifakara | Kilombero | Morogoro | EST PN. 326/44 | February | Maturity | 3 | -8.150813, 36.665661 |
|  | Ifakara | Kilombero | Morogoro | EST PN. 326/44 | February | Maturity | 3 | -8.150813, 36.665661 |
|  | Ifakara | Kilombero | Morogoro | EST PN. 326/44 | February | Maturity | 3 | -8.150813, 36.665661 |
|  | Ifakara | Kilombero | Morogoro | EST PN. 326/44 | February | Maturity | 3 | -8.150813, 36.665661 |
|  | Ifakara | Kilombero | Morogoro | EST PN. 326/44 | February | Maturity | 3 | -8.150813, 36.665661 |
|  | Ifakara | Kilombero | Morogoro | EST PN. 326/44 | February | Maturity | 3 | -8.150813, 36.665661 |
|  |  |  |  |  |  |  |  |  |
| 7 | Ifakara | Kilombero | Morogoro | DSR PN. 137/31 | February | Maturity | 3 | -8.150813, 36.665661 |
|  | Ifakara | Kilombero | Morogoro | DSR PN. 137/31 | February | Maturity | 3 | -8.150813, 36.665661 |
|  | Ifakara | Kilombero | Morogoro | DSR PN. 137/31 | February | Maturity | 3 | -8.150813, 36.665661 |
|  | Ifakara | Kilombero | Morogoro | DSR PN. 137/31 | February | Maturity | 3 | -8.150813, 36.665661 |
|  | Ifakara | Kilombero | Morogoro | DSR PN. 137/31 | February | Maturity | 3 | -8.150813, 36.665661 |
|  | Ifakara | Kilombero | Morogoro | DSR PN. 137/31 | February | Maturity | 3 | -8.150813, 36.665661 |
|  | Ifakara | Kilombero | Morogoro | DSR PN. 137/31 | February | Maturity | 3 | -8.150813, 36.665661 |
|  | Ifakara | Kilombero | Morogoro | DSR PN. 137/31 | February | Maturity | 3 | -8.150813, 36.665661 |
|  | Ifakara | Kilombero | Morogoro | DSR PN. 137/31 | February | Maturity | 3 | -8.150813, 36.665661 |
|  | Ifakara | Kilombero | Morogoro | DSR PN. 137/31 | February | Maturity | 3 | -8.150813, 36.665661 |
|  | Ifakara | Kilombero | Morogoro | DSR PN. 137/31 | February | Maturity | 3 | -8.150813, 36.665661 |
|  | Ifakara | Kilombero | Morogoro | DSR PN. 137/31 | February | Maturity | 3 | -8.150813, 36.665661 |
|  | Ifakara | Kilombero | Morogoro | DSR PN. 137/31 | February | Maturity | 3 | -8.150813, 36.665661 |
|  | Ifakara | Kilombero | Morogoro | DSR PN. 137/31 | February | Maturity | 3 | -8.150813, 36.665661 |
|  | Ifakara | Kilombero | Morogoro | DSR PN. 137/31 | February | Maturity | 3 | -8.150813, 36.665661 |
|  | Ifakara | Kilombero | Morogoro | DSR PN. 137/31 | February | Maturity | 3 | -8.150813, 36.665661 |
|  | Ifakara | Kilombero | Morogoro | DSR PN. 137/31 | February | Maturity | 3 | -8.150813, 36.665661 |
|  | Ifakara | Kilombero | Morogoro | DSR PN. 137/31 | February | Maturity | 3 | -8.150813, 36.665661 |
|  | Ifakara | Kilombero | Morogoro | DSR PN. 137/31 | February | Maturity | 3 | -8.150813, 36.665661 |
|  | Ifakara | Kilombero | Morogoro | DSR PN. 137/31 | February | Maturity | 3 | -8.150813, 36.665661 |
|  | Ifakara | Kilombero | Morogoro | DSR PN. 137/31 | February | Maturity | 3 | -8.150813, 36.665661 |
|  | Ifakara | Kilombero | Morogoro | DSR PN. 137/31 | February | Maturity | 3 | -8.150813, 36.665661 |
|  | Ifakara | Kilombero | Morogoro | DSR PN. 137/31 | February | Maturity | 3 | -8.150813, 36.665661 |
|  | Ifakara | Kilombero | Morogoro | DSR PN. 137/31 | February | Maturity | 3 | -8.150813, 36.665661 |
|  | Ifakara | Kilombero | Morogoro | DSR PN. 137/31 | February | Maturity | 3 | -8.150813, 36.665661 |
|  |  |  |  |  |  |  |  |  |
| 8 | Njage | Mlimba | Morogoro | TXD 306 | March | Maturity | 2 | -8.253896, 36.181780 |
|  | Njage | Mlimba | Morogoro | TXD 306 | March | Maturity | 2 | -8.253896, 36.181780 |
|  | Njage | Mlimba | Morogoro | TXD 306 | March | Maturity | 2 | -8.253896, 36.181780 |
|  | Njage | Mlimba | Morogoro | TXD 306 | March | Maturity | 2 | -8.253896, 36.181780 |
|  | Njage | Mlimba | Morogoro | TXD 306 | March | Maturity | 2 | -8.253896, 36.181780 |
|  | Njage | Mlimba | Morogoro | TXD 306 | March | Maturity | 2 | -8.253896, 36.181780 |
|  | Njage | Mlimba | Morogoro | TXD 306 | March | Maturity | 2 | -8.253896, 36.181780 |
|  | Njage | Mlimba | Morogoro | TXD 306 | March | Maturity | 2 | -8.253896, 36.181780 |
|  | Njage | Mlimba | Morogoro | TXD 306 | March | Maturity | 2 | -8.253896, 36.181780 |
|  | Njage | Mlimba | Morogoro | TXD 306 | March | Maturity | 2 | -8.253896, 36.181780 |
|  | Njage | Mlimba | Morogoro | TXD 306 | March | Maturity | 2 | -8.253896, 36.181780 |
|  | Njage | Mlimba | Morogoro | TXD 306 | March | Maturity | 2 | -8.253896, 36.181780 |
|  | Njage | Mlimba | Morogoro | TXD 306 | March | Maturity | 2 | -8.253896, 36.181780 |
|  | Njage | Mlimba | Morogoro | TXD 306 | March | Maturity | 2 | -8.253896, 36.181780 |
|  | Njage | Mlimba | Morogoro | TXD 306 | March | Maturity | 2 | -8.253896, 36.181780 |
|  | Njage | Mlimba | Morogoro | TXD 306 | March | Maturity | 2 | -8.253896, 36.181780 |
|  | Njage | Mlimba | Morogoro | TXD 306 | March | Maturity | 2 | -8.253896, 36.181780 |
|  | Njage | Mlimba | Morogoro | TXD 306 | March | Maturity | 2 | -8.253896, 36.181780 |
|  | Njage | Mlimba | Morogoro | TXD 306 | March | Maturity | 2 | -8.253896, 36.181780 |
|  | Njage | Mlimba | Morogoro | TXD 306 | March | Maturity | 2 | -8.253896, 36.181780 |
|  | Njage | Mlimba | Morogoro | TXD 306 | March | Maturity | 2 | -8.253896, 36.181780 |
|  |  |  |  |  |  |  |  |  |
| 9 | Itongowa | Mlimba | Morogoro | TXD 306 | March | Maturity | 2 | -8.352210, 36.080054 |
|  | Itongowa | Mlimba | Morogoro | TXD 306 | March | Maturity | 2 | -8.352210, 36.080054 |
|  | Itongowa | Mlimba | Morogoro | TXD 306 | March | Maturity | 2 | -8.352210, 36.080054 |
|  | Itongowa | Mlimba | Morogoro | TXD 306 | March | Maturity | 2 | -8.352210, 36.080054 |
|  | Itongowa | Mlimba | Morogoro | TXD 306 | March | Maturity | 2 | -8.352210, 36.080054 |
|  | Itongowa | Mlimba | Morogoro | TXD 306 | March | Maturity | 2 | -8.352210, 36.080054 |
|  | Itongowa | Mlimba | Morogoro | TXD 306 | March | Maturity | 2 | -8.352210, 36.080054 |
|  | Itongowa | Mlimba | Morogoro | TXD 306 | March | Maturity | 2 | -8.352210, 36.080054 |
|  | Itongowa | Mlimba | Morogoro | TXD 306 | March | Maturity | 2 | -8.352210, 36.080054 |
|  | Itongowa | Mlimba | Morogoro | TXD 306 | March | Maturity | 2 | -8.352210, 36.080054 |
|  | Itongowa | Mlimba | Morogoro | TXD 306 | March | Maturity | 2 | -8.352210, 36.080054 |
|  | Itongowa | Mlimba | Morogoro | TXD 306 | March | Maturity | 2 | -8.352210, 36.080054 |
|  | Itongowa | Mlimba | Morogoro | TXD 306 | March | Maturity | 2 | -8.352210, 36.080054 |
|  | Itongowa | Mlimba | Morogoro | TXD 306 | March | Maturity | 2 | -8.352210, 36.080054 |
|  | Itongowa | Mlimba | Morogoro | TXD 306 | March | Maturity | 2 | -8.352210, 36.080054 |
|  | Itongowa | Mlimba | Morogoro | TXD 306 | March | Maturity | 2 | -8.352210, 36.080054 |
|  | Itongowa | Mlimba | Morogoro | TXD 306 | March | Maturity | 2 | -8.352210, 36.080054 |
|  | Itongowa | Mlimba | Morogoro | TXD 306 | March | Maturity | 2 | -8.352210, 36.080054 |
|  | Itongowa | Mlimba | Morogoro | TXD 306 | March | Maturity | 2 | -8.352210, 36.080054 |
|  | Itongowa | Mlimba | Morogoro | TXD 306 | March | Maturity | 2 | -8.352210, 36.080054 |
|  |  |  |  |  |  |  |  |  |
| 10 | Mkula | Kilombero | Morogoro | Lawama | March | Dough | 2 | -7.799772, 36.905489 |
|  | Mkula | Kilombero | Morogoro | Lawama | March | Dough | 2 | -7.799772, 36.905489 |
|  | Mkula | Kilombero | Morogoro | Lawama | March | Dough | 2 | -7.799772, 36.905489 |
|  | Mkula | Kilombero | Morogoro | Lawama | March | Dough | 2 | -7.799772, 36.905489 |
|  | Mkula | Kilombero | Morogoro | Lawama | March | Dough | 2 | -7.799772, 36.905489 |
|  | Mkula | Kilombero | Morogoro | Lawama | March | Dough | 2 | -7.799772, 36.905489 |
|  | Mkula | Kilombero | Morogoro | Lawama | March | Dough | 2 | -7.799772, 36.905489 |
|  | Mkula | Kilombero | Morogoro | Lawama | March | Dough | 2 | -7.799772, 36.905489 |
|  | Mkula | Kilombero | Morogoro | Lawama | March | Dough | 2 | -7.799772, 36.905489 |
|  | Mkula | Kilombero | Morogoro | Lawama | March | Dough | 2 | -7.799772, 36.905489 |
|  | Mkula | Kilombero | Morogoro | Lawama | March | Dough | 2 | -7.799772, 36.905489 |
|  |  |  |  |  |  |  |  |  |
| 11 | Dakawa | Mvomero | Morogoro | TXD 306 | March | Maturity | 4 | -6.420505, 37.544786 |
|  | Dakawa | Mvomero | Morogoro | TXD 306 | March | Maturity | 4 | -6.420505, 37.544786 |
|  | Dakawa | Mvomero | Morogoro | TXD 306 | March | Maturity | 4 | -6.420505, 37.544786 |
|  | Dakawa | Mvomero | Morogoro | TXD 306 | March | Maturity | 4 | -6.420505, 37.544786 |
|  | Dakawa | Mvomero | Morogoro | TXD 306 | March | Maturity | 4 | -6.420505, 37.544786 |
|  | Dakawa | Mvomero | Morogoro | TXD 306 | March | Maturity | 4 | -6.420505, 37.544786 |
|  | Dakawa | Mvomero | Morogoro | TXD 306 | March | Maturity | 4 | -6.420505, 37.544786 |
|  | Dakawa | Mvomero | Morogoro | TXD 306 | March | Maturity | 4 | -6.420505, 37.544786 |
|  | Dakawa | Mvomero | Morogoro | TXD 306 | March | Maturity | 4 | -6.420505, 37.544786 |
|  | Dakawa | Mvomero | Morogoro | TXD 306 | March | Maturity | 4 | -6.420505, 37.544786 |
|  | Dakawa | Mvomero | Morogoro | TXD 306 | March | Maturity | 4 | -6.420505, 37.544786 |
|  | Dakawa | Mvomero | Morogoro | TXD 306 | March | Maturity | 4 | -6.420505, 37.544786 |
|  | Dakawa | Mvomero | Morogoro | TXD 306 | March | Maturity | 4 | -6.420505, 37.544786 |
|  | Dakawa | Mvomero | Morogoro | TXD 306 | March | Maturity | 4 | -6.420505, 37.544786 |
|  | Dakawa | Mvomero | Morogoro | TXD 306 | March | Maturity | 4 | -6.420505, 37.544786 |
|  | Dakawa | Mvomero | Morogoro | TXD 306 | March | Maturity | 4 | -6.420505, 37.544786 |
|  | Dakawa | Mvomero | Morogoro | TXD 306 | March | Maturity | 4 | -6.420505, 37.544786 |
|  | Dakawa | Mvomero | Morogoro | TXD 306 | March | Maturity | 4 | -6.420505, 37.544786 |
|  | Dakawa | Mvomero | Morogoro | TXD 306 | March | Maturity | 4 | -6.420505, 37.544786 |
|  | Dakawa | Mvomero | Morogoro | TXD 306 | March | Maturity | 4 | -6.420505, 37.544786 |
|  | Dakawa | Mvomero | Morogoro | TXD 306 | March | Maturity | 4 | -6.420505, 37.544786 |
|  | Dakawa | Mvomero | Morogoro | TXD 306 | March | Maturity | 4 | -6.420505, 37.544786 |
|  | Dakawa | Mvomero | Morogoro | TXD 306 | March | Maturity | 4 | -6.420505, 37.544786 |
|  | Dakawa | Mvomero | Morogoro | TXD 306 | March | Maturity | 4 | -6.420505, 37.544786 |
|  | Dakawa | Mvomero | Morogoro | TXD 306 | March | Maturity | 4 | -6.420505, 37.544786 |
|  | Dakawa | Mvomero | Morogoro | TXD 306 | March | Maturity | 4 | -6.420505, 37.544786 |
|  |  |  |  |  |  |  |  |  |
| 12 | Dakawa | Mvomero | Morogoro | Supa | February | Milk | 4 | -6.419846, 37.544267 |
|  | Dakawa | Mvomero | Morogoro | Supa | February | Milk | 4 | -6.419846, 37.544267 |
|  | Dakawa | Mvomero | Morogoro | Supa | February | Milk | 4 | -6.419846, 37.544267 |
|  | Dakawa | Mvomero | Morogoro | Supa | February | Milk | 4 | -6.419846, 37.544267 |
|  | Dakawa | Mvomero | Morogoro | Supa | February | Milk | 4 | -6.419846, 37.544267 |
|  | Dakawa | Mvomero | Morogoro | Supa | February | Milk | 4 | -6.419846, 37.544267 |
|  | Dakawa | Mvomero | Morogoro | Supa | February | Milk | 4 | -6.419846, 37.544267 |
|  | Dakawa | Mvomero | Morogoro | Supa | February | Milk | 4 | -6.419846, 37.544267 |
|  | Dakawa | Mvomero | Morogoro | Supa | February | Milk | 4 | -6.419846, 37.544267 |
|  | Dakawa | Mvomero | Morogoro | Supa | February | Milk | 4 | -6.419846, 37.544267 |
|  | Dakawa | Mvomero | Morogoro | Supa | February | Milk | 4 | -6.419846, 37.544267 |
|  | Dakawa | Mvomero | Morogoro | Supa | February | Milk | 4 | -6.419846, 37.544267 |
|  | Dakawa | Mvomero | Morogoro | Supa | February | Milk | 4 | -6.419846, 37.544267 |
|  | Dakawa | Mvomero | Morogoro | Supa | February | Milk | 4 | -6.419846, 37.544267 |
|  | Dakawa | Mvomero | Morogoro | Supa | February | Milk | 4 | -6.419846, 37.544267 |
|  | Dakawa | Mvomero | Morogoro | Supa | February | Milk | 4 | -6.419846, 37.544267 |
|  | Dakawa | Mvomero | Morogoro | Supa | February | Milk | 4 | -6.419846, 37.544267 |
|  | Dakawa | Mvomero | Morogoro | Supa | February | Milk | 4 | -6.419846, 37.544267 |
|  | Dakawa | Mvomero | Morogoro | Supa | February | Milk | 4 | -6.419846, 37.544267 |
|  | Dakawa | Mvomero | Morogoro | Supa | February | Milk | 4 | -6.419846, 37.544267 |
|  | Dakawa | Mvomero | Morogoro | Supa | February | Milk | 4 | -6.419846, 37.544267 |
|  | Dakawa | Mvomero | Morogoro | Supa | February | Milk | 4 | -6.419846, 37.544267 |
|  | Dakawa | Mvomero | Morogoro | Supa | February | Milk | 4 | -6.419846, 37.544267 |
|  |  |  |  |  |  |  |  |  |
| 13 | Dakawa | Mvomero | Morogoro | TXD 306 | March | Maturity | 4 | -6.415932, 37.551897 |
|  | Dakawa | Mvomero | Morogoro | TXD 306 | March | Maturity | 4 | -6.415932, 37.551897 |
|  | Dakawa | Mvomero | Morogoro | TXD 306 | March | Maturity | 4 | -6.415932, 37.551897 |
|  | Dakawa | Mvomero | Morogoro | TXD 306 | March | Maturity | 4 | -6.415932, 37.551897 |
|  | Dakawa | Mvomero | Morogoro | TXD 306 | March | Maturity | 4 | -6.415932, 37.551897 |
|  | Dakawa | Mvomero | Morogoro | TXD 306 | March | Maturity | 4 | -6.415932, 37.551897 |
|  | Dakawa | Mvomero | Morogoro | TXD 306 | March | Maturity | 4 | -6.415932, 37.551897 |
|  | Dakawa | Mvomero | Morogoro | TXD 306 | March | Maturity | 4 | -6.415932, 37.551897 |
|  | Dakawa | Mvomero | Morogoro | TXD 306 | March | Maturity | 4 | -6.415932, 37.551897 |
|  | Dakawa | Mvomero | Morogoro | TXD 306 | March | Maturity | 4 | -6.415932, 37.551897 |
|  | Dakawa | Mvomero | Morogoro | TXD 306 | March | Maturity | 4 | -6.415932, 37.551897 |
|  | Dakawa | Mvomero | Morogoro | TXD 306 | March | Maturity | 4 | -6.415932, 37.551897 |
|  | Dakawa | Mvomero | Morogoro | TXD 306 | March | Maturity | 4 | -6.415932, 37.551897 |
|  | Dakawa | Mvomero | Morogoro | TXD 306 | March | Maturity | 4 | -6.415932, 37.551897 |
|  | Dakawa | Mvomero | Morogoro | TXD 306 | March | Maturity | 4 | -6.415932, 37.551897 |
|  | Dakawa | Mvomero | Morogoro | TXD 306 | March | Maturity | 4 | -6.415932, 37.551897 |
|  | Dakawa | Mvomero | Morogoro | TXD 306 | March | Maturity | 4 | -6.415932, 37.551897 |
|  | Dakawa | Mvomero | Morogoro | TXD 306 | March | Maturity | 4 | -6.415932, 37.551897 |
|  | Dakawa | Mvomero | Morogoro | TXD 306 | March | Maturity | 4 | -6.415932, 37.551897 |
|  | Dakawa | Mvomero | Morogoro | TXD 306 | March | Maturity | 4 | -6.415932, 37.551897 |
|  | Dakawa | Mvomero | Morogoro | TXD 306 | March | Maturity | 4 | -6.415932, 37.551897 |
|  | Dakawa | Mvomero | Morogoro | TXD 306 | March | Maturity | 4 | -6.415932, 37.551897 |
|  | Dakawa | Mvomero | Morogoro | TXD 306 | March | Maturity | 4 | -6.415932, 37.551897 |
|  | Dakawa | Mvomero | Morogoro | TXD 306 | March | Maturity | 4 | -6.415932, 37.551897 |
|  | Dakawa | Mvomero | Morogoro | TXD 306 | March | Maturity | 4 | -6.415932, 37.551897 |
|  |  |  |  |  |  |  |  |  |
| 14 | Dakawa | Mvomero | Morogoro | TXD 306 | March | Maturity | 3 | -6.400923, 37.567535 |
|  | Dakawa | Mvomero | Morogoro | TXD 306 | March | Maturity | 3 | -6.400923, 37.567535 |
|  | Dakawa | Mvomero | Morogoro | TXD 306 | March | Maturity | 3 | -6.400923, 37.567535 |
|  | Dakawa | Mvomero | Morogoro | TXD 306 | March | Maturity | 3 | -6.400923, 37.567535 |
|  | Dakawa | Mvomero | Morogoro | TXD 306 | March | Maturity | 3 | -6.400923, 37.567535 |
|  | Dakawa | Mvomero | Morogoro | TXD 306 | March | Maturity | 3 | -6.400923, 37.567535 |
|  | Dakawa | Mvomero | Morogoro | TXD 306 | March | Maturity | 3 | -6.400923, 37.567535 |
|  | Dakawa | Mvomero | Morogoro | TXD 306 | March | Maturity | 3 | -6.400923, 37.567535 |
|  | Dakawa | Mvomero | Morogoro | TXD 306 | March | Maturity | 3 | -6.400923, 37.567535 |
|  | Dakawa | Mvomero | Morogoro | TXD 306 | March | Maturity | 3 | -6.400923, 37.567535 |
|  | Dakawa | Mvomero | Morogoro | TXD 306 | March | Maturity | 3 | -6.400923, 37.567535 |
|  | Dakawa | Mvomero | Morogoro | TXD 306 | March | Maturity | 3 | -6.400923, 37.567535 |
|  | Dakawa | Mvomero | Morogoro | TXD 306 | March | Maturity | 3 | -6.400923, 37.567535 |
|  | Dakawa | Mvomero | Morogoro | TXD 306 | March | Maturity | 3 | -6.400923, 37.567535 |
|  | Dakawa | Mvomero | Morogoro | TXD 306 | March | Maturity | 3 | -6.400923, 37.567535 |
|  | Dakawa | Mvomero | Morogoro | TXD 306 | March | Maturity | 3 | -6.400923, 37.567535 |
|  | Dakawa | Mvomero | Morogoro | TXD 306 | March | Maturity | 3 | -6.400923, 37.567535 |
|  | Dakawa | Mvomero | Morogoro | TXD 306 | March | Maturity | 3 | -6.400923, 37.567535 |
|  | Dakawa | Mvomero | Morogoro | TXD 306 | March | Maturity | 3 | -6.400923, 37.567535 |
|  | Dakawa | Mvomero | Morogoro | TXD 306 | March | Maturity | 3 | -6.400923, 37.567535 |
|  | Dakawa | Mvomero | Morogoro | TXD 306 | March | Maturity | 3 | -6.400923, 37.567535 |
|  | Dakawa | Mvomero | Morogoro | TXD 306 | March | Maturity | 3 | -6.400923, 37.567535 |
|  | Dakawa | Mvomero | Morogoro | TXD 306 | March | Maturity | 3 | -6.400923, 37.567535 |
|  |  |  |  |  |  |  |  |  |
| 15 | Dakawa | Mvomero | Morogoro | TXD 306 | February | Maturity | 3 | -6.395002, 37.567265 |
|  | Dakawa | Mvomero | Morogoro | TXD 306 | February | Maturity | 3 | -6.395002, 37.567265 |
|  | Dakawa | Mvomero | Morogoro | TXD 306 | February | Maturity | 3 | -6.395002, 37.567265 |
|  | Dakawa | Mvomero | Morogoro | TXD 306 | February | Maturity | 3 | -6.395002, 37.567265 |
|  | Dakawa | Mvomero | Morogoro | TXD 306 | February | Maturity | 3 | -6.395002, 37.567265 |
|  | Dakawa | Mvomero | Morogoro | TXD 306 | February | Maturity | 3 | -6.395002, 37.567265 |
|  | Dakawa | Mvomero | Morogoro | TXD 306 | February | Maturity | 3 | -6.395002, 37.567265 |
|  | Dakawa | Mvomero | Morogoro | TXD 306 | February | Maturity | 3 | -6.395002, 37.567265 |
|  | Dakawa | Mvomero | Morogoro | TXD 306 | February | Maturity | 3 | -6.395002, 37.567265 |
|  | Dakawa | Mvomero | Morogoro | TXD 306 | February | Maturity | 3 | -6.395002, 37.567265 |
|  | Dakawa | Mvomero | Morogoro | TXD 306 | February | Maturity | 3 | -6.395002, 37.567265 |
|  | Dakawa | Mvomero | Morogoro | TXD 306 | February | Maturity | 3 | -6.395002, 37.567265 |
|  | Dakawa | Mvomero | Morogoro | TXD 306 | February | Maturity | 3 | -6.395002, 37.567265 |
|  | Dakawa | Mvomero | Morogoro | TXD 306 | February | Maturity | 3 | -6.395002, 37.567265 |
|  | Dakawa | Mvomero | Morogoro | TXD 306 | February | Maturity | 3 | -6.395002, 37.567265 |
|  | Dakawa | Mvomero | Morogoro | TXD 306 | February | Maturity | 3 | -6.395002, 37.567265 |
|  | Dakawa | Mvomero | Morogoro | TXD 306 | February | Maturity | 3 | -6.395002, 37.567265 |
|  | Dakawa | Mvomero | Morogoro | TXD 306 | February | Maturity | 3 | -6.395002, 37.567265 |
|  | Dakawa | Mvomero | Morogoro | TXD 306 | February | Maturity | 3 | -6.395002, 37.567265 |
|  | Dakawa | Mvomero | Morogoro | TXD 306 | February | Maturity | 3 | -6.395002, 37.567265 |
|  |  |  |  |  |  |  |  |  |
| 16 | Dakawa | Mvomero | Morogoro | TXD 306 | March | Flowering | 4 | -6.392945, 37.60610 |
|  | Dakawa | Mvomero | Morogoro | TXD 306 | March | Flowering | 4 | -6.392945, 37.60610 |
|  | Dakawa | Mvomero | Morogoro | TXD 306 | March | Flowering | 4 | -6.392945, 37.60610 |
|  | Dakawa | Mvomero | Morogoro | TXD 306 | March | Flowering | 4 | -6.392945, 37.60610 |
|  | Dakawa | Mvomero | Morogoro | TXD 306 | March | Flowering | 4 | -6.392945, 37.60610 |
|  | Dakawa | Mvomero | Morogoro | TXD 306 | March | Flowering | 4 | -6.392945, 37.60610 |
|  | Dakawa | Mvomero | Morogoro | TXD 306 | March | Flowering | 4 | -6.392945, 37.60610 |
|  | Dakawa | Mvomero | Morogoro | TXD 306 | March | Flowering | 4 | -6.392945, 37.60610 |
|  | Dakawa | Mvomero | Morogoro | TXD 306 | March | Flowering | 4 | -6.392945, 37.60610 |
|  | Dakawa | Mvomero | Morogoro | TXD 306 | March | Flowering | 4 | -6.392945, 37.60610 |
|  | Dakawa | Mvomero | Morogoro | TXD 306 | March | Flowering | 4 | -6.392945, 37.60610 |
|  | Dakawa | Mvomero | Morogoro | TXD 306 | March | Flowering | 4 | -6.392945, 37.60610 |
|  | Dakawa | Mvomero | Morogoro | TXD 306 | March | Flowering | 4 | -6.392945, 37.60610 |
|  | Dakawa | Mvomero | Morogoro | TXD 306 | March | Flowering | 4 | -6.392945, 37.60610 |
|  | Dakawa | Mvomero | Morogoro | TXD 306 | March | Flowering | 4 | -6.392945, 37.60610 |
|  | Dakawa | Mvomero | Morogoro | TXD 306 | March | Flowering | 4 | -6.392945, 37.60610 |
|  | Dakawa | Mvomero | Morogoro | TXD 306 | March | Flowering | 4 | -6.392945, 37.60610 |
|  | Dakawa | Mvomero | Morogoro | TXD 306 | March | Flowering | 4 | -6.392945, 37.60610 |
|  | Dakawa | Mvomero | Morogoro | TXD 306 | March | Flowering | 4 | -6.392945, 37.60610 |
|  | Dakawa | Mvomero | Morogoro | TXD 306 | March | Flowering | 4 | -6.392945, 37.60610 |
|  | Dakawa | Mvomero | Morogoro | TXD 306 | March | Flowering | 4 | -6.392945, 37.60610 |
|  |  |  |  |  |  |  |  |  |
| 17 | Dakawa | Mvomero | Morogoro | TXD 306 | March | Milk | 5 | -6.395147, 37.574687 |
|  | Dakawa | Mvomero | Morogoro | TXD 306 | March | Milk | 5 | -6.395147, 37.574687 |
|  | Dakawa | Mvomero | Morogoro | TXD 306 | March | Milk | 5 | -6.395147, 37.574687 |
|  | Dakawa | Mvomero | Morogoro | TXD 306 | March | Milk | 5 | -6.395147, 37.574687 |
|  | Dakawa | Mvomero | Morogoro | TXD 306 | March | Milk | 5 | -6.395147, 37.574687 |
|  | Dakawa | Mvomero | Morogoro | TXD 306 | March | Milk | 5 | -6.395147, 37.574687 |
|  | Dakawa | Mvomero | Morogoro | TXD 306 | March | Milk | 5 | -6.395147, 37.574687 |
|  | Dakawa | Mvomero | Morogoro | TXD 306 | March | Milk | 5 | -6.395147, 37.574687 |
|  | Dakawa | Mvomero | Morogoro | TXD 306 | March | Milk | 5 | -6.395147, 37.574687 |
|  | Dakawa | Mvomero | Morogoro | TXD 306 | March | Milk | 5 | -6.395147, 37.574687 |
|  | Dakawa | Mvomero | Morogoro | TXD 306 | March | Milk | 5 | -6.395147, 37.574687 |
|  | Dakawa | Mvomero | Morogoro | TXD 306 | March | Milk | 5 | -6.395147, 37.574687 |
|  | Dakawa | Mvomero | Morogoro | TXD 306 | March | Milk | 5 | -6.395147, 37.574687 |
|  | Dakawa | Mvomero | Morogoro | TXD 306 | March | Milk | 5 | -6.395147, 37.574687 |
|  | Dakawa | Mvomero | Morogoro | TXD 306 | March | Milk | 5 | -6.395147, 37.574687 |
|  | Dakawa | Mvomero | Morogoro | TXD 306 | March | Milk | 5 | -6.395147, 37.574687 |
|  | Dakawa | Mvomero | Morogoro | TXD 306 | March | Milk | 5 | -6.395147, 37.574687 |
|  | Dakawa | Mvomero | Morogoro | TXD 306 | March | Milk | 5 | -6.395147, 37.574687 |
|  | Dakawa | Mvomero | Morogoro | TXD 306 | March | Milk | 5 | -6.395147, 37.574687 |
|  | Dakawa | Mvomero | Morogoro | TXD 306 | March | Milk | 5 | -6.395147, 37.574687 |
|  | Dakawa | Mvomero | Morogoro | TXD 306 | March | Milk | 5 | -6.395147, 37.574687 |
|  | Dakawa | Mvomero | Morogoro | TXD 306 | March | Milk | 5 | -6.395147, 37.574687 |
|  |  |  |  |  |  |  |  |  |
| 18 | Dakawa | Mvomero | Morogoro | TXD 306 | February | Maturity | 4 | -6.385051, 37.598029 |
|  | Dakawa | Mvomero | Morogoro | TXD 306 | February | Maturity | 4 | -6.385051, 37.598029 |
|  | Dakawa | Mvomero | Morogoro | TXD 306 | February | Maturity | 4 | -6.385051, 37.598029 |
|  | Dakawa | Mvomero | Morogoro | TXD 306 | February | Maturity | 4 | -6.385051, 37.598029 |
|  | Dakawa | Mvomero | Morogoro | TXD 306 | February | Maturity | 4 | -6.385051, 37.598029 |
|  | Dakawa | Mvomero | Morogoro | TXD 306 | February | Maturity | 4 | -6.385051, 37.598029 |
|  | Dakawa | Mvomero | Morogoro | TXD 306 | February | Maturity | 4 | -6.385051, 37.598029 |
|  | Dakawa | Mvomero | Morogoro | TXD 306 | February | Maturity | 4 | -6.385051, 37.598029 |
|  | Dakawa | Mvomero | Morogoro | TXD 306 | February | Maturity | 4 | -6.385051, 37.598029 |
|  | Dakawa | Mvomero | Morogoro | TXD 306 | February | Maturity | 4 | -6.385051, 37.598029 |
|  | Dakawa | Mvomero | Morogoro | TXD 306 | February | Maturity | 4 | -6.385051, 37.598029 |
|  | Dakawa | Mvomero | Morogoro | TXD 306 | February | Maturity | 4 | -6.385051, 37.598029 |
|  | Dakawa | Mvomero | Morogoro | TXD 306 | February | Maturity | 4 | -6.385051, 37.598029 |
|  | Dakawa | Mvomero | Morogoro | TXD 306 | February | Maturity | 4 | -6.385051, 37.598029 |
|  | Dakawa | Mvomero | Morogoro | TXD 306 | February | Maturity | 4 | -6.385051, 37.598029 |
|  |  |  |  |  |  |  |  |  |
| 19 | Lukenge | Mvomero | Morogoro | TXD 306 | March | Maturity | 4 | -6.234562, 37.678811 |
|  | Lukenge | Mvomero | Morogoro | TXD 306 | March | Maturity | 4 | -6.234562, 37.678811 |
|  | Lukenge | Mvomero | Morogoro | TXD 306 | March | Maturity | 4 | -6.234562, 37.678811 |
|  | Lukenge | Mvomero | Morogoro | TXD 306 | March | Maturity | 4 | -6.234562, 37.678811 |
|  | Lukenge | Mvomero | Morogoro | TXD 306 | March | Maturity | 4 | -6.234562, 37.678811 |
|  | Lukenge | Mvomero | Morogoro | TXD 306 | March | Maturity | 4 | -6.234562, 37.678811 |
|  | Lukenge | Mvomero | Morogoro | TXD 306 | March | Maturity | 4 | -6.234562, 37.678811 |
|  | Lukenge | Mvomero | Morogoro | TXD 306 | March | Maturity | 4 | -6.234562, 37.678811 |
|  | Lukenge | Mvomero | Morogoro | TXD 306 | March | Maturity | 4 | -6.234562, 37.678811 |
|  | Lukenge | Mvomero | Morogoro | TXD 306 | March | Maturity | 4 | -6.234562, 37.678811 |
|  | Lukenge | Mvomero | Morogoro | TXD 306 | March | Maturity | 4 | -6.234562, 37.678811 |
|  | Lukenge | Mvomero | Morogoro | TXD 306 | March | Maturity | 4 | -6.234562, 37.678811 |
|  | Lukenge | Mvomero | Morogoro | TXD 306 | March | Maturity | 4 | -6.234562, 37.678811 |
|  | Lukenge | Mvomero | Morogoro | TXD 306 | March | Maturity | 4 | -6.234562, 37.678811 |
|  | Lukenge | Mvomero | Morogoro | TXD 306 | March | Maturity | 4 | -6.234562, 37.678811 |
|  | Lukenge | Mvomero | Morogoro | TXD 306 | March | Maturity | 4 | -6.234562, 37.678811 |
|  | Lukenge | Mvomero | Morogoro | TXD 306 | March | Maturity | 4 | -6.234562, 37.678811 |
|  | Lukenge | Mvomero | Morogoro | TXD 306 | March | Maturity | 4 | -6.234562, 37.678811 |
|  | Lukenge | Mvomero | Morogoro | TXD 306 | March | Maturity | 4 | -6.234562, 37.678811 |
|  | Lukenge | Mvomero | Morogoro | TXD 306 | March | Maturity | 4 | -6.234562, 37.678811 |
|  | Lukenge | Mvomero | Morogoro | TXD 306 | March | Maturity | 4 | -6.234562, 37.678811 |
|  | Lukenge | Mvomero | Morogoro | TXD 306 | March | Maturity | 4 | -6.234562, 37.678811 |
|  | Lukenge | Mvomero | Morogoro | TXD 306 | March | Maturity | 4 | -6.234562, 37.678811 |
|  | Lukenge | Mvomero | Morogoro | TXD 306 | March | Maturity | 4 | -6.234562, 37.678811 |
|  | Lukenge | Mvomero | Morogoro | TXD 306 | March | Maturity | 4 | -6.234562, 37.678811 |
|  | Lukenge | Mvomero | Morogoro | TXD 306 | March | Maturity | 4 | -6.234562, 37.678811 |
|  |  |  |  |  |  |  |  |  |
| 20 | Lukenge | Mvomero | Morogoro | TXD 306 | April | Milk | 3 | -6.238532, 37.679524 |
|  | Lukenge | Mvomero | Morogoro | TXD 306 | April | Milk | 3 | -6.238532, 37.679524 |
|  | Lukenge | Mvomero | Morogoro | TXD 306 | April | Milk | 3 | -6.238532, 37.679524 |
|  | Lukenge | Mvomero | Morogoro | TXD 306 | April | Milk | 3 | -6.238532, 37.679524 |
|  | Lukenge | Mvomero | Morogoro | TXD 306 | April | Milk | 3 | -6.238532, 37.679524 |
|  | Lukenge | Mvomero | Morogoro | TXD 306 | April | Milk | 3 | -6.238532, 37.679524 |
|  | Lukenge | Mvomero | Morogoro | TXD 306 | April | Milk | 3 | -6.238532, 37.679524 |
|  | Lukenge | Mvomero | Morogoro | TXD 306 | April | Milk | 3 | -6.238532, 37.679524 |
|  | Lukenge | Mvomero | Morogoro | TXD 306 | April | Milk | 3 | -6.238532, 37.679524 |
|  | Lukenge | Mvomero | Morogoro | TXD 306 | April | Milk | 3 | -6.238532, 37.679524 |
|  | Lukenge | Mvomero | Morogoro | TXD 306 | April | Milk | 3 | -6.238532, 37.679524 |
|  | Lukenge | Mvomero | Morogoro | TXD 306 | April | Milk | 3 | -6.238532, 37.679524 |
|  | Lukenge | Mvomero | Morogoro | TXD 306 | April | Milk | 3 | -6.238532, 37.679524 |
|  | Lukenge | Mvomero | Morogoro | TXD 306 | April | Milk | 3 | -6.238532, 37.679524 |
|  | Lukenge | Mvomero | Morogoro | TXD 306 | April | Milk | 3 | -6.238532, 37.679524 |
|  | Lukenge | Mvomero | Morogoro | TXD 306 | April | Milk | 3 | -6.238532, 37.679524 |
|  | Lukenge | Mvomero | Morogoro | TXD 306 | April | Milk | 3 | -6.238532, 37.679524 |
|  | Lukenge | Mvomero | Morogoro | TXD 306 | April | Milk | 3 | -6.238532, 37.679524 |
|  | Lukenge | Mvomero | Morogoro | TXD 306 | April | Milk | 3 | -6.238532, 37.679524 |
|  | Lukenge | Mvomero | Morogoro | TXD 306 | April | Milk | 3 | -6.238532, 37.679524 |
|  | Lukenge | Mvomero | Morogoro | TXD 306 | April | Milk | 3 | -6.238532, 37.679524 |
|  | Lukenge | Mvomero | Morogoro | TXD 306 | April | Milk | 3 | -6.238532, 37.679524 |
|  | Lukenge | Mvomero | Morogoro | TXD 306 | April | Milk | 3 | -6.238532, 37.679524 |
|  | Lukenge | Mvomero | Morogoro | TXD 306 | April | Milk | 3 | -6.238532, 37.679524 |
|  | Lukenge | Mvomero | Morogoro | TXD 306 | April | Milk | 3 | -6.238532, 37.679524 |
|  |  |  |  |  |  |  |  |  |
| 21 | Kwam-tonga | Mvomero | Morogoro | TXD 306 | March | Maturity | 2 | -6.189823, 37.599676 |
|  | Kwam-tonga | Mvomero | Morogoro | TXD 306 | March | Maturity | 2 | -6.189823, 37.599676 |
|  | Kwam-tonga | Mvomero | Morogoro | TXD 306 | March | Maturity | 2 | -6.189823, 37.599676 |
|  | Kwam-tonga | Mvomero | Morogoro | TXD 306 | March | Maturity | 2 | -6.189823, 37.599676 |
|  | Kwam-tonga | Mvomero | Morogoro | TXD 306 | March | Maturity | 2 | -6.189823, 37.599676 |
|  | Kwam-tonga | Mvomero | Morogoro | TXD 306 | March | Maturity | 2 | -6.189823, 37.599676 |
|  | Kwam-tonga | Mvomero | Morogoro | TXD 306 | March | Maturity | 2 | -6.189823, 37.599676 |
|  | Kwam-tonga | Mvomero | Morogoro | TXD 306 | March | Maturity | 2 | -6.189823, 37.599676 |
|  | Kwam-tonga | Mvomero | Morogoro | TXD 306 | March | Maturity | 2 | -6.189823, 37.599676 |
|  | Kwam-tonga | Mvomero | Morogoro | TXD 306 | March | Maturity | 2 | -6.189823, 37.599676 |
|  | Kwam-tonga | Mvomero | Morogoro | TXD 306 | March | Maturity | 2 | -6.189823, 37.599676 |
|  | Kwam-tonga | Mvomero | Morogoro | TXD 306 | March | Maturity | 2 | -6.189823, 37.599676 |
|  | Kwam-tonga | Mvomero | Morogoro | TXD 306 | March | Maturity | 2 | -6.189823, 37.599676 |
|  | Kwam-tonga | Mvomero | Morogoro | TXD 306 | March | Maturity | 2 | -6.189823, 37.599676 |
|  | Kwam-tonga | Mvomero | Morogoro | TXD 306 | March | Maturity | 2 | -6.189823, 37.599676 |
|  | Kwam-tonga | Mvomero | Morogoro | TXD 306 | March | Maturity | 2 | -6.189823, 37.599676 |
|  | Kwam-tonga | Mvomero | Morogoro | TXD 306 | March | Maturity | 2 | -6.189823, 37.599676 |
|  | Kwam-tonga | Mvomero | Morogoro | TXD 306 | March | Maturity | 2 | -6.189823, 37.599676 |
|  |  |  |  |  |  |  |  |  |
| 22 | Mkindo | Mvomero | Morogoro | TXD 306 | March | Maturity | 2 | -6.256603, 37.539718 |
|  | Mkindo | Mvomero | Morogoro | TXD 306 | March | Maturity | 2 | -6.256603, 37.539718 |
|  | Mkindo | Mvomero | Morogoro | TXD 306 | March | Maturity | 2 | -6.256603, 37.539718 |
|  | Mkindo | Mvomero | Morogoro | TXD 306 | March | Maturity | 2 | -6.256603, 37.539718 |
|  | Mkindo | Mvomero | Morogoro | TXD 306 | March | Maturity | 2 | -6.256603, 37.539718 |
|  | Mkindo | Mvomero | Morogoro | TXD 306 | March | Maturity | 2 | -6.256603, 37.539718 |
|  | Mkindo | Mvomero | Morogoro | TXD 306 | March | Maturity | 2 | -6.256603, 37.539718 |
|  | Mkindo | Mvomero | Morogoro | TXD 306 | March | Maturity | 2 | -6.256603, 37.539718 |
|  | Mkindo | Mvomero | Morogoro | TXD 306 | March | Maturity | 2 | -6.256603, 37.539718 |
|  | Mkindo | Mvomero | Morogoro | TXD 306 | March | Maturity | 2 | -6.256603, 37.539718 |
|  | Mkindo | Mvomero | Morogoro | TXD 306 | March | Maturity | 2 | -6.256603, 37.539718 |
|  | Mkindo | Mvomero | Morogoro | TXD 306 | March | Maturity | 2 | -6.256603, 37.539718 |
|  | Mkindo | Mvomero | Morogoro | TXD 306 | March | Maturity | 2 | -6.256603, 37.539718 |
|  | Mkindo | Mvomero | Morogoro | TXD 306 | March | Maturity | 2 | -6.256603, 37.539718 |
|  | Mkindo | Mvomero | Morogoro | TXD 306 | March | Maturity | 2 | -6.256603, 37.539718 |
|  | Mkindo | Mvomero | Morogoro | TXD 306 | March | Maturity | 2 | -6.256603, 37.539718 |
|  | Mkindo | Mvomero | Morogoro | TXD 306 | March | Maturity | 2 | -6.256603, 37.539718 |
|  | Mkindo | Mvomero | Morogoro | TXD 306 | March | Maturity | 2 | -6.256603, 37.539718 |
|  |  |  |  |  |  |  |  |  |
| 23 | Ruvu (Chauru) | Baga-moyo | Coast | TXD 306 | March | Maturity | 3 | -6.742699, 38.668300 |
|  | Ruvu (Chauru) | Baga-moyo | Coast | TXD 306 | March | Maturity | 3 | -6.742699, 38.668300 |
|  | Ruvu (Chauru) | Baga-moyo | Coast | TXD 306 | March | Maturity | 3 | -6.742699, 38.668300 |
|  | Ruvu (Chauru) | Baga-moyo | Coast | TXD 306 | March | Maturity | 3 | -6.742699, 38.668300 |
|  | Ruvu (Chauru) | Baga-moyo | Coast | TXD 306 | March | Maturity | 3 | -6.742699, 38.668300 |
|  | Ruvu (Chauru) | Baga-moyo | Coast | TXD 306 | March | Maturity | 3 | -6.742699, 38.668300 |
|  | Ruvu (Chauru) | Baga-moyo | Coast | TXD 306 | March | Maturity | 3 | -6.742699, 38.668300 |
|  | Ruvu (Chauru) | Baga-moyo | Coast | TXD 306 | March | Maturity | 3 | -6.742699, 38.668300 |
|  | Ruvu (Chauru) | Baga-moyo | Coast | TXD 306 | March | Maturity | 3 | -6.742699, 38.668300 |
|  | Ruvu (Chauru) | Baga-moyo | Coast | TXD 306 | March | Maturity | 3 | -6.742699, 38.668300 |
|  | Ruvu (Chauru) | Baga-moyo | Coast | TXD 306 | March | Maturity | 3 | -6.742699, 38.668300 |
|  | Ruvu (Chauru) | Baga-moyo | Coast | TXD 306 | March | Maturity | 3 | -6.742699, 38.668300 |
|  | Ruvu (Chauru) | Baga-moyo | Coast | TXD 306 | March | Maturity | 3 | -6.742699, 38.668300 |
|  | Ruvu (Chauru) | Baga-moyo | Coast | TXD 306 | March | Maturity | 3 | -6.742699, 38.668300 |
|  | Ruvu (Chauru) | Baga-moyo | Coast | TXD 306 | March | Maturity | 3 | -6.742699, 38.668300 |
|  | Ruvu (Chauru) | Baga-moyo | Coast | TXD 306 | March | Maturity | 3 | -6.742699, 38.668300 |
|  | Ruvu (Chauru) | Baga-moyo | Coast | TXD 306 | March | Maturity | 3 | -6.742699, 38.668300 |
|  |  |  |  |  |  |  |  |  |
| 24 | Baga-moyo | Baga-moyo | Coast | Local | March | Maturity | 2 | -6.456316, 38.902098 |
|  | Baga-moyo | Baga-moyo | Coast | Local | March | Maturity | 2 | -6.456316, 38.902098 |
|  | Baga-moyo | Baga-moyo | Coast | Local | March | Maturity | 2 | -6.456316, 38.902098 |
|  | Baga-moyo | Baga-moyo | Coast | Local | March | Maturity | 2 | -6.456316, 38.902098 |
|  | Baga-moyo | Baga-moyo | Coast | Local | March | Maturity | 2 | -6.456316, 38.902098 |
|  | Baga-moyo | Baga-moyo | Coast | Local | March | Maturity | 2 | -6.456316, 38.902098 |
|  | Baga-moyo | Baga-moyo | Coast | Local | March | Maturity | 2 | -6.456316, 38.902098 |
|  | Baga-moyo | Baga-moyo | Coast | Local | March | Maturity | 2 | -6.456316, 38.902098 |
|  | Baga-moyo | Baga-moyo | Coast | Local | March | Maturity | 2 | -6.456316, 38.902098 |
|  | Baga-moyo | Baga-moyo | Coast | Local | March | Maturity | 2 | -6.456316, 38.902098 |
|  | Baga-moyo | Baga-moyo | Coast | Local | March | Maturity | 2 | -6.456316, 38.902098 |
|  | Baga-moyo | Baga-moyo | Coast | Local | March | Maturity | 2 | -6.456316, 38.902098 |
|  | Baga-moyo | Baga-moyo | Coast | Local | March | Maturity | 2 | -6.456316, 38.902098 |
|  | Baga-moyo | Baga-moyo | Coast | Local | March | Maturity | 2 | -6.456316, 38.902098 |
|  | Baga-moyo | Baga-moyo | Coast | Local | March | Maturity | 2 | -6.456316, 38.902098 |
|  | Baga-moyo | Baga-moyo | Coast | Local | March | Maturity | 2 | -6.456316, 38.902098 |
|  |  |  |  |  |  |  |  |  |
| 25 | Mombo | Koro-gwe | Tanga | TXD 306 | March | Maturity | 2 | -4.896162, 38.268456 |
|  | Mombo | Koro-gwe | Tanga | TXD 306 | March | Maturity | 2 | -4.896162, 38.268456 |
|  | Mombo | Koro-gwe | Tanga | TXD 306 | March | Maturity | 2 | -4.896162, 38.268456 |
|  | Mombo | Koro-gwe | Tanga | TXD 306 | March | Maturity | 2 | -4.896162, 38.268456 |
|  | Mombo | Koro-gwe | Tanga | TXD 306 | March | Maturity | 2 | -4.896162, 38.268456 |
|  | Mombo | Koro-gwe | Tanga | TXD 306 | March | Maturity | 2 | -4.896162, 38.268456 |
|  | Mombo | Koro-gwe | Tanga | TXD 306 | March | Maturity | 2 | -4.896162, 38.268456 |
|  | Mombo | Koro-gwe | Tanga | TXD 306 | March | Maturity | 2 | -4.896162, 38.268456 |
|  | Mombo | Koro-gwe | Tanga | TXD 306 | March | Maturity | 2 | -4.896162, 38.268456 |
|  | Mombo | Koro-gwe | Tanga | TXD 306 | March | Maturity | 2 | -4.896162, 38.268456 |
|  | Mombo | Koro-gwe | Tanga | TXD 306 | March | Maturity | 2 | -4.896162, 38.268456 |
|  | Mombo | Koro-gwe | Tanga | TXD 306 | March | Maturity | 2 | -4.896162, 38.268456 |
|  | Mombo | Koro-gwe | Tanga | TXD 306 | March | Maturity | 2 | -4.896162, 38.268456 |
|  | Mombo | Koro-gwe | Tanga | TXD 306 | March | Maturity | 2 | -4.896162, 38.268456 |
|  | Mombo | Koro-gwe | Tanga | TXD 306 | March | Maturity | 2 | -4.896162, 38.268456 |
|  | Mombo | Koro-gwe | Tanga | TXD 306 | March | Maturity | 2 | -4.896162, 38.268456 |
|  | Mombo | Koro-gwe | Tanga | TXD 306 | March | Maturity | 2 | -4.896162, 38.268456 |
|  |  |  |  |  |  |  |  |  |
| 26 | Mwama-puli | Igunga | Tabora | TXD 306 | February | Reproductive | 3 | -4.355418, 33.909460 |
|  | Mwama-puli | Igunga | Tabora | TXD 306 | February | Reproductive | 3 | -4.355418, 33.909460 |
|  | Mwama-puli | Igunga | Tabora | TXD 306 | February | Reproductive | 3 | -4.355418, 33.909460 |
|  | Mwama-puli | Igunga | Tabora | TXD 306 | February | Reproductive | 3 | -4.355418, 33.909460 |
|  | Mwama-puli | Igunga | Tabora | TXD 306 | February | Reproductive | 3 | -4.355418, 33.909460 |
|  | Mwama-puli | Igunga | Tabora | TXD 306 | February | Reproductive | 3 | -4.355418, 33.909460 |
|  | Mwama-puli | Igunga | Tabora | TXD 306 | February | Reproductive | 3 | -4.355418, 33.909460 |
|  | Mwama-puli | Igunga | Tabora | TXD 306 | February | Reproductive | 3 | -4.355418, 33.909460 |
|  | Mwama-puli | Igunga | Tabora | TXD 306 | February | Reproductive | 3 | -4.355418, 33.909460 |
|  | Mwama-puli | Igunga | Tabora | TXD 306 | February | Reproductive | 3 | -4.355418, 33.909460 |
|  |  |  |  |  |  |  |  |  |
| 27 | Mwama-puli | Igunga | Tabora | TXD 306 | February | Reproductive | 4 | -4.350908, 33.914491 |
|  | Mwama-puli | Igunga | Tabora | TXD 306 | February | Reproductive | 4 | -4.350908, 33.914491 |
|  | Mwama-puli | Igunga | Tabora | TXD 306 | February | Reproductive | 4 | -4.350908, 33.914491 |
|  | Mwama-puli | Igunga | Tabora | TXD 306 | February | Reproductive | 4 | -4.350908, 33.914491 |
|  | Mwama-puli | Igunga | Tabora | TXD 306 | February | Reproductive | 4 | -4.350908, 33.914491 |
|  |  |  |  |  |  |  |  |  |
| 28 | Mwama-puli | Igunga | Tabora | TXD 306 | February | Reproductive | 4 | -4.349097, 33.916132 |
|  | Mwama-puli | Igunga | Tabora | TXD 306 | February | Reproductive | 4 | -4.349097, 33.916132 |
|  | Mwama-puli | Igunga | Tabora | TXD 306 | February | Reproductive | 4 | -4.349097, 33.916132 |
|  | Mwama-puli | Igunga | Tabora | TXD 306 | February | Reproductive | 4 | -4.349097, 33.916132 |
|  | Mwama-puli | Igunga | Tabora | TXD 306 | February | Reproductive | 4 | -4.349097, 33.916132 |
|  | Mwama-puli | Igunga | Tabora | TXD 306 | February | Reproductive | 4 | -4.349097, 33.916132 |
|  | Mwama-puli | Igunga | Tabora | TXD 306 | February | Reproductive | 4 | -4.349097, 33.916132 |
|  | Mwama-puli | Igunga | Tabora | TXD 306 | February | Reproductive | 4 | -4.349097, 33.916132 |
|  | Mwama-puli | Igunga | Tabora | TXD 306 | February | Reproductive | 4 | -4.349097, 33.916132 |
|  | Mwama-puli | Igunga | Tabora | TXD 306 | February | Reproductive | 4 | -4.349097, 33.916132 |
|  | Mwama-puli | Igunga | Tabora | TXD 306 | February | Reproductive | 4 | -4.349097, 33.916132 |
|  |  |  |  |  |  |  |  |  |
| 29 | Mwama-puli | Igunga | Tabora | TXD 306 | February | Reproductive | 4 | -4.349588, 33.916919 |
|  | Mwama-puli | Igunga | Tabora | TXD 306 | February | Reproductive | 4 | -4.349588, 33.916919 |
|  | Mwama-puli | Igunga | Tabora | TXD 306 | February | Reproductive | 4 | -4.349588, 33.916919 |
|  | Mwama-puli | Igunga | Tabora | TXD 306 | February | Reproductive | 4 | -4.349588, 33.916919 |
|  | Mwama-puli | Igunga | Tabora | TXD 306 | February | Reproductive | 4 | -4.349588, 33.916919 |
|  | Mwama-puli | Igunga | Tabora | TXD 306 | February | Reproductive | 4 | -4.349588, 33.916919 |
|  | Mwama-puli | Igunga | Tabora | TXD 306 | February | Reproductive | 4 | -4.349588, 33.916919 |
|  | Mwama-puli | Igunga | Tabora | TXD 306 | February | Reproductive | 4 | -4.349588, 33.916919 |
|  | Mwama-puli | Igunga | Tabora | TXD 306 | February | Reproductive | 4 | -4.349588, 33.916919 |
|  | Mwama-puli | Igunga | Tabora | TXD 306 | February | Reproductive | 4 | -4.349588, 33.916919 |
|  |  |  |  |  |  |  |  |  |
| 30 | Mwama-puli | Igunga | Tabora | TXD 306 | February | Reproductive | 4 | -4.347134, 33.925409 |
|  | Mwama-puli | Igunga | Tabora | TXD 306 | February | Reproductive | 4 | -4.347134, 33.925409 |
|  | Mwama-puli | Igunga | Tabora | TXD 306 | February | Reproductive | 4 | -4.347134, 33.925409 |
|  | Mwama-puli | Igunga | Tabora | TXD 306 | February | Reproductive | 4 | -4.347134, 33.925409 |
|  | Mwama-puli | Igunga | Tabora | TXD 306 | February | Reproductive | 4 | -4.347134, 33.925409 |
|  | Mwama-puli | Igunga | Tabora | TXD 306 | February | Reproductive | 4 | -4.347134, 33.925409 |
|  | Mwama-puli | Igunga | Tabora | TXD 306 | February | Reproductive | 4 | -4.347134, 33.925409 |
|  | Mwama-puli | Igunga | Tabora | TXD 306 | February | Reproductive | 4 | -4.347134, 33.925409 |
|  | Mwama-puli | Igunga | Tabora | TXD 306 | February | Reproductive | 4 | -4.347134, 33.925409 |
|  | Mwama-puli | Igunga | Tabora | TXD 306 | February | Reproductive | 4 | -4.347134, 33.925409 |
|  |  |  |  |  |  |  |  |  |
| 31 | Ngana | Kyela | Mbeya | TXD 306 | February | Maturity | 2 | -9.584835, 33.6997167 |
|  | Ngana | Kyela | Mbeya | TXD 306 | February | Maturity | 2 | -9.584835, 33.6997167 |
|  | Ngana | Kyela | Mbeya | TXD 306 | February | Maturity | 2 | -9.584835, 33.6997167 |
|  | Ngana | Kyela | Mbeya | TXD 306 | February | Maturity | 2 | -9.584835, 33.6997167 |
|  | Ngana | Kyela | Mbeya | TXD 306 | February | Maturity | 2 | -9.584835, 33.6997167 |
|  |  |  |  |  |  |  |  |  |
| 32 | Ngana | Kyela | Mbeya | TXD 306 | February | Maturity | 2 | -9.587494, 33.691116 |
|  | Ngana | Kyela | Mbeya | TXD 306 | February | Maturity | 2 | -9.587494, 33.691116 |
|  | Ngana | Kyela | Mbeya | TXD 306 | February | Maturity | 2 | -9.587494, 33.691116 |
|  | Ngana | Kyela | Mbeya | TXD 306 | February | Maturity | 2 | -9.587494, 33.691116 |
|  | Ngana | Kyela | Mbeya | TXD 306 | February | Maturity | 2 | -9.587494, 33.691116 |
|  | Ngana | Kyela | Mbeya | TXD 306 | February | Maturity | 2 | -9.587494, 33.691116 |
|  |  |  |  |  |  |  |  |  |
| 33 | Kilasilo | Kyela | Mbeya | SUPA | March | Maturity | 2 | -9.587998, 33.806142 |
|  | Kilasilo | Kyela | Mbeya | SUPA | March | Maturity | 2 | -9.587998, 33.806142 |
|  | Kilasilo | Kyela | Mbeya | SUPA | March | Maturity | 2 | -9.587998, 33.806142 |
|  | Kilasilo | Kyela | Mbeya | SUPA | March | Maturity | 2 | -9.587998, 33.806142 |
|  | Kilasilo | Kyela | Mbeya | SUPA | March | Maturity | 2 | -9.587998, 33.806142 |
|  |  |  |  |  |  |  |  |  |
| 34 | Llilido | Mikin-dani | Mtwara | TARI RIC 1 | March | Maturity | 2 | -10.352557, 39.704874 |
|  | Llilido | Mikin-dani | Mtwara | TARI RIC 2 | March | Maturity | 2 | -10.352557, 39.704874 |
|  | Llilido | Mikin-dani | Mtwara | TARI RIC 3 | March | Maturity | 2 | -10.352557, 39.704874 |
|  | Llilido | Mikin-dani | Mtwara | TARI RIC 4 | March | Maturity | 2 | -10.352557, 39.704874 |
|  | Llilido | Mikin-dani | Mtwara | TARI RIC 5 | March | Maturity | 2 | -10.352557, 39.704874 |
|  |  |  |  |  |  |  |  |  |
| 35 | Lilido | Mikin-dani | Mtwara | Komboka | March | Maturity | 2 | -10.351676, 39.705414 |
|  | Lilido | Mikin-dani | Mtwara | Komboka | March | Maturity | 2 | -10.351676, 39.705414 |
|  | Lilido | Mikin-dani | Mtwara | Komboka | March | Maturity | 2 | -10.351676, 39.705414 |
|  | Lilido | Mikin-dani | Mtwara | Komboka | March | Maturity | 2 | -10.351676, 39.705414 |
|  | Lilido | Mikin-dani | Mtwara | Komboka | March | Maturity | 2 | -10.351676, 39.705414 |
|  |  |  |  |  |  |  |  |  |
| 36 | Lilido | Mikin-dani | Mtwara | TARI RIC 1 | March | Maturity | 2 | -10.350944, 39.706175 |
|  | Lilido | Mikin-dani | Mtwara | TARI RIC 2 | March | Maturity | 2 | -10.350944, 39.706175 |
|  | Lilido | Mikin-dani | Mtwara | TARI RIC 3 | March | Maturity | 2 | -10.350944, 39.706175 |
|  | Lilido | Mikin-dani | Mtwara | TARI RIC 4 | March | Maturity | 2 | -10.350944, 39.706175 |
|  | Lilido | Mikin-dani | Mtwara | TARI RIC 5 | March | Maturity | 2 | -10.350944, 39.706175 |
|  |  |  |  |  |  |  |  |  |
| 37 | Lilido | Mikin-dani | Mtwara | TXD 306 | March | Maturity | 2 | -10.353980, 39.705278 |
|  | Lilido | Mikin-dani | Mtwara | TXD 306 | March | Maturity | 2 | -10.353980, 39.705278 |
|  | Lilido | Mikin-dani | Mtwara | TXD 306 | March | Maturity | 2 | -10.353980, 39.705278 |
|  | Lilido | Mikin-dani | Mtwara | TXD 306 | March | Maturity | 2 | -10.353980, 39.705278 |
|  | Lilido | Mikin-dani | Mtwara | TXD 306 | March | Maturity | 2 | -10.353980, 39.705278 |
|  | Lilido | Mikin-dani | Mtwara | TXD 306 | March | Maturity | 2 | -10.353980, 39.705278 |

*According to the IRRI Standard Evaluation System for rice, nine growth stages were considered: 1-Germination, 2-Seedling, 3-Tillering, 4-Stem elongation, 5-Booting, 6-Heading, 7-Milk stage, 8- Dough stage, and 9-Mature grain.

**Bacterial Leaf Blight Disease score scale: 1=0-3%, 2=4-6%, 3=7-12%, 4=13-25%, 5=26-50%, 6=51-75%, 7= 76-87%, 8=88-94%, 9=95-100% (% of leaf area affected by the disease)

**Supplementary file 1c. Sequences of *Xa1* and *Xa4* genes from *Oryza sativa* cv. Komboka.** Capital letters represent exons, while small letters represent introns. Start and stop codons are highlighted in yellow and red, respectfully.

> *Oryza sativa* cv. Komboka *Xa1*

ATGGAGGAGGTGGAAGCCGGTTTGCTGGAGGGCGGGATCAGGTGGCTGGCGGAGACCATCCTGGATAACCTGGACGCCGACAAGCTGGATGAGTGGATTCGCCAGATTAGGCTCGCCGCTGACACCGAGAAGCTACGGGCTGAGATCGAGAAGGTGGATGGGGTGGTCGCTGCCGTGAAGGGGAGGGCGATCGGGAACAGGTCGCTGGCCCGATCTCTCGGCCGTCTCAGGGGGTTGCTGTACGACGCCGACGATGCGGTCGACGAGCTCGACTACTTCAGGCTCCAGCAGCAGGTCGAGGGAGGAGgtactgtctttgcatatatccgtgccttttaattaagtttgcaagctgcgttgcctgcaacaatggcgtattggcgtcagtttccaatccatgcttgtgctacagTTACTACACGGTTTGAGGCTGAAGAGACGGTCGGAGATGGAGCAGAGGACGAGGACGATATTCCGATGGACAATACTGATGTACCGGAGGCAGTGGCGGCAGGCAGCAGCAAGAAACGGTCCAAGGCATGGGAACACTTTACTACCGTAGAGTTCACTGCTGACGGGAAGGATTCTAAAGCACGGTGCAAGTACTGCCACAAGGACCTATGTTGCACATCTAAGAACGGGACATCAGCTTTGCGCAACCATCTCAATGTTTGCAAGAGGAAACGTGTAACAAGTACTGACCAACCGGTAAATCCATCAAGgtaatgctaatggagttctgaatttagtgtaaatccgttgaagtgtaaatttggcccgttacatctgcttaagatctcattctgtctctaatcttctaatagccaactcatggtcattttttttcctaatatatagtaccggtgatggtgcaccaaatgtaattagatgcaaggaaacaaaagtgaacaattgtatatatcaaatataattatatctaaaacatgagtagtgtatcaaatccaattctttcaaaaatctactatgcaaaattgagtgacaaaatctgctgccttttttttttacagaaagcaaccaattaatataagtcaaatataaaaacgctttgtagtctccaataaaatagctcattgtttcgtttatacttatgtttataaatttaaatttaaaacttaattttggagttgattttgtggttttcttttcatcctattttattttacaacatttgattttgaatagttaagaatgcgtatataaaaattttacccataagttattttttaaattgttaataaatcgtaaggataatcataagtataagtgaaacgattcgctcttcatctacttaagattgcgttatattgctgacctttctaatcgcctaaccacgatcacatgctcttccagTGCCGGTGAGGGTGCATCAAATGCAACTGGTAATTCAGTTGGCAGAAAAAGGATGAGAATGGATGGGACTTCAACACACCACGAGGCAGTTAGCACGCACCCTTGGAACAAGGCTGAACTTTCCAACAGGATCCAATGCATGACTCATCAGTTAGAAGAGGCTGTAAATGAGGTTATGAGGCTATGTCGATCCTCAAGTTCAAACCAGAGTCGACAGGGTACACCACCGGCCACAAATGCAACAACATCGTCTTATCTTCCGGAGCCCATAGTGTATGGGAGGGCTGCAGAGATGGAAACCATCAAACAGCTGATCATGAGCAATAGATCTAATGGCATAACCGTCCTGCCAATTGTAGGCAATGGAGGGATAGGAAAAACCACTTTGGCGCAACTGGTCTGCAAAGATCTGGTAATTAAAAGTCAGTTTAATGTTAAGATATGGGTGTATGTATCTGATAAATTTGATGTAGTTAAGATTACAAGGCAGATTTTGGATCATGTCTCCAACCAGAGCCACGAAGGAATAAGCAACCTTGATACGCTTCAGCAGGATCTTGAGGAACAAATGAAATCTAAGAAGTTCCTCATTGTCTTAGATGATGTGTGGGAAATCCGTACAGATGACTGGAAAAAACTACTGGCTCCTTTAAGACCTAAAGATCAGGTGAATTCGTCACAGGAAGAGGCAACAGGTAATATGATAATTTTGACAACTCGTATACAGAGTATTGCCAAAAGTCTTGGAACAGTACAATCAATTAAGTTAGAAGCTCTGAAAGATGACGATATATGGTCACTATTTAAAGTGCATGCTTTTGGTAATGATAAACATGATAGTAGTCCAGGCTTACAGGTTCTTGGGAAGCAAATTGCTAGCGAGCTAAAAGGCAACCCACTGGCAGCAAAAACTGTGGGTTCACTATTAGGAACGAATCTTACCATCGATCATTGGGATAGCATTATAAAGAGTGAAGAATGGAAATCCCTGCAACAAGCTTATGGCATCATGCAAGCGCTGAAGTTGAGCTATGATCATCTATCCAACCCCTTACAGCAATGCGTCTCTTATTGTTCTCTTTTCCCCAAGGGTTATTCTTTCAGCAAAGCACAACTAATACAAATATGGATTGCTCAAGGATTTGTGGAAGAATCCAGTGAGAAGTTGGAGCAGAAAGGATGGAAATATCTAGCTGAGTTGGTAAATTCGGGTTTCCTTCAGCAAGTTGAAAGCACACGGTTTTCATCAGAATATTTTGTTATGCACGATCTTATGCATGATTTAGCGCAAAAGGTTTCACAAACAGAATATGCAACTATAGATGGCTCAGAGTGCACAGAGTTAGCCCCAAGTATACGCCATTTGTCAATAGTAACTGATTCTGCATACCGCAAGGAGAAATATAGAAACATATCTCGTAATGAGGTGTTTGAGAAAAGGTTGATGAAAGTTAAGTCAAGGAGTAAGTTGAGGTCACTGGTATTAATTGGGCAATATGATTCTCATTTTTTTAAATATTTCAAAGATGCTTTCAAGGAAGCACAACATCTGCGACTGCTGCAGATCACTGCAACTTATGCTGATTCTGATTCATTTCTCTCCAGTTTGGTAAATTCTACACATCTCCGGTATCTGAAAATTGTGACCGAAGAATCCGGCAGAACTTTGCCCCGATCTCTAAGGAAGTATTACCATCTTCAAGTACTAGATATTGGCTATAGATTTGGAATTCCCCGTATATCTAATGATATAAATAATCTTCTCAGCCTGCGGCATCTTGTTGCATATGATGAAGTGTGTTCTTCCATTGCTAACATTGGTAAAATGACCTCACTTCAGGAACTAGGCAATTTTATTGTTCAGAATAATTTAAGTGGTTTTGAGGTGACACAATTGAAATCCATGAACAAGCTTGTACAACTTAGTGTGTCTCAGCTTGAAAATGTTAGAACTCAGGAGGAGGCATGTGGGGCAAAACTGAAAGACAAACAACACTTAGAAAAGCTACATTTGTCCTGGAAGGATGCATGGAATGGATATGACAGTGACGAAAGCTATGAAGATGAATACGGCAGTGATATGAATATAGAAACAGAAGGGGAGGAACTGTCAGTTGGTGATGCCAATGGTGCCCAAAGCTTACAACATCACAGTAATATAAGCTCTGAACTTGCTTCAAGTGAGGTGCTCGAAGGTCTTGAACCACATCACGGCCTCAAGTATCTACGGATATCTGGGTATAATGGATCTACCTCCCCAACTTGGCTTCCTTCTTCACTTACCTGTCTGCAAACACTTCATCTAGAAAAATGTGGAAAATGGCAAATACTTCCTTTAGAAAGGCTAGGGTTACTTGTAAAGCTCGTGTTGATCAAAATGAGGAATGCAACAGAACTCTCAATCCCTTCACTGGAGGAGCTTGTGTTAATTGCATTGCCAAGCTTGAACACATGCTCCTGCACTTCCATCAGGAACTTGAACTCCAGTTTAAAGGTTCTGAAAATTAAGAATTGCCCTGTACTGAAGGTATTTCCCTTGTTTGAGATTTCCCAGAAATTTGAAATCGAGCGGACGTCGTCATGGTTGCCCCATCTTAGCAAGCTTACCATCTATAATTATCCTCTTTCCTGTGTGCACAGTTCTCTGCCACCTTCCGCAATCAGTGGTTATGGAGAATATGGAAGGTGTACCCTTCCGCAATCACTTGAGGAACTTTACATCCATGAGTATTCTCAAGAAACTCTGCAGCCCTGCTTTTCAGGGAACCTCACTCTCCTGAGAAAATTACATGTACTGGGAAACTCAAATTTAGTGTCTCTGCAGCTCCATTCCTGCACAGCACTCGAAGAGTTGATAATTCAAAGCTGTGAGTCTCTTAGTTCTCTGGATGGCTTGCAATTGCTTGGCAATCTCAGGTTGCTGCAGGCACATAGATGCCTCAGTGGTCATGGAGAAGATGGAAGGTGTATCCTTCCGCAATCACTTGAGGAACTTTACATCCATGAGTATTCTCAAGAAACTCTGCAGCCCTGCTTTTCAGGGAACCTCACTCTCCTGAGAAAATTACATGTACTGGGAAACTCAAATTTAGTGTCTCTGCAGCTCCATTCCTGCACAGCACTCGAAGAGTTGATAATTCAAAGCTGTGAGTCTCTTAGTTCTCTGGATGGCTTGCAATTGCTTGGCAATCTCAGGTTGCTGCAGGCACATAGATGCCTCAGTGGTCATGGAGAAGATGGAAGGTGTATCCTTCCGCAATCACTTGAGGAACTTTACATCCATGAGTATTCTCAAGAAACTCTGCAGCCCTGCTTTTCAGGGAACCTCACTCTCCTGAGAAAATTACATGTACTGGGAAACTCAAATTTAGTGTCTCTGCAGCTCCATTCCTGCACAGCACTCGAAGAGTTGATAATTCAAAGCTGTGAGTCTCTTAGTTCTCTGGATGGCTTGCAATTGCTTGGCAATCTCAGGTTGCTGCAGGCACATAGATGCCTCAGTGGTCATGGAGAAGATGGAAGGTGTATCCTTCCGCAATCACTTGAGGAACTTTACATCCATGAGTATTCTCAAGAAACTCTGCAGCCCTGCTTTTCAGGGAACCTCACTCTCCTGAGAAAATTACATGTACTGGGAAACTCAAATTTAGTGTCTCTGCAGCTCCATTCCTGCACAGCACTCGAAGAGTTGATAATTCAAAGCTGTGAGTCTCTTAGTTCTCTGGATGGCTTGCAATTGCTTGGCAATCTCAGGTTGCTGCAGGCACATAGATGCCTCAGTGGTCATGGAGAAGATGGAAGGTGTATCCTTCCGCAATCACTTGAGGAACTTTACATCCATGAGTATTCTCAAGAAACTCTGCAGCCCTGCTTTTCAGGGAACCTCACTCTCCTGAGAAAATTACATGTACTGGGAAACTCAAATTTAGTGTCTCTGCAGCTCCATTCCTGCACAGCACTCGAAGTGTTGATAATTCAAAGCTGTGAGTCTCTTAGTTCTCTGGATGGCTTGCAATTGCTTGGCAACCTCAGGTTGCTGCAGGCACATAGATGCCTCAGTGGTCATGGAGAAGATGGAAGGTGTATCCTTCCGCAATCACTTGAGGAACTTTACATCCATGAGTATTCTCAAGAAACTCTGCAGCCCTGCTTTTCAGGGAACCTCACTCTCCTGAGAAAATTACATGTACTGGGAAACTCAAATTTAGTGTCTCTGCAGCTCCATTCCTGCACAGCACTCGAAGTGTTGATAATTCAAAGCTGTGAGTCTCTTAGTTCTCTGGATGGCTTGCAATTGCTTGGCAATCTCAGGTTGCTGCAGGCACATAGATGCCTCAGTGGTCATGGAGAAGATGGAAGGTGTATCCTTCCGCAATCACTTGAGGAACTTTTCATCAGTGAGTATTCTCTAGAAACTCTGCAGCCCTGCTTCCTGACGAATCTCACCTGCTTAAAACAATTAAGGGTATCAGGCACCACAAGTTTCAAATCTCTAGAACTGCAATCATGCACTGCACTCGAACATTTGAAGATTCAAGGTTGTGCGTCGCTTGCTACATTGGAGGGGTTGCAATTCCTCCACGCCCTCAGGCATATGGAAGTATTCAGATGCCCTGGCTTGCCTCCATATTTGGGGAGTTCGTCAGAGCAGGGCTATGAGCTATGCCCACGACTGGAAAGGCTCGACATCGATGACCCCTCTATCCTTACCACGTCGTTCTGCAAGCACCTCACCTCCCTCCAACGCCTAGAGCTTAACTATTGCGGAAGTGAAGTGGCAAGACTAACGGATGAGCAAGAGAGAGCGCTTCAGCTCCTCACGTCCCTGCAAGAGCTCCGGTTTAAGTATTGCTACAATCTCATAGATCTTCCTGCGGGGCTCCACAGCCTTCCCTCCCTCGAGAGGTTGGAGATCCGGAGTTGCAGGAGCATCGCGAGGCTGCCGGAGAAGGGCCTCCCACCTTCGTTCGAAGAACTGGATATCATCGCTTGCAGTAATGAGCTAGCTCAGCAGTGCAGAACTCTAGCAAGCACTCTGAAGGTCAAAATTAATGGGGGATATGTGAACTGA

> *Oryza sativa* cv. Komboka *Xa4*

ATGGCAGCAGCCGCCGCACTTTCTGCGGTGGCGCCTGGGCAGCGGCCCGCGGACTGCCCGAGCGAGTGCGGCGGCGTGGACATCCCCTACCCATTCGGCGTGGACAACTGCTCCTGGCCGGGTCCTGATGACTTCACCATCATCTGCAAATATAGCAGGCCGTACTACAGGGGCGCGGAGATCGTGAACATCTCGGTGGAGGCAGGGGAGATGCGCGTCTACTCTCCCGTGGTCTCCCAGTGCTACAACTCATCCAACACCACGGACTCTGACGGATTCGAGTTCTTGCGGCTCAACATCACCAACACGCCGTTCCTGGTCGCTCCGGAGAGAAACGAGTTCACGGCCATCGGCTGTGCCACGTTGGCGTGGCTATGGGGCAGAGACGATGGGAGCTACTTGACCGGCTGCATCTCGACGTGCGCGAGCTTGGCGACGGCTGCCAAGGACCGTGACCCATGTACGGGGCTGGGTTGCTGCCAGGTGCCCTCCATCCCGGCCAACCTTAGCGTCTTAAATATTTCCTTGGGCACAGGCATCGCCAACGTTGCCTGGGAAGAAAGTCCCTGTAGCTACGCCTTCGTGGCCGAAAAACACTGgtacgtaactaagtacgaacacatacctactacttcatacatctatttttatttttgatagtgatatttctaaatcagaaaaatttacttttgataggtatatttcaatccaaccatttattcttttaatgactttcttggatttaatgcgtgaatctccattctttcacacaaaattggctacatgagcatcaagaaatgtaaatattattatatcgcttgtttacgaggaataactaatactccactagtatgtttaaatggatgataagtagaattatttattcttggtcattgtgccaagataaaatatgactatcaaaggactctatctatcgtctagactcacgaatttgaatacatatatatggatggatacacggatctatatctctatctctactattttaagttttcgttaatactttagtacgtagtttgtatttgagtcggtttttaaatccgttcgcttttcaaaatacataaggagtcatacaaggaattcttttaaaaaacttgcatgctaactgggatgagcgagaattagactcctaattgcatcagaattcctaccaagacataagaaaacaacgtatatgatatggttattatccgtcccaacttagatatcaaatactatctcaaagtaaaattcacatttactttttagataaaataaatctaataataatatgattaaaatagcattcacatgttgcaacgcacaagtatttttctgtatatatagatatatattcaaaacccaatacgttaagttcatcttataatatgaaaaagagaatatactccctccgtccctaaatatttgacgccgttaacttttttaaatatgtttgaccgttcgtcttattcaaaaaaatttaagtaattattcattctttttgattcattgttaaatgtacttttatgtatatatatagttttaaatatttcataaaagttttcaaataagattaacggtcaaatatatttaaaaaaatcaacggtgtcaaatatttagggacggagggagtatataagtacacaaaatccattagtattataattatatggatctaattattaatgataatgattattcactctatttggtgtccatactagtttaatttattttgatgtatatccttatatgtgacggttctttaaaagaaacagcatgtacgtgtatcttcatatccgttgtttttactgagaacagcacatatatatcgacatcatatttgcgttggttctcaatgaaacaggcatatgagtgcagcataatatgcaatattttacacatgcatggtggatatttttatatgtgcatattgtgccgtcacttatatgtgcatatttttaataacttttaaaattatatgaagttggatgaagataaattttactatactaagttcatagagttcaacgagatgtaacaactttgcagataattgatatatagctttttcacttgggattagttatatgtccgaatatactttagaaactcttggaccaatttggatatataaatagacttttaaaattttaaacgacttcgtacgcgctatacaaaagttatacgagaccatcggatggcttattggcttattggggaaataagccaaacggcatatttacaaacaaaaaataatttgtgaataaaacttttatatacatgttcttaataatctaaaaacaaaggctgaaaaataaacttcaatgaaaaaacctcaaaatcaactccaaatttaaagttgaaaatttaaattttggctgataagtataagcataagcgaaaagatgaggccgataaagcttgacgagatctaaaattttgtagtttataaccttttcatttgaactcatttggggtgcaacatacgcattatgaatttttgacgggagtagtaacaagatacatttgttttctaacgacaaatgctcgatagttgagatgataagaaagctgcacatgcagataaaaggtctcggatttgaattccacatttcccatgtgcacgtatttcgcatgaaaaatctcgtgacttgtgacttgcaaaattagaaatactactctgggttttaaatttgcacctaggaaattcaaaatgtagcaccactgtatttgccggttttactttagaatcgaaatataatatgcctaacaccaaatggtttaagaattaagtgggttaacaaaataaatggcacatggatgactttcatgtgtgatgattcttaataaaaattagcacatatataattttcatttgttacagttcttaataaaaacctgaacatatgaaggtatatatgtgccggttcttttaactttggctctatatgcatcttggggtgaatgagagattaataccttatttgtgttagtttttggataatcagtatatatagtattgtcacttatttaatgtttttcagccgtgaactgattgtgcacctgttgtccgtatgtgaatcagGTATAATTTCAACAGGCAAGATTTCGAACGTGACGGTAAAAGCTTTGAACACCGGGATGAGAAAATGGTAGTTCCGACGGTGCTTGACTGGGCCATCAGGAAAAATGGGTCGTGCCCGTCAACCGGACAGGGTGCTCCTGCCTGTAAGAGCGAACATAGTGAGTGCGTCAACGCCACCAACGGCAAGGGCTACCTCTGCAATTGCTCCAGGGGATACGCCGGCAATCCCTATAGGGACGATGGATGCAAAAgttagttacgttcttaattagctcgctagctatcacctaagcatagaattcttctctaattttattttggatgctagattagtatttacagtacgtggtgatgattaatgacgagcatgcttgcttagtgtttaaatttacttatttatgacttaattgttctaactctttaatttgattgtttatcaccaatacgtctgtagATATTAATGAGTGTAAGGAACCTTCTATTACTTGTTACGGTGGTAGCACATGCCAGGACACAGACGGCAGTTACGAGTGCAAATGCCAATTTGGATATAGAGGCGATGGCAGGAAGAATGAGAGTCAGAAGGGACGATGCCAACCCATAATTCCCGCTGCTATAGCCAATGCAATAGgtaaaccgatagagtgttaattcttaactactccaacacgtagcaatataaatttaatttgctacctattagcaaatgtggatcaacactgtatatctttatcttatgtttctttgtatgcagCGATTGTTTGCATTGTGATTGTTCTCTTGGGCCTTTTCTGGTTGCCTAAGAGATGGAAGCGGAGAGTGTTCTTCGATAATAACGGTGGTCGCCTACTGAAGGATATGGACATCATCGTTTTCACTGAGAAGGAACTCAACAAGATAACAAACAAGAAGCGCACTAAGATTGGAGAGGGCGCCTTTGGCGAGGTCTACAAGGGGAACCACAATAACCAACCGGTTGCTGTCAAGTACTCCATCGCAAAGAACATGACTCAAACACATTACAAAGACGTCGTCGAGAGCATAAACCAAAACGTATTCCAGACTGTTTTTCGTCAGTCAAAAGTTCCACCATCAACACCGGGCCAGAATGCAGTCGTCAACGAGATAAAGGTTCAGCTGCAGATCCGGCACCCCAACATTGTCCGCCTCATCGGGTGCTGTATGGAAACAGAGGTCCCCATGCTTGTCTTTGAGTTCATCCCTAACGGGAGCCTCGAGACAGTTCTCCATGGTATCGATCGATGCAGCCTTTCTCTCCAGCAACGCCTGGACATCGCGATCGGCTCTGCAGAGGCTCTTGCCTACATGCATTGGCACGGCCATCATCAGATCATACATGGGGATATCAAGCCTGGCAACATTCTCCTTGGTGACAATCTCATGCCTAAGGTCTCCGACTTTGGATCATCTGAGCTCACGTTGAAAGTCAAGCGTGCAGGGAAGTGGAACGTATATGCTGACATGAACTACATCGACCCTGTGTACATCAAGACAGGCGATTTCACGGATAAGAGCGATGTATATAGTTTTGGGGTTGTGCTCCTAGAGCTCATCACCAGGAAGAAGGCCAAGTATGACGATAGAAGCCTCCCGGTAGAATTTGTCAGCCATTATGAGGATGAAGACACAAGGAGGAAAATGTATGACCAGGACATGTTGCCTACCGAGGCCTCGCATCCTCACTGCATGGAGTGTCTTGACAGAATGGCCGATATTGTGCTCCGTTGTCTTGAAAATGAAGTGGGCAAGAGGCCAACCATGGCTGAGGTGCTAGAGGAGCTTAAGAAGTTGTTACCATTGCTAACCACGACGCCGGTCGAACTCGTGTAG

**Supplementary file 1d. Characteristics of the TALome of *Xanthomonas oryzae* pv. *oryzae* Tanzanian iTz strains**

| **Name** | **Site** | **Year** | **Major Vir TALe*** | **Number of TALes** | **iTALe** |
| --- | --- | --- | --- | --- | --- |
| TzDak11-1 | Dakawa | 2011 | TalC | 9 | - |
| TzDak11-2 | Dakawa | 2011 | TalC | 9 | - |
| TzDak18-1 | Dakawa | 2018 | TalC | 9 | - |
| iTzDak19-1 | Dakawa | 2019 | PthXo1-like | nd | + |
| iTzDak19-2 | Dakawa | 2019 | PthXo1-like | nd | + |
| iTzDak19-3 | Dakawa | 2019 | PthXo1-like | >16 | + |
| iTzLuk21-1 | Lukenge | 2021 | PthXo1-like | >16 | + |
| iTzLuk21-2 | Lukenge | 2021 | PthXo1-like | >16 | + |
| iTzLuk21-3 | Lukenge | 2021 | PthXo1-like | nd | + |
| iTzLuk21-4 | Lukenge | 2021 | PthXo1-like | >16 | + |
| iTzLuk21-5 | Lukenge | 2021 | PthXo1-like | nd | + |

*TALe inducing *SWEET11*, *SWEET13* or *SWEET14*.

**Supplementary file 1e. *OsSWEET* promoter sequences for the rice varieties Komboka and Kitaake.**

***OsSWEET11a* sequence. PthXo1 EBE is highlighted in yellow. First exon is highlighted in grey.**

>OsSWEET11a_Komboka_ 1756 bp.

CTCTCTAAGAATAGGCATATTATGGTTTAATTAGCTAGACAAGAAATTAGTTAAGGTTCTTAATGAAGATTCCGTCACTTTTGCTAGCTTTGAAAACCTGCAGAGTGAATTGTGCAAAACATCTTGGCATGTTGGTGTTAGTGGTACAACAGATGCTGATACAAAGAAATTAATGCTGCAATTGTTAGAAGCTCTTTTTTTTTTCTCTCTTTTGAACACCAAAGCACTTTTGTTCATGGTGAAAGGGACTACCCTTCTCTTTCATATGTTCCCTTCTTCCTCTGTTCTTTTCATGAGATTTATTTGATGACTTCCCTCTGCTCTGTTATTTTCATGTTCTTTGATAAATGTGCTGATTGTATTATCAGTGTTATTGCACTCAATTCATTCTTGAACAGTGTGTGAATTAACTCTTTGTTCGATTTGAGCTTAGTCACTTGATTGCACACGAACTACTCTGCAATTCTTTTCTGACGATAGAAGTCGATTGATGGATCACCCAACATGTTACTAATTAAGTTGCATCATTGTCCATGGTTGTACATCCTTCTACAAATAAAACTACACAAATCAAGAAAATTTTCAATAACATTTCAACTATTGTAACAAGTAAAAGAAACCTATATGAGAGCTCCAGCTCTCCAAATGGCAACAGACACACTGAGTGGTCATACGTGTCATATTGCCCCTCAGTTATGCATTCATATGACCACATATTCAGAGTAGTGGAGAGAGGGACAGATCTAGAGGTAGAAAAAGAAAATTCATATAAATGATATATCAGAGTGAAAAAGAAATATCAAGCACAAGAAAAAAAAAAGCAAAGGTTAGATATGCATCTCCCCCTACTGTACACCACCAAAAGTGGAGGGTCTCCAACTATATAAACACTGAGCCATGGCCAAGGCCAAACCACACATGCAGTTGTAGTAGCACTTAAGCCTTCCTCTCTAGCTAGCATCTCTTGTGTCAGGAAGTTGGAAGGGATTTCTGGCTAGTTTCTAGCTGGTGTCTCCTCTCCTCTTCCTAACCTTCTCACTGATTAACACCTTAGAGTTAGTTAATAACCTTCATCACCAGTAGCAATGGCAGGAGGTTTCTTGTCCATGGCTAACCCGGCGGTCACCCTCTCCGGTGTTGCAGGTAAAGCATGCAACCAATGCATAATGCTCAAACTTAATTTCATCATTATCATCATCATCATCTTCACAGCCATGATCATCCATGGACAAATGCAACTGAAGATCATTTTAGTTTTCATATGCTAATGATCAAATTCAGGTTAATTGCTGTTTAATTTCTCCATACACTAGTTGTCTGCACCATTGCATTGTGCACAGCACACACACGCTTTTGATGCTTCTAGGAATGCATATCTGTTCAGCAGTTCACACAGTGCAGCAGGGCAATGTTGTTAAAAAATCTTCTCCTTTTTTTTTATGTCCTTGTATTCTTGAGCTTTCTGTCTCCATTGATCTGCTTTTTTCTTGTTTACAAGTGATGGGCACAAGTCACTTCCCTTAGCTTCAGCTCATGCATGGAGCAGGAATCTCACTTCAAAAGACCTAGCACTTTTTCTCTCTTCACCTTTTTGCCTCAACACATGCCCAGTTTCTGGCCACACAAACATAAACACATATACTATCTAGCTGCATAATTGCATCAAATTAAGCAGGGTTTGTTTCAGCTAGGAATTCCACACATAGGTCATTAATTAGTATTGCCAACTTTCTCAACATGCATGCACTCTAGTACT

> OsSWEET11a_Kitaake_OsativaKitaake_499.genome_Chr8 Chr8: 27602383..27604137 (-strand) class=match length=1755 bp

CTCTCTAAGAATAGGCATATTATGGTTTAATTAGCTAGACAAGAAATTAGTTAAGGTTCTTAATGAAGATTCCGTCACTTTTGCTAGCTTTGAAAACCTGCAGAGTGAATTGTGCAAAACATCTTGGCATGTTGGTGTTAGTGGTACAACAGATGCTGATACAAAGAAATTAATGCTGCAATTGTTAGAAGCTCTTTTTTTTCTCTCTTTTGAACACCAAAGCACTTTGTTCATGGTGAAAGGGACTACCCTTCTCTTTCATATGTTCCCTTCTTCCTCTGTTCTTTTCATGAGATTTATTTGATGACTTCCCTCTGCTCTGTTATTTTCATGTTCTTTGATAAATGTGCTGATTGTATTATCAGTGTTATTGCACTCAATTCATTCTTGAACAGTGTGTGAATTAACTCTTTGTTCGATTTGAGCTTAGTCACTTGATTGCACACGAACTACTCTGCAATTCTTTTCTGACGATAGAAGTCGATTGATGGATCACCCAACATGTTACTAATTAAGTTGCATCATTGTCCATGGTTGTACATCCTTCTACAAATAAAACTACACAAATCAAGAAAATTTTCAATAACATTTCAACTATTGTAACAAGTAAAAGAAACCTATATGAGAGCTCCAGCTCTCCAAATGGCAACAGACACACTGAGTGGTCATACGTGTCATATTGCCCCTCAGTTATGCATTCATATGACCACATATTCAGAGTAGTGGAGAGAGGGACAGATCTAGAGGTAGAAAAAGAAAATTCATATAAATGATATATCAGAGTGAAAAAGAAATATCAAGCACAAGAAAAAAAAGCAAAGGTTAGATATGCATCTCCCCCTACTGTACACCACCAAAAGTGGAGGGTCTCCAACTATATAAACACTGAGCCATGGCCAAGGCCAAACCACACATGCAGTTGTAGTAGCACTTAAGCCTTCCTCTCTAGCTAGCATCTCTTGTGTCAGGAAGTTGGAAGGGATTTCTGGCTAGTTTCTAGCTGGTGTCTCCTCTCCTCTTCCTAACCTTCTCACTGATTAACACCTTAGAGTTAGTTAATAACCTTCATCACCAGTAGCAATGGCAGGAGGTTTCTTGTCCATGGCTAACCCGGCGGTCACCCTCTCCGGTGTTGCAGGTAAAGCATGCAACCAATGCATAATGCTCAAACTTAATTtcatcatcatcatcatcatcatcatcTTCACAGCCATGATCATCCATGGACAAATGCAACTGAAGATCATTTTAGTTTTCATATGCTAATGATCAAATTCAGGTTAATTGCTGTTTAATTTCTCCATACACTAGTTGTTGTCTGCACCATTGCATTGTGCACAGCACACACACGCTTTTGATGCTTCTAGGAATGCATATCTGTTCAGCAGTTCACACAGTGCAGCAGGGCAATGTTGTTAAAAAATCTTCTCCTTTTTTTTATGTCCTTGTGTTCTTGAGCTTTCTGTCTCCATTGATCTGCTTTTTTCTTGTTTACAAGTGATGGGCACAAGTCACTTCCCTAGCTTCAGCTCATGCATGGAGCAGGAATCTCACTTCAAAAGACCTAGCACTTTTTCTCTCTTCACCTTTTTGCCTCAACACATGCCCAGTTTCTGGCCACACAAACATAAACACATATACTATCTAGCTGCATAATTGCATCAAATTAAGCAGGGTTTGTTTCAGCTAGGAATTCCACACATAGGTCATTAATTAGTATTGCCAACTTTCTCAACATGCATGCACTCTAGTACT

***OsSWEET13* sequence. PthXo2 EBE is highlighted in yellow. First exon is highlighted in grey.**

>OsSWEET13_Komboka_ 1444 bp

TAAAAGAACAATTAAATACGAGTACCCGTATCAAATTTAGGATTTAAATATATGTATGTATCATATATATTTCGAAATTAAGTATGCTTATATATGCAGATTGATTGCGTATATATATTGTGCTATGCTTATAGGTTGATTGGAAAGGCAAGCAGCCGCGCGCACAGAAAAAAGTAGAGAGAAAAAAAAAAGCAGATCCACTAGCTTAGCTTCATATACGTGGGAGTAGAACAAGATCAACGCGCTTCGCAGAAGCAGAAATCGACCTGTCTTCCCAACAAACTGTGTATGATAGCTTAGTCGACAGGGATGTCTACTGCAGGTGAAAACAATCCTTCGACAAAAAATAAGTTACTTTTGGTAAAGACAGTTAAATAATAAGCAGCTATATCACGCGCATGGGAGAATTGCATATTCAATTACAATCATTATTTTTTTTTCAGAACACTGTCGGCGACATTGAGAATTAATCTACCCGTGCAAACAAAGAACAGAGAAACTATAGTATACCTACATGTATCTATCACCCAATAATTGCAAGATCATGTTACAAAACGGTTCTAATTAATATATAGAAACAAGGCAGAGAATTCTACCTTTCTTTTGTCTAAGTACAATTATCTTTTTCTCCGCGATTAATATTTTTCGAGTAGTAAAATTTAAGTCAAAAGCCGTATCAGGATTCAGGAATAATCCTTCACTGGGAGAGATCTCATGTGATTTGCTGTTGCACTCGGCGGCTATCTTTTACCGTTCCCAGCAGGAAGCTGCAGACGTTGGAGAGATCGATCTCTACTGACAATGCACAAAGCAATTACTCACTAAATTGGCTATGGCTAGTGAGAGGTGCGCTGCGCACAAAGCCAATGCAACTTTTTTTGAAAATTAGCCAGGATTATCTCCAACAGTAGCTCATTTTTGTAAAAGCCTAATTATTGTGCGTGTCCAAAAGACTTTCCTCAAAAGCAAATAAAGAAAAAAAATCTTTGCATAATTATTCTATGATTACTTTGATGCGTACGTGAATGGCCATGGGTAGGAGGCAACCAAGTGATTCGCACCTAGCTAGCTTTTCTCCTATATAAGCACCACAACTCCCTTCATTCCTCTCCAAGAGTTTTCAGCCAACACATTGAACTCTTCTTCAGAGCTCTCCCTTCCCTCCACAAAGGGGTCTAGGGTTAGAGTGTGTGTGTCTGTGACAAGTTCCAAGCTAGCAACAACAAGCTCAATTCCTTGCTTGTTTGCTTCCATATTACACTACATCTCTTCCCTTCAATTACCCCCCTTTTAGCACACAAAAATGGCTGGCCTGTCCCTGCAGCATCCCTGGGCTTTTGCCTTCGGCCTCCTTGGTATATCATCATCACCTACCACAACTAAGACATTCCCTTCATTGCCAACATTTTACTTCTTTTTATTAGAAACCATTGAGTTTGTACAT

> *OsSWEET13*_Kitaake_OsativaKitaake_499.genome _Chr12 Chr12:17058815..17060261 (- strand) class=match length=1447 bp

TAAAAGAACAATTAAATACGAGTACCTGTATCAAATTTAGGATTTAAATATATGTATGTATCATATATATTTCGAAATTAAGTATGCTTATATATATGCAGATTGATTGCGTATATATATTGTGCTATGCTTATAGGTTGATTGGAAAGGCAAGCAGCCGCGCGCACAGAAAAAAGTAGAGAGAAAAAAAAAGCAGATCCACTAGCTTAGCTTCATATACGTGGGAGTAGAACAAGATCAACGCGCTTCGCAGAAGCAGAAATCGACCTGTCTTCCCAACAAACTGTGTATGAAAGCTTAGTCGACAGGGATGTCTACTGCAGGTGAAAACAATCCTTCGACAAAAAATAAGTTACTTTTGGTAAAGACAGTTAAATAATAAGCAGCTATATCACGCGCATGGGAGAATTGCATATTCAATTACAATCATTATTTTTTTTTCAGAACACTGTCGGCGACATTGAGAATTAATCTACCCGTGCAAACAAAGAACAGAGAAACTATAGTATACCTACATGTATCTATCACCCAATAATTGCAAGATCATGTTACAAAACGGTTCTAATTAATATATAGAAACAAGGCAGAGAATTCTACCTTTCTTTTGTCTAAGTACAATTATCTTTTTCTCCGCGATTAATATTTTTCGAGTAGTAAAATTTAAGTCAAAAGCCGTATCAGGATTCAGGAATAATCCTTCACTGGGAGAGATCTCATGTGATTTGCTGTTGCACTCGGCGGCTATCTTTTACCGTTCCCAGCAGGAAGCTGCAGACGTTGGAGAGATCGATCTCTACTGACAATGCACAAAGCAATTACTCACTAAATTGGCTATGGCTAGTGAGAGGTGCGCTGCGCACAAAGCCAATGCAACTTTTTTTGAAAATTAGCCAGGATTATCTCCAACAGTAGCTCATTTTTGTAAAAGCCTAATTATTGTGCGTGTCCAAAAGACTTTCCTCAAAAGCAAATAAAGAAAAAAAATCTTTGCATAATTATTCTATGATTACTTTGATGCGTACGTGAATGGCCATGGGTAGGAGGCAACCAAGTGATTCCCACCTAGCTAGCTTTGCTCCTATATAAAGCACCACAACTCCCTTCATTCCTCTCCAAGAGTTTTCAGCCAACACATTGAACTCTTCTTCAGAGCTCTCCCTTCCCTCCACAAAGGGGGTCTAGGGTTAGAGTGTGTGTGTCTGTGACAAGTTCCAAGCTAGCAACAACAAGCTCAATTCCTTGCTTGTTTGCTTCCATATTACACTACATCTCTTCCCTTCAATTACCCCCCTTTTAGCACACAAAAATGGCTGGCCTGTCCCTGCAGCATCCCTGGGCTTTTGCCTTCGGCCTCCTTGGTATATCATCATCACCTACCACAACTAAGACATTCCCTTCATTGCCAACATTTTACTTCTTTTTATTAGAAACCATTGAGTTTGTACAT

***OsSWEET14* sequence. TalC is highlighted in yellow. AvrXa7 EBE is colored in red. PthXo3 is highlighted in light turquoise. TalF EBE is underlined. First exon is highlighted in grey.**

>OsSWEET14_Komboka_ 888 bp

TGCGGCTCATCAGTTTCTCTAAGCTCTCACCATTCATTCCACTATACAAGCCTAAGGCAGCTAGCTTAGTTAATTACCTAATAACTATAGCTTGCCCAACTCTAGATCCCTTAACTAGGACAACTTGGAGTACACAACAATGTTAATAATCCCATGCATTGAGGACAGAGTTGTGAAGGAAACAAAAAAAAGCTAGCAGATTGGCACTTTCTGTCATGCATGGGTGCTGATGATTATCTTGTATCTAATTTAATCAATCCCATGGCTGTGATTGATCAGGAATAGTTTGTGTGTGCAGCTATATTACCTATTGGTGTCCAGGGTCACACACCATAAGGGCATGCATGTCAGCAGCTGGTCATGTGTGCCTTTTCATTCCCTTCTTCCTTCCTAGCACTATATAAACCCCCTCCAACCAGGTGCTAAGCTCATCAAGCCTTCAAGCAAAGCAAACTCAAGTAGTAGCTGATTACCAGCTCTTCTCTCTTCTCATTGAGAAGAGGGAATTAAGTTTTGATCTCTGCTTTATTGCCTGATCATCCTCTTGTTACTTGCAAGCAAGAACAGTAGTGTACTGTGCCTCATTGATCTCCTCTCACCAAACTCTCTCTCTCTCTCTCATATTCCGAGCTAGCTAGTTAATCAAGATCTTGCTGCAATGGCTGGCATGTCTCTTCAGCATCCCTGGGCCTTCGCCTTTGGTCTCCTAGGTGTGTTGCCTTTGATCTGATCCAAGGAATTCTCTTGAGAATTAATCTTGCATGGTTATTTACTTTTGTTGTTATTATTCTCTACATTTTTAATCATGTACTTTTCCATGTTCCTCTTTTGTTGCCAAAGCTACTATATTTTTCCTACCAATTCATCCAAAACTACTATATTATAGCA

> OsSWEET14_Kitaake_OsativaKitaake_499.genome Chr11 Chr11:18397214..18398101 (- strand) class=match length=888 bp

TGCGGCTCATCAGTTTCTCTAAGCTCTCACCATTCATTCCACTATACAAGCCTAAGGCAGCTAGCTTAGTTAATTACCTAATAACTATAGCTTGCCCAACTCTAGATCCCTTAACTAGGACAACTTGGAGTACACAACAATGTTACTAATCCCATGCATTGAGGACAGAGTTATGAAGGAAACAAAAAAAAGCTAGCAGATTGGCACTTTCTGTCATGCATGGGTGCTGATGATTATCTTGTATCTAATTTAATCAATCCCATGGCTGTGATTGATCAGGAATAGTTTGTGTGTGCAGCTATATTGCCTATTGGTGTCCAGGGTCACACACCATAAGGGCATGCATGTCAGCAGCTGGTCATGTGTGCCTTTTCATTCCCTTCTTCCTTCCTAGCACTATATAAACCCCCTCCAACCAGGTGCTAAGCTCATCAAGCCTTCAAGCAAAGCAAACTCAAGTAGTAGCTGATTACCAGCTCTTCTCTCTTCTCATTGAGAAGAGGGAATTAAGTTTTGATCTCTGCTTTATTGCCTGATCATCCTCTTGTTACTTGCAAGCAAGAACAGTAGTGTACTGTGCCTCATTGATCTCCTCCCACCAAACTCTCTCTCTCTCTCTCATATTCCGAGCTAGCTAGTTAATCAAGATCTTGCTGCAATGGCTGGCATGTCTCTTCAGCATCCCTGGGCCTTCGCCTTTGGTCTCCTAGGTGTGTTGCCTTTGATCTGATCCAAGGAATTCTCTTGAGAATTAATCTTGCATGGTTATTTACTTTTGTTGTTATTATTCTCTACATTTTTAATCATGTACTTTTCCATGTTCCACTTTTGTTGCCAAAGCTACTATATTTTTCCTACCAATTCATCCAAAACTACTATATTATAGCA

**Supplementary file 1f: Guide RNA sequences.** Green letters indicate the protospacer adjacent motif (PAM) sequence NGG.

| **Genome** | **gRNA/cRNA** | **Target locus** | **Sequence** |
| --- | --- | --- | --- |
| *Oryza sativa* japonica (Nipponbare, IRGSP1.0) | cXo1 | chr08: 26728846-26728872 | TTTG GTGGTGTACAGTAGGGGGAGATG |
| *Oryza sativa* indica (Shuhui498) | cXo1 | chr08: 28035804-28035830 | TTTG GTGGTGTACAGTAGGGGGAGATG |
| *Oryza sativa* japonica (Nipponbare, IRGSP1.0) | cXo2c | Komboka specific | TTTC TCCTATATAAGCACCACAACTCC |
| *Oryza sativa* indica (Shuhui498) | cXo2c | Komboka specific | TTTC TCCTATATAAGCACCACAACTCC |
| *Oryza sativa* japonica (Nipponbare, IRGSP1.0) | cXo2d | variety specific | TTTG TTCCTATATAAAGCACCACAACT |
| *Oryza sativa* indica (Shuhui498) | cXo2d | variety specific | TTTG TTCCTATATAAAGCACCACAACT |
| *Oryza sativa* japonica (Nipponbare, IRGSP1.0) | cTalF | chr11: 18174461-18174487 | TTTG CTTGAAGGCTTGATGAGCTTAGC |
| *Oryza sativa* indica (Shuhui498) | cTalF | chr11: 20518333-20518359 | TTTG CTTGAAGGCTTGATGAGCTTAGC |
| *Oryza sativa* japonica (Nipponbare, IRGSP1.0) | gTalC | chr11: 18174551-18174572 | GGGCATGCATGTCAGCAGC TGG |
| *Oryza sativa* indica (Shuhui498) | gTalC | chr11: 20518423-20518444 | GGGCATGCATGTCAGCAGC TGG |

**Supplementary file 1g. EBE sequences in select *SWEET* promoters of Komboka wild-type and CRISPR-edited lines.** DNA sequences shaded in gray are *SWEET* EBEs for respective TALes. Letters in blue: PAM sequence for LbCpf1; letters in pink: PAM sequence for SpCas9; letters in red: TATA boxes; bold lowercase letters: insertion mutations; dashed lines: deletions; Italic lowercase letters: substitution mutations. Lines in orange and bold font were subjected to pathogen tests. n.d., not determined.

| ***SWEET11*** |  | AGCAAAGGTTAGATATGCATCTCCCCCTACTGTACACCACCAAAAGTGGAGGGTCTCCAACTATATAAACAC (PthXo1 EBE is shaded) | Genotype |
| --- | --- | --- | --- |
|  | T0 generation | | |
| 1.1 to 1.7 | Biallelic | AGCAAAGGTTAGATATGC---------TACTGTACACCACCAAAAGTGGAGGGTCTCCAACTATATAAACAC  AGCAAAGGTTAGATATGC------------TGTACACCACCAAAAGTGGAGGGTCTCCAACTATATAAACAC | -9  -12 |
| 2 | Biallelic | AGCAAAGGTTAGATATG----------TACTGTACACCACCAAAAGTGGAGGGTCTCCAACTATATAAACAC  AGCAAAGGTTAGATA-------------------------//---GCTAGCAT | -10  -119 |
| 4 | Biallelic | AGCAAAGGTTAGATATGC---------TACTGTACACCACCAAAAGTGGAGGGTCTCCAACTATATAAACAC  AGCAAAGGTTA*a*ATATG**g**CAT----CCCTACTGTACACCACCAAAAGTGGAGGGTCTCCAACTATATAAACAC | -9  -4 |
| 7 | Biallelic | AGCAAAGGTTA*c*ATATG-------------TGTACACCACCAAAAGTGGAGGGTCTCCAACTATATAAACAC  AGCAAAGGTTA------------------CTGTACACCACCAAAAGTGGAGGGTCTCCAACTATATAAACAC | -13  -18 |
| **9** | Biallelic | AGCAAAGGTTAGATATGC---------TACTGTACACCACCAAAAGTGGAGGGTCTCCAACTATATAAACAC  AGCAAAGGTTAGATATGC------------TGTACACCACCAAAAGTGGAGGGTCTCCAACTATATAAACAC | -9  -12 |
| 10 | Biallelic | AGCAAAGGTTAGATAT-------------CTGTACACCACCAAAAGTGGAGGGTCTCCAACTATATAAACAC  AGCAAAGGTTAaATA--------------CTGTACACCACCAAAAGTGGAGGGTCTCCAACTATATAAACAC | -13  -14 |
| **12** | Biallelic | AGCAAAGGTTAGATATG----------TACTGTACACCACCAAAAGTGGAGGGTCTCCAACTATATAAACAC  AGCAAAGGTTAGATA-----------CTACTGTACACCACCAAAAGTGGAGGGTCTCCAACTATATAAACAC | -10  -11 |
| **14** | **Homo-zygous** | AGCAAAGGTTAGATATG---------CTACTGTACACCACCAAAAGTGGAGGGTCTCCAACTATATAAACAC | -9 |
| 16 | Biallelic | AGCAAAGGTTAGATATG------------CTGTACACCACCAAAAGTGGAGGGTCTCCAACTATATAAACAC  AGCAAAGGTTAGATA-----------CTACTGTACACCACCAAAAGTGGAGGGTCTCCAACTATATAAACAC | -12  -11 |
| **18** | Biallelic | AGCAAAGGTTAGATATGC--------CTACTGTACACCACCAAAAGTGGAGGGTCTCCAACTATATAAACAC  AGCAAAGGTTAGATATGC---------TACTGTACACCACCAAAAGTGGAGGGTCTCCAACTATATAAACAC | -8  -9 |
|  | T1 generation | | |
| 1.1_10 | Homo-zygous | AGCAAAGGTTAGATATG---------CTACTGTACACCACCAAAAGTGGAGGGTCTCCAACTATATAAACAC | -9 |
| 1.1_22 | Homo-zygous | AGCAAAGGTTA*G^*^*ATATG---------CTACTGTACACCACCAAAAGTGGAGGGTCTCCAACTATATAAACAC | -9;^*^G or C |
| 1.2_17 | Homo-zygous | AGCAAAGGTTAGATATG------------CTGTACACCACCAAAAGTGGAGGGTCTCCAACTATATAAACAC | -12 |
| 1.2_38 | Homo-zygous | AGCAAAGGTTAGATATG---------CTACTGTACACCACCAAAAGTGGAGGGTCTCCAACTATATAAACAC | -9 |
| **1.2_40** | Homo-zygous | AGCAAAGGTTAGATATG---------CTACTGTACACCACCAAAAGTGGAGGGTCTCCAACTATATAAACAC | -9 |
| **1.2_45** | Homo-zygous | AGCAAAGGTTAGATATG---------CTACTGTACACCACCAAAAGTGGAGGGTCTCCAACTATATAAACAC | -9 |
| 1.2_64 | Homo-zygous | AGCAAAGGTTAGATATG---------CTACTGTACACCACCAAAAGTGGAGGGTCTCCAACTATATAAACAC | -9 |
| 1.3_18 | Homo-zygous | AGCAAAGGTTAGATATG---------CTACTGTACACCACCAAAAGTGGAGGGTCTCCAACTATATAAACAC | -9 |
| 1.3_22 | Homo-zygous | AGCAAAGGTTAGATATG---------CTACTGTACACCACCAAAAGTGGAGGGTCTCCAACTATATAAACAC | -9 |
| **1.3_24** | Homo-zygous | AGCAAAGGTTAGATATG---------CTACTGTACACCACCAAAAGTGGAGGGTCTCCAACTATATAAACAC | -9 |
| 1.3_29 | Homo-zygous | AGCAAAGGTTAGATATG------------CTGTACACCACCAAAAGTGGAGGGTCTCCAACTATATAAACAC | -12 |
| 1.3_34 | Homo-zygous | AGCAAAGGTTAGATATG------------CTGTACACCACCAAAAGTGGAGGGTCTCCAACTATATAAACAC | -12 |
| 1.3_54 | Homo-zygous | AGCAAAGGTTAGATATG---------CTACTGTACACCACCAAAAGTGGAGGGTCTCCAACTATATAAACAC | -9 |
| 1.3_74 | Homo-zygous | AGCAAAGGTTAGATATG---------CTACTGTACACCACCAAAAGTGGAGGGTCTCCAACTATATAAACAC | -9 |
| 1.3_78 | Homo-zygous | AGCAAAGGTTAGATATG------------CTGTACACCACCAAAAGTGGAGGGTCTCCAACTATATAAACAC | -12 |
| 1.4_03 | Homo-zygous | AGCAAAGGTTAGATATG------------CTGTACACCACCAAAAGTGGAGGGTCTCCAACTATATAAACAC | -12 |
| 1.4_12 | Homo-zygous | AGCAAAGGTTAGATATG------------CTGTACACCACCAAAAGTGGAGGGTCTCCAACTATATAAACAC | -12 |
| 1.4_15 | Homo-zygous | AGCAAAGGTTAGATATG---------CTACTGTACACCACCAAAAGTGGAGGGTCTCCAACTATATAAACAC | -9 |
| **1.4_21** | Homo-zygous | AGCAAAGGTTAGATATG------------CTGTACACCACCAAAAGTGGAGGGTCTCCAACTATATAAACAC | -12 |
| **1.5_19** | Homo-zygous | AGCAAAGGTTAGATATG------------CTGTACACCACCAAAAGTGGAGGGTCTCCAACTATATAAACAC | -12 |
| 1.5_20 | Homo-zygous | AGCAAAGGTTAGATATG---------CTACTGTACACCACCAAAAGTGGAGGGTCTCCAACTATATAAACAC | -9 |
| 1.5_24 | Homo-zygous | AGCAAAGGTTAGATATG---------CTACTGTACACCACCAAAAGTGGAGGGTCTCCAACTATATAAACAC | -9 |
| **1.7_10** | Homo-zygous | AGCAAAGGTTAGATATG------------CTGTACACCACCAAAAGTGGAGGGTCTCCAACTATATAAACAC | -12 |
| 9_32 | Biallelic | AGCAAAGGTTAGATATGC---------TACTGTACACCACCAAAAGTGGAGGGTCTCCAACTATATAAACAC  AGCAAAGGTTAGATATGC------------TGTACACCACCAAAAGTGGAGGGTCTCCAACTATATAAACAC | -9 /-12 |
| 9_33 | Biallelic | AGCAAAGGTTAGATATGC---------TACTGTACACCACCAAAAGTGGAGGGTCTCCAACTATATAAACAC  AGCAAAGGTTAGATATGC------------TGTACACCACCAAAAGTGGAGGGTCTCCAACTATATAAACAC | -9 /-12 |
| 9_34 | Homo-zygous | AGCAAAGGTTAGATATGC------------TGTACACCACCAAAAGTGGAGGGTCTCCAACTATATAAACAC | -12 |
| 12_57 | Homo-zygous | AGCAAAGGTTAGATA-----------CTACTGTACACCACCAAAAGTGGAGGGTCTCCAACTATATAAACAC | -11 |
| 12_69 | Homo-zygous | AGCAAAGGTTAGATA-----------CTACTGTACACCACCAAAAGTGGAGGGTCTCCAACTATATAAACAC | -11 |
| 12_81 | Homo-zygous | AGCAAAGGTTAGATA-----------CTACTGTACACCACCAAAAGTGGAGGGTCTCCAACTATATAAACAC | -11 |
| **14_19** | Homo-zygous | AGCAAAGGTTAGATATG---------CTACTGTACACCACCAAAAGTGGAGGGTCTCCAACTATATAAACAC | -9 |
| 14_29 | Homo-zygous | AGCAAAGGTTAGATATG---------CTACTGTACACCACCAAAAGTGGAGGGTCTCCAACTATATAAACAC | -9 |
| 14_32 | Homo-zygous | AGCAAAGGTTAGATATG---------CTACTGTACACCACCAAAAGTGGAGGGTCTCCAACTATATAAACAC | -9 |
| **14_65** | Homo-zygous | AGCAAAGGTTAGATATG---------CTACTGTACACCACCAAAAGTGGAGGGTCTCCAACTATATAAACAC | -9 |
| 16_5 | Homo-zygous | AGCAAAGGTTAGATATG------------CTGTACACCACCAAAAGTGGAGGGTCTCCAACTATATAAACAC | -12 |
| 18_31 | Biallelic | AGCAAAGGTTAGATATGC--------CTACTGTACACCACCAAAAGTGGAGGGTCTCCAACTATATAAACAC  AGCAAAGGTTAGATATGC---------TACTGTACACCACCAAAAGTGGAGGGTCTCCAACTATATAAACAC | -8 /-9 |
| 18_68 | Homo-zygous | AGCAAAGGTTAGATATGC---------TACTGTACACCACCAAAAGTGGAGGGTCTCCAACTATATAAACAC | -9 |
| 18_118 | Biallelic | AGCAAAGGTTAGATATGC--------CTACTGTACACCACCAAAAGTGGAGGGTCTCCAACTATATAAACAC  AGCAAAGGTTAGATATGC---------TACTGTACACCACCAAAAGTGGAGGGTCTCCAACTATATAAACAC | -8 /-9 |
| 18_142 | Homo-zygous | AGCAAAGGTTAGATATGC---------TACTGTACACCACCAAAAGTGGAGGGTCTCCAACTATATAAACAC | -9 |
| 18_160 | Homo-zygous | AGCAAAGGTTAGATATGC--------CTACTGTACACCACCAAAAGTGGAGGGTCTCCAACTATATAAACAC | -8 |
| T2 generation |  | | |
| 1.2_45_25 | Homo-zygous | AGCAAAGGTTAGATATG----------TACTGTACACCACCAAAAGTGGAGGGTCTCCAACTATATAAACAC | -10 |
| 1.3_24_1 | Homo-zygous | AGCAAAGGTTAGATATG----------TACTGTACACCACCAAAAGTGGAGGGTCTCCAACTATATAAACAC | -10 |
| 1.3_24_29 | Homo-zygous | AGCAAAGGTTAGATATG---------CTACTGTACACCACCAAAAGTGGAGGGTCTCCAACTATATAAACAC | -9 |
|  |  |  |  |
| ***SWEET13*** |  | CTTTTCTCCTATATAAGCACCACAACTCCCTTCATTCCTCTCCAAGAGTTTTCAGCCAACACATTGAACT (PthXo2A EBE is shaded) |  |
|  | T0 generation | | |
| 1.1 | Biallelic | CTTTTCTCCTATATAAGCAC*t*-----------------CTCTCCAAGAGTTTTCAGCCAACACATTGAACT  CTTTTCTCCTATATAAGCACC-----------CATTCCTCTCCAAGAGTTTTCAGCCAACACATTGAACT | -17  -11 |
| 1.2 | Biallelic | CTTTTCTCCTATATAAGCAC---------CTTCATTCCTCTCCAAGAGTTTTCAGCCAACACATTGAACT  CTTTTCTCCTATATAAGCAC-----------TCATTCCTCTCCAAGAGTTTTCAGCCAACACATTGAACT | -9  -11 |
| 1.3 | Biallelic | CTTTTCTCCTATATAAGCACC---ACTCCCTTCATTCCTCTCCAAGAGTTTTCAGCCAACACATTGAACT  CTTTTCTCCTATATAAGCAC-----------TCATTCCTCTCCAAGAGTTTTCAGCCAACACATTGAACT | -3  -11 |
| 1.4 | Biallelic | CTTTTCTCCTATATAAGCAC--------------TTCCTCTCCAAGAGTTTTCAGCCAACACATTGAACT  CTTTTCTCCTATATAAGCAC-----------TCATTCCTCTCCAAGAGTTTTCAGCCAACACATTGAACT | -14  -11 |
| 1.5 | Biallelic | CTTTTCTCCTATATAAGCAC-------CCCTTCATTCCTCTCCAAGAGTTTTCAGCCAACACATTGAACT  CTTTTCTCCTATATAAGCAC*t*A-----------ATTCCTCTCCAAGAGTTTTCAGCCAACACATTGAACT | -7  -11 |
| 1.6 | Biallelic | CTTTTCTCCTATATAAGCACC*c*--ACTCCCTTCATTCCTCTCCAAGAGTTTTCAGCCAACACATTGAACT  CTTTTCTCCTATATAAGCAC-----------TCATTCCTCTCCAAGAGTTTTCAGCCAACACATTGAACT | -2  -11 |
| 1.7 | Biallelic | CTTTTCTCCTATATAAGCAC----------TTCATTCCTCTCCAAGAGTTTTCAGCCAACACATTGAACT  CTTTTCTCCTATATAAGCACC-----------CATTCCTCTCCAAGAGTTTTCAGCCAACACATTGAACT | -10  -11 |
| 2 | Biallelic | CTTTTCTCCTATATAAGCAC*a*-------CCTTCATTCCTCTCCAAGAGTTTTCAGCCAACACATTGAACT  CTTTTCTCCTATATAA-----------------------TCTCCAAGAGTTTTCAGCCAACACATTGAACT | -7  -23 |
| 4 | Biallelic | CTTTTCTCCTATATAAGCACC--------CTTCATTCCTCTCCAAGAGTTTTCAGCCAACACATTGAACT  CTTTTCTCCTATATAAGCACC-----TCCCTTCATTCCTCTCCAAGAGTTTTCAGCCAACACATTGAACT | -8  -5 |
| 7 | Biallelic | CTTTTCTCCTATATAAGCAC---------------TCCTCTCCAAGAGTTTTCAGCCAACACATTGAACT  CTTTTCTCCTAT--------------------CATTCCTCTCCAAGAGTTTTCAGCCAACACATTGAACT | -15  -20 |
| **9** | Biallelic | CTTTTCTCCTATATAA*c*GCACC---------TTCATTCCTCTCCAAGAGTTTTCAGCCAACACATTGAACT  CTTTTCTCCTA-----------------CCTTCATTCCTCTCCAAGAGTTTTCAGCCAACACATTGAACT | -9  -17 |
| 10 | Biallelic | CTTTTCTCCTATATAAGCAC------------//---AACACATTCCTCTC  CTTTTCTCCTATATAAGCACC--------CTTCATTCCTCTCCAAGAGTTTTCAGCCAACACATTGAACT | -37  -8 |
| **12** | Biallelic | CTTTTCTCCTATATAAGCACCA-----CCCTTCATTCCTCTCCAAGAGTTTTCAGCCAACACATTGAACT  CTTTTCTCCTATATAAGC-----------CTTCATTCCTCTCCAAGAGTTTTCAGCCAACACATTGAACT | -5b  -11 |
| **14** | Biallelic | CTTTTCTCCTATATAAGCACC----------TCATTCCTCTCCAAGAGTTTTCAGCCAACACATTGAACT  CTTTTCTCCTATATAAGCAC----------TTCATTCCTCTCCAAGAGTTTTCAGCCAACACATTGAACT | -10  -10 |
| 16 | Biallelic | CTTTTCTCCTATATAAGCAC*t*A---------TCATTCCTCTCCAAGAGTTTTCAGCCAACACATTGAACT  CTTTTCTCCTATATAAGCAC-------CCCTTCATTCCTCTCCAAGAGTTTTCAGCCAACACATTGAACT | -9  -7 |
| **18** | Biallelic | CTTTTCTCCTATATAAGCAC-----CTCCCTTCATTCCTCTCCAAGAGTTTTCAGCCAACACATTGAACT  TTCGCA--//------------------CCTTCATTCCTCTCCAAGAGTTTTCAGCCAACACATTGAACT | -5  -37 |
|  | T1 generation | | |
| 1.1_10 | Homo-zygous | CTTTTCTCCTATATAAGCAC-----------TCATTCCTCTCCAAGAGTTTTCAGCCAACACATTGAACT | -11 |
| 1.1_22 | Homo-zygous | CTTTTCTCCTATATAAGCA-----------------CCTCTCCAAGAGTTTTCAGCCAACACATTGAACT | -17 |
| 1.2_17 | Homo-zygous | CTTTTCTCCTATATAAGCA---------CCTTCATTCCTCTCCAAGAGTTTTCAGCCAACACATTGAACT | -9 |
| 1.2_38 | Homo-zygous | CTTTTCTCCTATATAAGCA---------CCTTCATTCCTCTCCAAGAGTTTTCAGCCAACACATTGAACT | -9 |
| **1.2_40** | Homo-zygous | CTTTTCTCCTATATAAGCAC-----------TCATTCCTCTCCAAGAGTTTTCAGCCAACACATTGAACT | -11 |
| **1.2_45** | Homo-zygous | CTTTTCTCCTATATAAGCA---------CCTTCATTCCTCTCCAAGAGTTTTCAGCCAACACATTGAACT | -9 |
| 1.2_64 | Homo-zygous | CTTTTCTCCTATATAAGCAC-----------TCATTCCTCTCCAAGAGTTTTCAGCCAACACATTGAACT | -11 |
| 1.3_18 | Homo-zygous | CTTTTCTCCTATATAAGCACC---ACTCCCTTCATTCCTCTCCAAGAGTTTTCAGCCAACACATTGAACT | -3 |
| 1.3_22 | Homo-zygous | CTTTTCTCCTATATAAGCACC---ACTCCCTTCATTCCTCTCCAAGAGTTTTCAGCCAACACATTGAACT | -3 |
| **1.3_24** | Homo-zygous | CTTTTCTCCTATATAAGCACC---ACTCCCTTCATTCCTCTCCAAGAGTTTTCAGCCAACACATTGAACT | -3 |
| 1.3_29 | Homo-zygous | CTTTTCTCCTATATAAGCAC-----------TCATTCCTCTCCAAGAGTTTTCAGCCAACACATTGAACT | -11 |
| 1.3_34 | Homo-zygous | CTTTTCTCCTATATAAG--------------TCATTCCTCTCCAAGAGTTTTCAGCCAACACATTGAACT | -14 |
| 1.3_54 | Homo-zygous | CTTTTCTCCTATATAAG--------------TCATTCCTCTCCAAGAGTTTTCAGCCAACACATTGAACT | -14 |
| 1.3_74 | Homo-zygous | CTTTTCTCCTATATAAGCAC-----------TCATTCCTCTCCAAGAGTTTTCAGCCAACACATTGAACT | -11 |
| 1.3_78 | Homo-zygous | CTTTTCTCCTATATAAG--------------TCATTCCTCTCCAAGAGTTTTCAGCCAACACATTGAACT | -14 |
| 1.4_03 | Homo-zygous | CTTTTCTCCTATATAAGCAC--------------TTCCTCTCCAAGAGTTTTCAGCCAACACATTGAACT | -14 |
| 1.4_12 | Homo-zygous | CTTTTCTCCTATATAAGCAC-----------TCATTCCTCTCCAAGAGTTTTCAGCCAACACATTGAACT | -11 |
| 1.4_15 | Homo-zygous | CTTTTCTCCTATATAAGCAC--------------TTCCTCTCCAAGAGTTTTCAGCCAACACATTGAACT | -14 |
| **1.4_21** | Homo-zygous | CTTTTCTCCTATATAAGCAC-----------TCATTCCTCTCCAAGAGTTTTCAGCCAACACATTGAACT | -11 |
| **1.5_19** | Homo-zygous | CTTTTCTCCTATATAAGCAC-----------TCATTCCTCTCCAAGAGTTTTCAGCCAACACATTGAACT | -11 |
| 1.5_20 | Homo-zygous | CTTTTCTCCTATATAAGCAC-----------TCATTCCTCTCCAAGAGTTTTCAGCCAACACATTGAACT | -11 |
| 1.5_24 | Homo-zygous | CTTTTCTCCTATATAAGCAC-----------TCATTCCTCTCCAAGAGTTTTCAGCCAACACATTGAACT | -11 |
| **1.7_10** | Homo-zygous | CTTTTCTCCTATATAAGCACC----------TCATTCCTCTCCAAGAGTTTTCAGCCAACACATTGAACT | -10 |
| 9_32 |  | n.d |  |
| 9_33 | Homo-zygous | CTTTTCTCCTA-----------------CCTTCATTCCTCTCCAAGAGTTTTCAGCCAACACATTGAACT | -17 |
| 9_34 | Homo-zygous | n.d. |  |
| 12_57 | Homo-zygous | CTTTTCTCCTATATAAGCACCA----CCCTTCATTCCTCTCCAAGAGTTTTCAGCCAACACATTGAACT | -5 |
| 12_69 | Homo-zygous | CTTTTCTCCTATATAAGCACCA----CCCTTCATTCCTCTCCAAGAGTTTTCAGCCAACACATTGAACT | -5 |
| 12_81 | Homo-zygous | CTTTTCTCCTATATAAG-----------CCTTCATTCCTCTCCAAGAGTTTTCAGCCAACACATTGAACT | -11 |
| **14_19** | Homo-zygous | CTTTTCTCCTATATAAGCA----------CTTCATTCCTCTCCAAGAGTTTTCAGCCAACACATTGAACT | -10 |
| 14_29 | Biallelic | CTTTTCTCCTATATAAGCA----------CTTCATTCCTCTCCAAGAGTTTTCAGCCAACACATTGAACT  CTTTTCTCCTATATAAGCA----------C*c*TCATTCCTCTCCAAGAGTTTTCAGCCAACACATTGAACT | -10 /-10 |
| 14_32 | Biallelic | CTTTTCTCCTATATAAGCA----------CTTCATTCCTCTCCAAGAGTTTTCAGCCAACACATTGAACT  CTTTTCTCCTATATAAGCA----------C*c*TCATTCCTCTCCAAGAGTTTTCAGCCAACACATTGAACT | -10/ -10 |
| **14_65** | Biallelic | CTTTTCTCCTATATAAGCA----------CTTCATTCCTCTCCAAGAGTTTTCAGCCAACACATTGAACT  CTTTTCTCCTATATAAGCA----------C*c*TCATTCCTCTCCAAGAGTTTTCAGCCAACACATTGAACT | -10/ -10 |
| 16_5 | Homo-zygous | CTTTTCTCCTATATAAGCACCA---------TCATTCCTCTCCAAGAGTTTTCAGCCAACACATTGAACT | -9 |
| 18_31 | Homo-zygous | CTTTTCTCCTATATAAGCAC-----CTCCCTTCATTCCTCTCCAAGAGTTTTCAGCCAACACATTGAACT | -5 |
| 18_68 | Homo-zygous | CTTTTCTCCTATATAAGCAC-----CTCCCTTCATTCCTCTCCAAGAGTTTTCAGCCAACACATTGAACT | -5 |
| 18_118 | Homo-zygous | CTTTTCTCCTATATAAGCAC-----CTCCCTTCATTCCTCTCCAAGAGTTTTCAGCCAACACATTGAACT | -5 |
| 18_142 | Homo-zygous | CTTTTCTCCTATATAAGCAC-----CTCCCTTCATTCCTCTCCAAGAGTTTTCAGCCAACACATTGAACT | -5 |
| 18_160 | Homo-zygous | CTTTTCTCCTATATAAGCAC-----CTCCCTTCATTCCTCTCCAAGAGTTTTCAGCCAACACATTGAACT | -5 |
|  | T2 generation | | |
| 1.2_45_25 | Homo-zygous | CTTTTCTCCTATATAAGCACCA---------TCATTCCTCTCCAAGAGTTTTCAGCCAACACATTGAACT | -9 |
| 1.3_24_1 | Homo-zygous | CTTTTCTCCTATATAAGCACCA---------TCATTCCTCTCCAAGAGTTTTCAGCCAACACATTGAACT | -9 |
| 1.3_24_29 | Homo-zygous | CTTTTCTCCTATATAAGCACCA---------TCATTCCTCTCCAAGAGTTTTCAGCCAACACATTGAACT | -9 |
|  |  | | |
| ***SWEET14*** |  | CATGCATGTCAGCAGCTGGTCATG...TATATAAACCCCCTCCAACCAGGTGCTAAGCTCATCAAGCCTTCAAGCAAA  (TalC, PthXo3, AvrXa7, TalF) | |
|  | T0 generation | | |
| 1.1 | Biallelic | CATGCATGT----AGCTGGTCATG...TATATAAACCCCCTCCAACCAGGTG-----------AAGCCTTCAA  CATGCA--------GCTGGTCATG...TAATAAACCCCCTCCAACCAGGTGC*a*----TCATCAAGCCTTCAA | -4/ -11  -8/ -4 |
| 1.2 | Biallelic | CATGCATGT----AGCTGGTCATG...TATATAAACCCCCTCCAACCAGGTG--------ATCAAGCCTTCAA  CATGCA--------GCTGGTCATG...TATATAAACCCCCTCCAACCAGGTGC-------ATCAAGCCTTCAA | -4/ -8  -8/ -7 |
| 1.3 | Biallelic | CATGCATGT----AGCTGGTCATG...TATATAAACCCCCTCCAACCAGGTG-----------AAGCCTTCAA  CATGCA--------GCTGGTCATG...TATATAAACCCCCTCCAACCAGGTGC*a*----TCATCAAGCCTTCAA | -4/ -11  -8/ -4 |
| 1.4 | Biallelic | CATGCATGT----AGCTGGTCATG...TATATAAACCCCCTCCAACCAGGTG--------ATCAAGCCTTCAA  CATGCA--------GCTGGTCATG...TATATAAACCCCCTCCAACCAGGTGC-------ATCAAGCCTTCAA | -4/ -8  -8/ -7 |
| 1.5, 1.6  and 1.7 | Biallelic | CATGCA-------AGCTGGTCATG...TATATAAACCCCCTCCAACCAGGTG--------ATCAAGCCTTCAA  CATGCA--------GCTGGTCATG...TATATAAACCCCCTCCAACCAGGT*t*--------ATCAAGCCTTCAA | -7/ -8  -8/ -8 |
| 2 | Biallelic | CATGCATGTCAGCA**a**GCTGGTCATG...TATATAAACCCCCTCCAACCAGG-------//--CAAAGCAACTA  CATGCA--------GCTGGTC*c*TG...TATATAAACCCCCTCCAACCAGGTG-------CATCAAGCCTTCAA | **+a**/ -24  -8/ -7 |
| 4 | Biallelic | CATGCATGTCAGC---TGGTCATG...TATATAAACCCCCTCCAACCAGGTGC--------------CTTCAA  CATGCATGTCAGC**c**AGCTGGTCATG...TATATAAACCCCCTCCAACCAGGTGCTA----CATCAAGC*g*TTCAA | -3/ -14  **+c**/ -4 |
| 7 | Biallelic | CATGCATGTCAGC---TGGTCATG...TATATAAACCCCCTCCAACCAGGTG--------ATCAAGCCTTCAA  CATGCAT------AGCTGGTCATG...TATATAAACCCCCTCCAACCAGGTGC---------CAAGCCTTCAA | -3/ -8  -6/ -9 |
| **9** | Biallelic | CATGCATGT---CAGCTGGTCATG...TATATAAACCCCCTCCAACCAGGTG-------CATCAAGCCTTCAA  CATGCATGTCAGCAGCTGGTCATG...TATATAAACCCCCTCCAA--------------------GCCTTCAA | -3/ -7  WT/ -20 |
| 10 | Biallelic | CATGCATGTCAGC---TGGT*a*ATG...TATATAAACCCCCTCCAACCAGGTGCTAAGCT--TCAAGCCTTCAA  CATGCA-------AGCTGGTCATG...TATATAAACCCCCTCCAACCAGGTGCTAA------TCAAGCC*a*TCAA | -3/ -2  -7/ -6 |
| **12** | Biallelic | CATGCAT-----CAGCTGGTCATG...TATATAAACCCCCTCCAACCAGGTG------TCATCAAGCCTTCAA  CATGCA*a*G-----AGCTGGTCATG...TATATAAACCCCCTCCAACCAGGTGCTAA-----TCAAGCCTTCAA | -5/ -6  -5/ -5 |
| **14** | Biallelic | CATGCA-------AGCTGGTCATG...TATATAAACCCCCTCCAACCAGGT-------TCATCAAGCCTTCAA  CATGCATGTCAGC**t**AGCTGGTCATG...TATATAAACCCCCTCCAACC---------------------TTCAA | -7/ -7  +**t**/ -21 |
| 16 | Biallelic | CATGCATGTCAGC---TGGTCATG...TATATAAACCCCCTCCAACCAGGTG-------CATCAAGCCTTCAA  CATGCAT------AGCTGGTCATG...TATATAAACCCCCTCCAACCAGGTGC------------GCCTTCAA | -3/ -7  -6/ -12 |
| **18** | Biallelic | CATGCATGTCAGCAG---GTCATG...TATATAAACCCCCTCCAACC-------------ATCAAGCCTTCAA  CATGCA--------GCTGGTC*t*TG...TATATAAACCCCCTCCAACCAGGTG------------//--TACTG | -3/ -13  -8/ -150 |
|  | T1 generation | | |
| 1.1_10 | Homo-zygous | CAT--------GCAGCTGGTCATG...TATATAAACCCCCTCCAACCAGGTGC-------ATCAAGCCTTCAA | -8/ -7 |
| 1.1_22 | Homo-zygous | CATGCATGT----AGCTGGTCATG...TATATAAACCCCCTCCAACCAGGTG--------ATCAAGCCTTCAA | -4/ -8 |
| 1.2_17 | Homo-zygous | CAT--------GCAGCTGGTCATG...TATATAAACCCCCTCCAACCAGGTGC-------ATCAAGCCTTCAA | -8/-7 |
| 1.2_38 | Homo-zygous | CATGCATGT----AGCTGGTCATG...TATATAAACCCCCTCCAACCAGGTG--------ATCAAGCCTTCAA | -4/ -8 |
| **1.2_40** | Homo-zygous | CAT--------GCAGCTGGTCATG...TATATAAACCCCCTCCAACCAGGTGC-------ATCAAGCCTTCAA | -8/ -7 |
| **1.2_45** | Homo-zygous | CAT--------GCAGCTGGTCATG...TATATAAACCCCCTCCAACCAGGTGC-------ATCAAGCCTTCAA | -8/ -7 |
| 1.2_64 | Homo-zygous | CATGCATGT----AGCTGGTCATG...TATATAAACCCCCTCCAACCAGGTG--------ATCAAGCCTTCAA | -4/ -8 |
| 1.3_18 | Homo-zygous | CATGCATGT----AGCTGGTCATG...TATATAAACCCCCTCCAACCAGGTG--------ATCAAGCCTTCAA | -4/ -8 |
| 1.3_22 | Homo-zygous | CATGCATGT----AGCTGGTCATG...TATATAAACCCCCTCCAACCAGGTG--------ATCAAGCCTTCAA | -4/ -8 |
| **1.3_24** | Homo-zygous | CAT--------GCAGCTGGTCATG...TATATAAACCCCCTCCAACCAGGTGC-------ATCAAGCCTTCAA | -8/ -7 |
| 1.3_29 | Homo-zygous | CATGCATGT----AGCTGGTCATG...TATATAAACCCCCTCCAACCAGGTG--------ATCAAGCCTTCAA | -4/ -8 |
| 1.3_34 | Homo-zygous | CATGCATGT----AGCTGGTCATG...TATATAAACCCCCTCCAACCAGGTG--------ATCAAGCCTTCAA | -4/ -8 |
| 1.3_54 | Homo-zygous | CAT--------GCAGCTGGTCATG...TATATAAACCCCCTCCAACCAGGTGC-------ATCAAGCCTTCAA | -8/ -7 |
| 1.3_74 | Homo-zygous | CATGCATGT----AGCTGGTCATG...TATATAAACCCCCTCCAACCAGGTG--------ATCAAGCCTTCAA | -4/ -8 |
| 1.3_78 | Homo-zygous | CAT--------GCAGCTGGTCATG...TATATAAACCCCCTCCAACCAGGTGC-------ATCAAGCCTTCAA | -8/ -7 |
| 1.4_03 | Homo-zygous | CATGCATGT----AGCTGGTCATG...TATATAAACCCCCTCCAACCAGGTG--------ATCAAGCCTTCAA | -4/ -8 |
| 1.4_12 | Homo-zygous | CAT--------GCAGCTGGTCATG...TATATAAACCCCCTCCAACCAGGTGC-------ATCAAGCCTTCAA | -8/ -7 |
| 1.4_15 | Homo-zygous | CATGCATGT----AGCTGGTCATG...TATATAAACCCCCTCCAACCAGGTG--------ATCAAGCCTTCAA | -4/ -8 |
| **1.4_21** | Homo-zygous | CATGCATGT----AGCTGGTCATG...TATATAAACCCCCTCCAACCAGGTG--------ATCAAGCCTTCAA | -4/ -8 |
| **1.5_19** | Homo-zygous | CAT--------GCAGCTGGTCATG...TATATAAACCCCCTCCAACCAGGT*t*--------ATCAAGCCTTCAA | -8/ -8 |
| 1.5_20 | Homo-zygous | CAT--------GCAGCTGGTCATG...TATATAAACCCCCTCCAACCAGGT*t*--------ATCAAGCCTTCAA | -8/ -8 |
| 1.5_24 | Homo-zygous | CATGCA-------AGCTGGTCATG...TATATAAACCCCCTCCAACCAGGTG--------ATCAAGCCTTCAA | -7/ -8 |
| **1.7_10** | Homo-zygous | CATGCA-------AGCTGGTCATG...TATATAAACCCCCTCCAACCAGGTG--------ATCAAGCCTTCAA | -7/ -8 |
| 9_32 | Homo-zygous | CATGCATGT---CAGCTGGTCATG...TATATAAACCCCCTCCAACCAGGTG-------CATCAAGCCTTCAA | -3/ -7 |
| 9_33 | Homo-zygous | CATGCATGTCAGCAGCTGGTCATG...TATATAAACCCCCTCCAACCAGGTGCTAAGCTCATCAAGCCTTCAA | WT |
| 9_34 | Homo-zygous | CATGCATGT---CAGCTGGTCATG...TATATAAACCCCCTCCAACCAGGTG-------CATCAAGCCTTCAA | -3 /-7 |
| 12_57 | Homo-zygous | CATGCATG-----AGCTGGTCATG...TATATAAACCCCCTCCAACCAGGTGCTAA-----TCAAGCCTTCAA | -5/ -5 |
| 12_69 | Homo-zygous | CATGCATG-----AGCTGGTCATG...TATATAAACCCCCTCCAACCAGGTGCTAA-----TCAAGCCTTCAA | -5/ -5 |
| 12_81 | Homo-zygous | CATGCATG-----AGCTGGTCATG...TATATAAACCCCCTCCAACCAGGTGCTAA-----TCAAGCCTTCAA | -5/ -5 |
| **14_19** | Homo-zygous | CATGCATGTCAGC**t**GCTGGTCATG...TATATAAACCCCCTCCAACC---------------------TTCAA | +**t**/ -21 |
| 14_29 | Homo-zygous | CATGCATGTCAGC**t**GCTGGTCATG...TATATAAACCCCCTCCAACC---------------------TTCAA | +**t**/ -21 |
| 14_32 | Homo-zygous | CATGCATGTCAGC**t**GCTGGTCATG...TATATAAACCCCCTCCAACC---------------------TTCAA | +**t**/ -21 |
| **14_65** | Homo-zygous | CATGCA-------AGCTGGTCATG...TATATAAACCCCCTCCAACCAGGT-------TCATCAAGCCTTCAA | -7/ -7 |
| 16_5 | Homo-zygous | CATGCAT------AGCTGGTCATG...TATATAAACCCCCTCCAACCAGGTGC------------GCCTTCAA | -6/ -12 |
| 18_31 | Homo-zygous | CATGCATGTCAGCAG---GTCATG...TATATAAACCCCCTCCAACC-------------ATCAAGCCTTCAA | -3/ -13 |
| 18_68 | Homo-zygous | CATGCATGTCAGCAG---GTCATG...TATATAAACCCCCTCCAACC-------------ATCAAGCCTTCAA | -3/ -13 |
| 18_118 | Homo-zygous | CATGCATGTCAGCAG---GTCATG...TATATAAACCCCCTCCAACC-------------ATCAAGCCTTCAA | -3/ -13 |
| 18_142 | Homo-zygous | CATGCATGTCAGCAG---GTCATG...TATATAAACCCCCTCCAACC-------------ATCAAGCCTTCAA | -3/ -13 |
| 18_160 | Homo-zygous | CATGCATGTCAGCAG---GTCATG...TATATAAACCCCCTCCAACC-------------ATCAAGCCTTCAA | -3/ -13 |
|  | T2 generation | | |
| 1.2_45_25 | Homo-zygous | CAT--------GCAGCTGGTCATG...TATATAAACCCCCTCCAACCAGGTGC-------ATCAAGCCTTCAA | -8/ -7 |
| 1.3_24_1 | Homo-zygous | CAT--------GCA*t*CTGGTCATG...TATATAAACCCCCTCCAACCAGGTGC-------ATCAAGCCTTCAA | -8/ -7 |
| 1.3_24_29 | Homo-zygous | CAT--------GCAGCTGGTCATG...TATATAAACCCCCTCCAACCAGGTGC-------ATCAAGCCTTCAA | -8/ -7 |

**Supplementary file 1h. List of SNPs and Indels in SWEET promoters after editing of the rice varieties of Komboka and Kitaake**

| Gene | Position before start codon* | Komboka | Kitaake |
| --- | --- | --- | --- |
| *SWEET11a* | 816 | +AA |  |
| *SWEET11a* | 857 | +T |  |
| *SWEET11a* | 888 | +TT |  |
| *SWEET13* | 132 | -G |  |
| *SWEET13* | 221 | -A (at PthXo2 EBE) |  |
| *SWEET13* | 232 | T | G |
| *SWEET13* | 248 | G | C |
| *SWEET13* | 1013 | T | A |
| *SWEET13* | 1122 | +A |  |
| *SWEET13* | 1216 | -TA |  |
| *SWEET13* | 1280 | C | T |
| *SWEET14* | 63 | T | C |
| *SWEET14* | 353 | A | G |
| *SWEET14* | 486 | G | A |
| *SWEET14* | 513 | A | C |

* for details see Supplementary Table 1g

**Supplementary file 1i. List of T0 lines with biallelic mutations at all targeted EBEs.** Same mutation types are highlighted with the same colors. Asterisks indicate mutations outside the EBE. For example: G/A*, +G, -4bp means one substitution of G to A outside of the EBE, one G insertion and a 4-bp deletion within the EBE.

| **Trans-forma-**  **tion Experiment** | **T0 Event** | **PthXo1** | | **PthXo2** | | **TalC** | | **PthXo3,**  **AvrXa7, TalF** | |
| --- | --- | --- | --- | --- | --- | --- | --- | --- | --- |
|  |  | **Allele 1** | **Allele 2** | **Allele 1** | **Allele 2** | **Allele 1** | **Allele 2** | **Allele 1** | **Allele 2** |
| 1 | 1.1 | -9bp | -12bp | C/T,  -17bp* | -11bp | -4bp | -8bp | -11bp | T/A,  -4bp |
|  | 1.2 | -9bp | -12bp | -9bp | -11bp | -4bp | -8bp | -8bp | -7bp |
|  | 1.3 | -9bp | -12bp | -3bp | -11bp | -4bp | -8bp | -11bp | T/A,  -4bp |
|  | 1.4 | -9bp | -12bp | -14bp* | -11bp | -4bp | -8bp | -8bp | -7bp |
|  | 1.5 | -9bp | -12bp | -7bp | C/T,  -11bp* | -7bp | -8bp | -8bp | G/T,  -8bp |
|  | 1.6 | -9bp | -12bp | A/C,  -2bp | -11bp | -7bp | -8bp | -8bp | G/T,  -8bp |
|  | 1.7 | -9bp | -12bp | -10bp | -11bp | -7bp | -8bp | -8bp | G/T,  -8bp |
| 2 | 2 | -10bp | -119bp* | C/A,  -7bp | -23bp | +A | -8bp, A/C | -24bp | -7bp |
|  | 4 | -9bp | G/A*, +G, -4bp | -8bp | -5bp | -3bp | +C | -14bp | -4bp, C/G |
|  | 7 | G/C*,  -13bp | -18bp | -15bp | -20bp | -3bp | -6bp | -9bp | -8bp |
|  | 9 | -9bp | -12bp | A/C,  -9bp | -17bp | -3bp | WT | -7bp | -20bp |
|  | 10 | -13bp | G/A*,  -14bp | -37bp | -8bp | -3bp, C/A | -7bp | -2bp | -6bp, T/A |
|  | 12 | -10bp | -11bp | -5bp | -11bp | -5bp | T/A,  -5bp | -6bp | -5bp |
|  | 14 | -9bp (homo-allelic) | | -10bp | -10bp | -7bp | +T | -7bp | -21bp |
|  | 16 | -12bp | -11bp | C/T,  -9bp | -7bp | -3pb | -6pb | -7bp | -12bp |
|  | 18 | -8bp | -9bp | -5bp | -37bp | -3bp | -8bp, A/T | -13bp | -150bp* |
|  | Total | 15 variants | | 27 variants | | 16 variants | | 21 variants | |

**Supplementary file 1j. Screening of the T1 generation for homozygous mutations in all six SWEET EBEs.**

|  | **T0 event** | **# of plants screened** | **Homozygous mutations**  **in all six EBEs** |
| --- | --- | --- | --- |
| Transformation  Round 1 | 1.1 | 14 | 2 |
|  | 1.2 | 52 | 5 |
|  | 1.3 | 87 | 8 |
|  | 1.4 | 21 | 4 |
|  | 1.5 | 26 | 3 |
|  | 1.6 | 21 | 0 |
|  | 1.7 | 12 | 1 |
|  | **Total for Round 1** | **233** | **23** |
| Transformation  Round 2 | 12 | 26 | 3 |
|  | 14 | 10 | 4 |
|  | 16 | 16 | 0 |
|  | **Total for Round 2** | **52** | **7** |
| **Sum** |  | **285** | **30** |

**Supplementary file 1k. List of T1 lines with homozygous mutations of all targeted EBEs.** n.d. not determined.

| **EBE / Line ID** | **SWEET11 PthXo1** | **SWEET13 PthXo2** | **SWEET14 TalC** | **SWEET14 PthXo3** | **SWEET14 AvrXa7** | **SWEET14 TalF** | **SpCas9 gene copy/2n** |
| --- | --- | --- | --- | --- | --- | --- | --- |
| 1.1_10 | -9bp | -11bp | -8bp | -5bp | -4bp | -5bp | 7 |
| 1.1_22 | -9bp | -17bp | -4bp | -5bp | -4bp | -6bp | 3 |
| 1.2_17 | -12bp | -9bp | -8bp | -5bp | -4bp | -5bp | 5 |
| 1.2_38 | -9bp | -9bp | -4bp | -5bp | -4bp | -6bp | 7 |
| 1.2_40 | -9bp | -11bp | -8bp | -5bp | -4bp | -5bp | 4 |
| 1.2_45 | -9bp | -9bp | -8bp | -5bp | -4bp | -5bp | 4 |
| 1.2_64 | -9bp | -11bp | -4bp | -5bp | -4bp | -6bp | 4 |
| 1.3_18 | -9bp | -3bp | -4bp | -5bp | -4bp | -6bp | 3 |
| 1.3_22 | -9bp | -3bp | -4bp | -5bp | -4bp | -6bp | 1 |
| 1.3_24 | -9bp | -3bp | -8bp | -5bp | -4bp | -5bp | 2 |
| 1.3_29 | -12bp | -11bp | -4bp | -5bp | -4bp | -6bp | 4 |
| 1.3_34 | -12bp | -14bp | -4bp | -5bp | -4bp | -6bp | 4 |
| 1.3_54 | -9bp | -14bp | -8bp | -5bp | -4bp | -5bp | 6 |
| 1.3_74 | -9bp | -11bp | -4bp | -5bp | -4bp | -6bp | 3 |
| 1.3_78 | -12bp | -14bp | -8bp | -5bp | -4bp | -5bp | 7 |
| 1.4_03 | -12bp | -14bp | -4bp | -5bp | -4bp | -6bp | 6 |
| 1.4_12 | -12bp | -11bp | -8bp | -5bp | -4bp | -5bp | 4 |
| 1.4_15 | -9bp | -14bp | -4bp | -5bp | -4bp | -6bp | 4 |
| 1.4_21 | -12bp | -11bp | -4bp | -5bp | -4bp | -6bp | 4 |
| 1.5_19 | -12bp | -11bp | -8bp | -7bp; G/T | G/T, -4bp | -5bp | 4 |
| 1.5_20 | -9bp | -11bp | -8bp | 7bp; G/T | G/T, -4bp | -5bp | 6 |
| 1.5_24 | -9bp | -11bp | -7bp | -5bp | -4bp | -6bp | 6 |
| 1.7_10 | -12bp | -10bp | -7bp | -5bp | -4bp | -6bp | 4 |
| 12_57 | -11bp | -5bp | -5bp | -2bp | -1bp | -4bp | 0 |
| 12_69 | -11bp | -5bp | -5bp | -2bp | -1bp | -4bp | 0 |
| 12_81 | -11bp | -11bp | -5bp | -2bp | -1bp | -4bp | 0 |
| 14_19 | -9bp | -10bp | +T | -12bp | -11bp | -12bp | 0 |
| 14_29 | -9bp | -10bp/  -10bp | +T | -12bp | -11bp | -12bp | 0 |
| 14_32 | -9bp | -10bp/  -10bp | +T | -12bp | -11bp | -12bp | 0 |
| 14_65 | -9bp | -10bp/  -10bp | -7bp | -6bp | -5bp | -4bp | 2 |
| 16_5 | -12bp | -9bp | -6bp | -4bp | -3bp | -11bp | n.d. |

**Supplementary file 1m. Alignment of the promoters of OsSWEET11a from the rice cv. Komboka and Kitaake**

>jgi:Chr8_499 OsativaKitaake|499|v3.1

Length=29331338

Score = 3090 bits (3426), Expect = 0.0

Identities = 1747/1762 (99%), Gaps = 13/1762 (1%)

Strand=Plus/Minus

Query 1 CTCTCTAAGAATAGGCATATTATGGTTTAATTAGCTAGACAAGAAATTAGTTAAGGTTCT 60

||||||||||||||||||||||||||||||||||||||||||||||||||||||||||||

Sbjct 27604137 CTCTCTAAGAATAGGCATATTATGGTTTAATTAGCTAGACAAGAAATTAGTTAAGGTTCT 27604078

Query 61 TAATGAAGATTCCGTCACTTTTGCTAGCTTTGAAAACCTGCAGAGTGAATTGTGCAAAAC 120

||||||||||||||||||||||||||||||||||||||||||||||||||||||||||||

Sbjct 27604077 TAATGAAGATTCCGTCACTTTTGCTAGCTTTGAAAACCTGCAGAGTGAATTGTGCAAAAC 27604018

Query 121 ATCTTGGCATGTTGGTGTTAGTGGTACAACAGATGCTGATACAAAGAAATTAATGCTGCA 180

||||||||||||||||||||||||||||||||||||||||||||||||||||||||||||

Sbjct 27604017 ATCTTGGCATGTTGGTGTTAGTGGTACAACAGATGCTGATACAAAGAAATTAATGCTGCA 27603958

Query 181 ATTGTTAGAAGCTCttttttttttCTCTCTTTTGAACACCAAAGCACTTTTGTTCATGGT 240

|||||||||||||||||||||| |||||||||||||||||||||||||| |||||||||

Sbjct 27603957 ATTGTTAGAAGCTCTTTTTTTT--CTCTCTTTTGAACACCAAAGCACTTT-GTTCATGGT 27603901

Query 241 GAAAGGGACTACCCTTCTCTTTCATATGTTCCCTTCTTCCTCTGTTCTTTTCATGAGATT 300

||||||||||||||||||||||||||||||||||||||||||||||||||||||||||||

Sbjct 27603900 GAAAGGGACTACCCTTCTCTTTCATATGTTCCCTTCTTCCTCTGTTCTTTTCATGAGATT 27603841

Query 301 TATTTGATGACTTCCCTCTGCTCTGTTATTTTCATGTTCTTTGATAAATGTGCTGATTGT 360

||||||||||||||||||||||||||||||||||||||||||||||||||||||||||||

Sbjct 27603840 TATTTGATGACTTCCCTCTGCTCTGTTATTTTCATGTTCTTTGATAAATGTGCTGATTGT 27603781

Query 361 ATTATCAGTGTTATTGCACTCAATTCATTCTTGAACAGTGTGTGAATTAACTCTTTGTTC 420

||||||||||||||||||||||||||||||||||||||||||||||||||||||||||||

Sbjct 27603780 ATTATCAGTGTTATTGCACTCAATTCATTCTTGAACAGTGTGTGAATTAACTCTTTGTTC 27603721

Query 421 GATTTGAGCTTAGTCACTTGATTGCACACGAACTACTCTGCAATTCTTTTCTGACGATAG 480

||||||||||||||||||||||||||||||||||||||||||||||||||||||||||||

Sbjct 27603720 GATTTGAGCTTAGTCACTTGATTGCACACGAACTACTCTGCAATTCTTTTCTGACGATAG 27603661

Query 481 AAGTCGATTGATGGATCACCCAACATGTTACTAATTAAGTTGCATCATTGTCCATGGTTG 540

||||||||||||||||||||||||||||||||||||||||||||||||||||||||||||

Sbjct 27603660 AAGTCGATTGATGGATCACCCAACATGTTACTAATTAAGTTGCATCATTGTCCATGGTTG 27603601

Query 541 TACATCCTTCTACAAATAAAACTACACAAATCAAGAAAATTTTCAATAACATTTCAACTA 600

||||||||||||||||||||||||||||||||||||||||||||||||||||||||||||

Sbjct 27603600 TACATCCTTCTACAAATAAAACTACACAAATCAAGAAAATTTTCAATAACATTTCAACTA 27603541

Query 601 TTGTAACAAGTAAAAGAAACCTATATGAGAGCTCCAGCTCTCCAAATGGCAACAGACACA 660

||||||||||||||||||||||||||||||||||||||||||||||||||||||||||||

Sbjct 27603540 TTGTAACAAGTAAAAGAAACCTATATGAGAGCTCCAGCTCTCCAAATGGCAACAGACACA 27603481

Query 661 CTGAGTGGTCATACGTGTCATATTGCCCCTCAGTTATGCATTCATATGACCACATATTCA 720

||||||||||||||||||||||||||||||||||||||||||||||||||||||||||||

Sbjct 27603480 CTGAGTGGTCATACGTGTCATATTGCCCCTCAGTTATGCATTCATATGACCACATATTCA 27603421

Query 721 GAGTAGTGGAGAGAGGGACAGATCTAGAGGTagaaaaagaaaattcatataaatgatata 780

||||||||||||||||||||||||||||||||||||||||||||||||||||||||||||

Sbjct 27603420 GAGTAGTGGAGAGAGGGACAGATCTAGAGGTAGAAAAAGAAAATTCATATAAATGATATA 27603361

Query 781 tcagagtgaaaaagaaatatcaagcacaagaaaaaaaaaaGCAAAGGTTAGATATGCATC 840

|||||||||||||||||||||||||||||| ||||||||||||||||||||||||||||

Sbjct 27603360 TCAGAGTGAAAAAGAAATATCAAGCACAAG--AAAAAAAAGCAAAGGTTAGATATGCATC 27603303

Query 841 TCCCCCTACTGTACACCACCAAAAGTGGAGGGTCTCCAACTATATAAACACTGAGCCATG 900

||||||||||||||||||||||||||||||||||||||||||||||||||||||||||||

Sbjct 27603302 TCCCCCTACTGTACACCACCAAAAGTGGAGGGTCTCCAACTATATAAACACTGAGCCATG 27603243

Query 901 GCCAAGGCCAAACCACACATGCAGTTGTAGTAGCACTTAAGCCTTCCTCTCTAGCTAGCA 960

||||||||||||||||||||||||||||||||||||||||||||||||||||||||||||

Sbjct 27603242 GCCAAGGCCAAACCACACATGCAGTTGTAGTAGCACTTAAGCCTTCCTCTCTAGCTAGCA 27603183

Query 961 TCTCTTGTGTCAGGAAGTTGGAAGGGATTTCTGGCTAGTTTCTAGCTGGTGTCTCCTCTC 1020

||||||||||||||||||||||||||||||||||||||||||||||||||||||||||||

Sbjct 27603182 TCTCTTGTGTCAGGAAGTTGGAAGGGATTTCTGGCTAGTTTCTAGCTGGTGTCTCCTCTC 27603123

Query 1021 CTCTTCCTAACCTTCTCACTGATTAACACCTTAGAGTTAGTTAATAACCTTCATCACCAG 1080

||||||||||||||||||||||||||||||||||||||||||||||||||||||||||||

Sbjct 27603122 CTCTTCCTAACCTTCTCACTGATTAACACCTTAGAGTTAGTTAATAACCTTCATCACCAG 27603063

Query 1081 TAGCAATGGCAGGAGGTTTCTTGTCCATGGCTAACCCGGCGGTCACCCTCTCCGGTGTTG 1140

||||||||||||||||||||||||||||||||||||||||||||||||||||||||||||

Sbjct 27603062 TAGCAATGGCAGGAGGTTTCTTGTCCATGGCTAACCCGGCGGTCACCCTCTCCGGTGTTG 27603003

Query 1141 CAGGTAAAGCATGCAACCAATGCATAATGCTCAAACTTAATT---tcatcattatcatca 1197

|||||||||||||||||||||||||||||||||||||||||| ||||||| |||||||

Sbjct 27603002 CAGGTAAAGCATGCAACCAATGCATAATGCTCAAACTTAATTTCATCATCATCATCATCA 27602943

Query 1198 tcatcatcttcacagccatgatcatccatGGACAAATGCAACTGAAGATCATTTTAGTTT 1257

||||||||||||||||||||||||||||||||||||||||||||||||||||||||||||

Sbjct 27602942 TCATCATCTTCACAGCCATGATCATCCATGGACAAATGCAACTGAAGATCATTTTAGTTT 27602883

Query 1258 TCATATGCTAATGATCAAATTCAGGTTAATTGCTGTTTAATTTCTCCATACACTA---GT 1314

||||||||||||||||||||||||||||||||||||||||||||||||||||||| ||

Sbjct 27602882 TCATATGCTAATGATCAAATTCAGGTTAATTGCTGTTTAATTTCTCCATACACTAGTTGT 27602823

Query 1315 TGTCTGCACCATTGCATTGTGCACAGCACACACACGCTTTTGATGCTTCTAGGAATGCAT 1374

||||||||||||||||||||||||||||||||||||||||||||||||||||||||||||

Sbjct 27602822 TGTCTGCACCATTGCATTGTGCACAGCACACACACGCTTTTGATGCTTCTAGGAATGCAT 27602763

Query 1375 ATCTGTTCAGCAGTTCACACAGTGCAGCAGGGCAATGTTGTTAAAAAATCTTCTCCtttt 1434

|||||||||||||||||||||||||||||||||||||||||||||||||||||||| |||

Sbjct 27602762 ATCTGTTCAGCAGTTCACACAGTGCAGCAGGGCAATGTTGTTAAAAAATCTTCTCC-TTT 27602704

Query 1435 tttttATGTCCTTGTATTCTTGAGCTTTCTGTCTCCATTGATCTGCTTTTTTCTTGTTTA 1494

||||||||||||||| ||||||||||||||||||||||||||||||||||||||||||||

Sbjct 27602703 TTTTTATGTCCTTGTGTTCTTGAGCTTTCTGTCTCCATTGATCTGCTTTTTTCTTGTTTA 27602644

Query 1495 CAAGTGATGGGCACAAGTCACTTCCCTTAGCTTCAGCTCATGCATGGAGCAGGAATCTCA 1554

|||||||||||||||||||||||||| |||||||||||||||||||||||||||||||||

Sbjct 27602643 CAAGTGATGGGCACAAGTCACTTCCC-TAGCTTCAGCTCATGCATGGAGCAGGAATCTCA 27602585

Query 1555 CTTCAAAAGACCTAGCACTTTTTCTCTCTTCACCTTTTTGCCTCAACACATGCCCAGTTT 1614

||||||||||||||||||||||||||||||||||||||||||||||||||||||||||||

Sbjct 27602584 CTTCAAAAGACCTAGCACTTTTTCTCTCTTCACCTTTTTGCCTCAACACATGCCCAGTTT 27602525

Query 1615 CTGGCCACACAAACATAAACACATATACTATCTAGCTGCATAATTGCATCAAATTAAGCA 1674

||||||||||||||||||||||||||||||||||||||||||||||||||||||||||||

Sbjct 27602524 CTGGCCACACAAACATAAACACATATACTATCTAGCTGCATAATTGCATCAAATTAAGCA 27602465

Query 1675 GGGTTTGTTTCAGCTAGGAATTCCACACATAGGTCATTAATTAGTATTGCCAACTTTCTC 1734

||||||||||||||||||||||||||||||||||||||||||||||||||||||||||||

Sbjct 27602464 GGGTTTGTTTCAGCTAGGAATTCCACACATAGGTCATTAATTAGTATTGCCAACTTTCTC 27602405

Query 1735 AACATGCATGCACTCTAGTACT 1756

||||||||||||||||||||||

Sbjct 27602404 AACATGCATGCACTCTAGTACT 27602383

**Alignment of OsSWEET13_1444bp between Komboka and Kitaake**

>jgi:Chr12_499 OsativaKitaake|499|v3.1

Length=27153574

Score = 2558 bits (2836), Expect = 0.0

Identities = 1439/1448 (99%), Gaps = 5/1448 (0%)

Strand=Plus/Minus

Query 1 TAAAAGAACAATTAAATACGAGTACCCGTATCAAATTTAGGATTTAAATATATGtatgta 60

|||||||||||||||||||||||||| |||||||||||||||||||||||||||||||||

Sbjct 17060261 TAAAAGAACAATTAAATACGAGTACCTGTATCAAATTTAGGATTTAAATATATGTATGTA 17060202

Query 61 tcatatatatttcgaaattaagtatgcttatatat--gcagattgattgcgtatatatat 118

||||||||||||||||||||||||||||||||||| |||||||||||||||||||||||

Sbjct 17060201 TCATATATATTTCGAAATTAAGTATGCTTATATATATGCAGATTGATTGCGTATATATAT 17060142

Query 119 TGTGCTATGCTTATAGGTTGATTGGAAAGGCAAGCAGCCGCGCGCACAGAAAAAAGTAGA 178

||||||||||||||||||||||||||||||||||||||||||||||||||||||||||||

Sbjct 17060141 TGTGCTATGCTTATAGGTTGATTGGAAAGGCAAGCAGCCGCGCGCACAGAAAAAAGTAGA 17060082

Query 179 GAGaaaaaaaaaaGCAGATCCACTAGCTTAGCTTCATATACGTGGGAGTAGAACAAGATC 238

|||||||||||| |||||||||||||||||||||||||||||||||||||||||||||||

Sbjct 17060081 GAGAAAAAAAAA-GCAGATCCACTAGCTTAGCTTCATATACGTGGGAGTAGAACAAGATC 17060023

Query 239 AACGCGCTTCGCAGAAGCAGAAATCGACCTGTCTTCCCAACAAACTGTGTATGATAGCTT 298

|||||||||||||||||||||||||||||||||||||||||||||||||||||| |||||

Sbjct 17060022 AACGCGCTTCGCAGAAGCAGAAATCGACCTGTCTTCCCAACAAACTGTGTATGAAAGCTT 17059963

Query 299 AGTCGACAGGGATGTCTACTGCAGGTGAAAACAATCCTTCGACAAAAAATAAGTTACTTT 358

||||||||||||||||||||||||||||||||||||||||||||||||||||||||||||

Sbjct 17059962 AGTCGACAGGGATGTCTACTGCAGGTGAAAACAATCCTTCGACAAAAAATAAGTTACTTT 17059903

Query 359 TGGTAAAGACAGTTAAATAATAAGCAGCTATATCACGCGCATGGGAGAATTGCATATTCA 418

||||||||||||||||||||||||||||||||||||||||||||||||||||||||||||

Sbjct 17059902 TGGTAAAGACAGTTAAATAATAAGCAGCTATATCACGCGCATGGGAGAATTGCATATTCA 17059843

Query 419 ATTACAATCATTAtttttttttCAGAACACTGTCGGCGACATTGAGAATTAATCTACCCG 478

||||||||||||||||||||||||||||||||||||||||||||||||||||||||||||

Sbjct 17059842 ATTACAATCATTATTTTTTTTTCAGAACACTGTCGGCGACATTGAGAATTAATCTACCCG 17059783

Query 479 TGCAAACAAAGAACAGAGAAACTATAGTATACCTACATGTATCTATCACCCAATAATTGC 538

||||||||||||||||||||||||||||||||||||||||||||||||||||||||||||

Sbjct 17059782 TGCAAACAAAGAACAGAGAAACTATAGTATACCTACATGTATCTATCACCCAATAATTGC 17059723

Query 539 AAGATCATGTTACAAAACGGTTCTAATTAATATATAGAAACAAGGCAGAGAATTCTACCT 598

||||||||||||||||||||||||||||||||||||||||||||||||||||||||||||

Sbjct 17059722 AAGATCATGTTACAAAACGGTTCTAATTAATATATAGAAACAAGGCAGAGAATTCTACCT 17059663

Query 599 TTCTTTTGTCTAAGTACAATTATCTTTTTCTCCGCGATTAATATTTTTCGAGTAGTAAAA 658

||||||||||||||||||||||||||||||||||||||||||||||||||||||||||||

Sbjct 17059662 TTCTTTTGTCTAAGTACAATTATCTTTTTCTCCGCGATTAATATTTTTCGAGTAGTAAAA 17059603

Query 659 TTTAAGTCAAAAGCCGTATCAGGATTCAGGAATAATCCTTCACTGGGAGAGATCTCATGT 718

||||||||||||||||||||||||||||||||||||||||||||||||||||||||||||

Sbjct 17059602 TTTAAGTCAAAAGCCGTATCAGGATTCAGGAATAATCCTTCACTGGGAGAGATCTCATGT 17059543

Query 719 GATTTGCTGTTGCACTCGGCGGCTATCTTTTACCGTTCCCAGCAGGAAGCTGCAGACGTT 778

||||||||||||||||||||||||||||||||||||||||||||||||||||||||||||

Sbjct 17059542 GATTTGCTGTTGCACTCGGCGGCTATCTTTTACCGTTCCCAGCAGGAAGCTGCAGACGTT 17059483

Query 779 GGAGAGATCGATCTCTACTGACAATGCACAAAGCAATTACTCACTAAATTGGCTATGGCT 838

||||||||||||||||||||||||||||||||||||||||||||||||||||||||||||

Sbjct 17059482 GGAGAGATCGATCTCTACTGACAATGCACAAAGCAATTACTCACTAAATTGGCTATGGCT 17059423

Query 839 AGTGAGAGGTGCGCTGCGCACAAAGCCAATGCAACtttttttGAAAATTAGCCAGGATTA 898

||||||||||||||||||||||||||||||||||||||||||||||||||||||||||||

Sbjct 17059422 AGTGAGAGGTGCGCTGCGCACAAAGCCAATGCAACTTTTTTTGAAAATTAGCCAGGATTA 17059363

Query 899 TCTCCAACAGTAGCTCATTTTTGTAAAAGCCTAATTATTGTGCGTGTCCAAAAGACTTTC 958

||||||||||||||||||||||||||||||||||||||||||||||||||||||||||||

Sbjct 17059362 TCTCCAACAGTAGCTCATTTTTGTAAAAGCCTAATTATTGTGCGTGTCCAAAAGACTTTC 17059303

Query 959 CTCAAAAGCAAATAAAGaaaaaaaaTCTTTGCATAATTATTCTATGATTACTTTGATGCG 1018

||||||||||||||||||||||||||||||||||||||||||||||||||||||||||||

Sbjct 17059302 CTCAAAAGCAAATAAAGAAAAAAAATCTTTGCATAATTATTCTATGATTACTTTGATGCG 17059243

Query 1019 TACGTGAATGGCCATGGGTAGGAGGCAACCAAGTGATTCGCACCTAGCTAGCTTTTCTCC 1078

||||||||||||||||||||||||||||||||||||||| ||||||||||||||| ||||

Sbjct 17059242 TACGTGAATGGCCATGGGTAGGAGGCAACCAAGTGATTCCCACCTAGCTAGCTTTGCTCC 17059183

Query 1079 TATAT-AAGCACCACAACTCCCTTCATTCCTCTCCAAGAGTTTTCAGCCAACACATTGAA 1137

||||| ||||||||||||||||||||||||||||||||||||||||||||||||||||||

Sbjct 17059182 TATATAAAGCACCACAACTCCCTTCATTCCTCTCCAAGAGTTTTCAGCCAACACATTGAA 17059123

Query 1138 CTCTTCTTCAGAGCTCTCCCTTCCCTCCACAAA-GGGGTCTAGGGTTAGAGTGTGTGTGT 1196

||||||||||||||||||||||||||||||||| ||||||||||||||||||||||||||

Sbjct 17059122 CTCTTCTTCAGAGCTCTCCCTTCCCTCCACAAAGGGGGTCTAGGGTTAGAGTGTGTGTGT 17059063

Query 1197 CTGTGACAAGTTCCAAGCTAGCAACAACAAGCTCAATTCCTTGCTTGTTTGCTTCCATAT 1256

||||||||||||||||||||||||||||||||||||||||||||||||||||||||||||

Sbjct 17059062 CTGTGACAAGTTCCAAGCTAGCAACAACAAGCTCAATTCCTTGCTTGTTTGCTTCCATAT 17059003

Query 1257 TACACTACATCTCTTCCCTTCAATTACCCCCCTTTTAGCACACAAAAATGGCTGGCCTGT 1316

||||||||||||||||||||||||||||||||||||||||||||||||||||||||||||

Sbjct 17059002 TACACTACATCTCTTCCCTTCAATTACCCCCCTTTTAGCACACAAAAATGGCTGGCCTGT 17058943

Query 1317 CCCTGCAGCATCCCTGGGCTTTTGCCTTCGGCCTCCTTGGTATATCATCATCACCTACCA 1376

||||||||||||||||||||||||||||||||||||||||||||||||||||||||||||

Sbjct 17058942 CCCTGCAGCATCCCTGGGCTTTTGCCTTCGGCCTCCTTGGTATATCATCATCACCTACCA 17058883

Query 1377 CAACTAAGACATTCCCTTCATTGCCAACATTTTACTTCTTTTTATTAGAAACCATTGAGT 1436

||||||||||||||||||||||||||||||||||||||||||||||||||||||||||||

Sbjct 17058882 CAACTAAGACATTCCCTTCATTGCCAACATTTTACTTCTTTTTATTAGAAACCATTGAGT 17058823

Query 1437 TTGTACAT 1444

||||||||

Sbjct 17058822 TTGTACAT 17058815

**Alignment of OsSWEET14_ 888bp between Komboka and Kitaake**

>jgi:Chr11_499 OsativaKitaake|499|v3.1

Length=29738898

Score = 1579 bits (1750), Expect = 0.0

Identities = 883/888 (99%), Gaps = 0/888 (0%)

Strand=Plus/Minus

Query 1 TGCGGCTCATCAGTTTCTCTAAGCTCTCACCATTCATTCCACTATACAAGCCTAAGGCAG 60

||||||||||||||||||||||||||||||||||||||||||||||||||||||||||||

Sbjct 18398101 TGCGGCTCATCAGTTTCTCTAAGCTCTCACCATTCATTCCACTATACAAGCCTAAGGCAG 18398042

Query 61 CTAGCTTAGTTAATTACCTAATAACTATAGCTTGCCCAACTCTAGATCCCTTAACTAGGA 120

||||||||||||||||||||||||||||||||||||||||||||||||||||||||||||

Sbjct 18398041 CTAGCTTAGTTAATTACCTAATAACTATAGCTTGCCCAACTCTAGATCCCTTAACTAGGA 18397982

Query 121 CAACTTGGAGTACACAACAATGTTAATAATCCCATGCATTGAGGACAGAGTTGTGAAGGA 180

||||||||||||||||||||||||| |||||||||||||||||||||||||| |||||||

Sbjct 18397981 CAACTTGGAGTACACAACAATGTTACTAATCCCATGCATTGAGGACAGAGTTATGAAGGA 18397922

Query 181 AACaaaaaaaaGCTAGCAGATTGGCACTTTCTGTCATGCATGGGTGCTGATGATTATCTT 240

||||||||||||||||||||||||||||||||||||||||||||||||||||||||||||

Sbjct 18397921 AACAAAAAAAAGCTAGCAGATTGGCACTTTCTGTCATGCATGGGTGCTGATGATTATCTT 18397862

Query 241 GTATCTAATTTAATCAATCCCATGGCTGTGATTGATCAGGAATAGTTTGTGTGTGCAGCT 300

||||||||||||||||||||||||||||||||||||||||||||||||||||||||||||

Sbjct 18397861 GTATCTAATTTAATCAATCCCATGGCTGTGATTGATCAGGAATAGTTTGTGTGTGCAGCT 18397802

Query 301 ATATTACCTATTGGTGTCCAGGGTCACACACCATAAGGGCATGCATGTCAGCAGCTGGTC 360

||||| ||||||||||||||||||||||||||||||||||||||||||||||||||||||

Sbjct 18397801 ATATTGCCTATTGGTGTCCAGGGTCACACACCATAAGGGCATGCATGTCAGCAGCTGGTC 18397742

Query 361 ATGTGTGCCTTTTCATTCCCTTCTTCCTTCCTAGCACTATATAAACCCCCTCCAACCAGG 420

||||||||||||||||||||||||||||||||||||||||||||||||||||||||||||

Sbjct 18397741 ATGTGTGCCTTTTCATTCCCTTCTTCCTTCCTAGCACTATATAAACCCCCTCCAACCAGG 18397682

Query 421 TGCTAAGCTCATCAAGCCTTCAAGCAAAGCAAACTCAAGTAGTAGCTGATTACCAGCTCT 480

||||||||||||||||||||||||||||||||||||||||||||||||||||||||||||

Sbjct 18397681 TGCTAAGCTCATCAAGCCTTCAAGCAAAGCAAACTCAAGTAGTAGCTGATTACCAGCTCT 18397622

Query 481 TCTCTCTTCTCATTGAGAAGAGGGAATTAAGTTTTGATCTCTGCTTTATTGCCTGATCAT 540

||||||||||||||||||||||||||||||||||||||||||||||||||||||||||||

Sbjct 18397621 TCTCTCTTCTCATTGAGAAGAGGGAATTAAGTTTTGATCTCTGCTTTATTGCCTGATCAT 18397562

Query 541 CCTCTTGTTACTTGCAAGCAAGAACAGTAGTGTACTGTGCCTCATTGATCTCCTCTCACC 600

||||||||||||||||||||||||||||||||||||||||||||||||||||||| ||||

Sbjct 18397561 CCTCTTGTTACTTGCAAGCAAGAACAGTAGTGTACTGTGCCTCATTGATCTCCTCCCACC 18397502

Query 601 AAActctctctctctctctcATATTCCGAGCTAGCTAGTTAATCAAGATCTTGCTGCAAT 660

||||||||||||||||||||||||||||||||||||||||||||||||||||||||||||

Sbjct 18397501 AAACTCTCTCTCTCTCTCTCATATTCCGAGCTAGCTAGTTAATCAAGATCTTGCTGCAAT 18397442

Query 661 GGCTGGCATGTCTCTTCAGCATCCCTGGGCCTTCGCCTTTGGTCTCCTAGGTGTGTTGCC 720

||||||||||||||||||||||||||||||||||||||||||||||||||||||||||||

Sbjct 18397441 GGCTGGCATGTCTCTTCAGCATCCCTGGGCCTTCGCCTTTGGTCTCCTAGGTGTGTTGCC 18397382

Query 721 TTTGATCTGATCCAAGGAATTCTCTTGAGAATTAATCTTGCATGGTTATTTACTTTTGTT 780

||||||||||||||||||||||||||||||||||||||||||||||||||||||||||||

Sbjct 18397381 TTTGATCTGATCCAAGGAATTCTCTTGAGAATTAATCTTGCATGGTTATTTACTTTTGTT 18397322

Query 781 GTTATTATTCTCTACATTTTTAATCATGTACTTTTCCATGTTCCTCTTTTGTTGCCAAAG 840

|||||||||||||||||||||||||||||||||||||||||||| |||||||||||||||

Sbjct 18397321 GTTATTATTCTCTACATTTTTAATCATGTACTTTTCCATGTTCCACTTTTGTTGCCAAAG 18397262

Query 841 CTACTATATTTTTCCTACCAATTCATCCAAAACTACTATATTATAGCA 888

||||||||||||||||||||||||||||||||||||||||||||||||

Sbjct 18397261 CTACTATATTTTTCCTACCAATTCATCCAAAACTACTATATTATAGCA 18397214
